# HIV-1 Nef generates lasting innate immune memory in haematopoietic stem and progenitor cells in vivo

**DOI:** 10.1038/s44319-026-00838-w

**Published:** 2026-06-15

**Authors:** Andrew J Fleetwood, Nigora Mukhamedova, Dragana Dragoljevic, Man K S Lee, Yangsong Xu, Malathi S I Dona, Ian Hsu, Camilla Bertuzzo Veiga, Fumihiko Takeuchi, Ben Crossett, Denise Tran, Alexander R Pinto, Michael Bukrinsky, Andrew J Murphy, Dmitri Sviridov

**Affiliations:** 1 Baker Heart and Diabetes Institute, Melbourne, VIC Australia; 2 Baker Department of Cardiometabolic Health, University of Melbourne, Melbourne, VIC Australia; 3 School of Agriculture, Biomedicine and Environment, Baker Department of Cardiovascular Research, Translation and Implementation, La Trobe University, Bundoora, VIC Australia; 4 Sydney Mass Spectrometry, The University of Sydney, Camperdown, NSW Australia; 5 Department of Microbiology, Immunology and Tropical Diseases, George Washington University, Washington DC, USA; 6 Department of Biochemistry and Molecular Biology, Monash University, Clayton, VIC Australia

**Keywords:** Immunology, Microbiology, Virology & Host Pathogen Interaction, Stem Cells & Regenerative Medicine

## Abstract

HIV infection is accompanied by chronic inflammation-related co-morbidities, even when viral replication is suppressed by therapy. This persistent inflammatory state suggests that long-lived immune cell lineages may acquire stable pro-inflammatory programming. Here, we investigate whether inflammatory programming can be imprinted within hematopoietic lineages, following the exposure of mice and bone marrow-derived macrophages (BMDMs) to extracellular vesicles (EVs) carrying Nef, a key inflammatory factor of HIV. Multi-omics profiling shows that hematopoietic cells exposed to Nef-EVs undergo epigenetic remodeling and reprogramming of energy and lipid metabolism characteristic of trained innate immunity. The inflammatory phenotype in BMDMs is partially reversed by inhibition of glycolysis, a key metabolic driver of trained immunity. We demonstrate that following competitive bone marrow transplantation, hematopoiesis in mice receiving bone marrow from Nef-EV-treated donors displays a sustained bias toward myelopoiesis, and BMDMs retain enhanced inflammatory potential. These findings demonstrate that Nef-EVs can imprint a lasting inflammatory memory, mechanistically similar to trained immunity, in hematopoietic cells. This memory persists beyond the initial exposure and may contribute to chronic inflammation in people with HIV.

## Synopsis

Extracellular vesicles carrying HIV-1 protein Nef can imprint an inflammatory memory, similar to trained immunity, in haematopoietic cells. This memory persists beyond the initial exposure and may contribute to chronic inflammation in people with HIV.

- Extracellular vesicles carrying HIV protein Nef can imprint an inflammatory memory into haematopoietic cells in vivo.
- This memory is mechanistically similar to trained immunity.
- This memory persists beyond the initial exposure and may contribute to chronic inflammation accompanying HIV infection.
- This may contribute to the pathogenesis of HIV-associated co-morbidities and persist even after a functional cure of HIV.

## Abstract (web summary)

Extracellular vesicles carrying HIV-1 protein Nef can imprint an inflammatory memory, similar to trained immunity, in haematopoietic cells. This memory persists beyond the initial exposure and may contribute to chronic inflammation in people with HIV.

## Graphical abstract

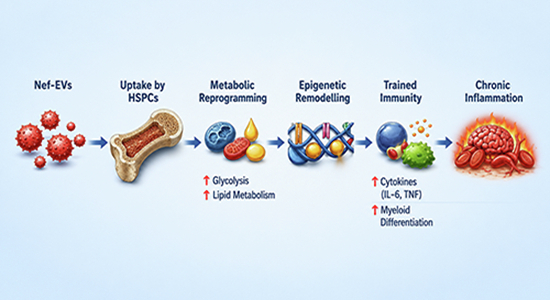

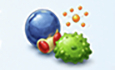

## Introduction

HIV infection is accompanied by numerous cardiovascular, metabolic, and neurological co-morbidities, such as atherosclerosis, diabetes, and HIV-associated neurocognitive disorders (HAND) (Perkins et al, 2023; Sviridov and Bukrinsky, 2023; Sviridov et al, 2020). Current antiretroviral regimens effectively eliminate the virus from circulation and reverse immunodeficiency; however, cardiometabolic and neurological co-morbidities persist, albeit often in a milder form (Triant et al, 2018). A common feature of these co-morbidities, frequently observed in people living with HIV (PLWH) receiving antiretroviral therapy, is chronic systemic inflammation.

A significant contributor to chronic inflammation in PLWH is the HIV-1 protein Nef (Mukhamedova et al, 2019), which is synthesized in infected cells in viral reservoirs. Nef promotes the secretion of inflammatory cytokines by these cells; moreover, Nef itself is incorporated into EVs released from the infected cells into systemic as well as brain circulation (Aiello et al, 2021; Lavrin et al, 2025; McNamara et al, 2018; Puzar Dominkus et al, 2017). These EVs are released independently of viral particle production (Lenassi et al, 2010); they contain Nef both as a cargo and on the surface of the EV (Vanpouille et al, 2024) and affect the metabolism of bystander cells (Arenaccio et al, 2014a; Khan et al, 2016). Specifically, Nef and Nef-EVs reduce the abundance of the cellular cholesterol transporter ABCA1 and enhance the abundance of plasma membrane lipid rafts across multiple cell types and tissues (Mujawar et al, 2006; Mukhamedova et al, 2019). Both effects contribute to the pathogenesis of chronic inflammation, acting independently as well as interdependently (Fitzgerald et al, 2010; Varshney et al, 2016). Furthermore, in the infected cells Nef alters the composition of the EV cargo secreted by these cells, which might affect bystander cells independently of the direct effects of Nef (da Silva-Januario et al, 2023).

Although experimental strategies targeting the Nef-ABCA1-lipid rafts axis have shown promise (Adzhubei et al, 2021; Dubrovsky et al, 2020; Hunegnaw et al, 2016; Ramezani et al, 2015), the only radical therapeutic approach capable of eliminating persistent inflammation in PLWH seems to be complete eradication of the virus and its genetic material from the body, a true “cure” for HIV (Chou et al, 2024). In theory, eliminating the primary cause of inflammation should lead to its resolution and, subsequently, to the amelioration of HIV-associated co-morbidities. This assumption, however, has been challenged by findings showing that monocytes exposed to Nef-EVs (Dubrovsky et al, 2022), as well as uninfected monocytes from the blood of PLWH (van der Heijden et al, 2021), remain hyperresponsive to inflammatory stimuli even in the absence of direct encounter with HIV-related pathogenic factors. If confirmed, these findings would imply that chronic inflammation and associated metabolic and neurological co-morbidities may persist even after patients are “virus-free”. This phenomenon parallels the concept of innate immune priming or trained immunity.

Trained innate immunity (TRIM) refers to the capacity of the innate immune system to develop non-specific immunologic memory through metabolic and epigenetic reprogramming of immune cells (Bekkering et al, 2018; Fanucchi et al, 2021; Hajishengallis et al, 2023; Mitroulis et al, 2018; Netea et al, 2011; Noz et al, 2020). Initially described for β-glucan (Saeed et al, 2014) and BCG vaccine (Moorlag et al, 2024), TRIM has since been observed following exposure to a variety of stimuli, including viral infections (Taks et al, 2022). While TRIM provides an additional layer of protection against reinfection (Quintin et al, 2012), maladaptive TRIM can drive sustained inflammatory activation, contributing to chronic inflammation and associated co-morbidities long after the initial infection has been resolved (Li et al, 2022). Such mechanisms have been implicated in post-acute sequelae of viral infections, including COVID-19 (Cheong et al, 2023; Gu et al, 2023). Based on these observations, we investigated whether HIV, through the activity of its Nef protein, promotes the establishment of a durable, myeloid-biased state of innate immune memory consistent with trained immunity.

## Results

### EVs from SupT1 cells

**Figure EV1:**
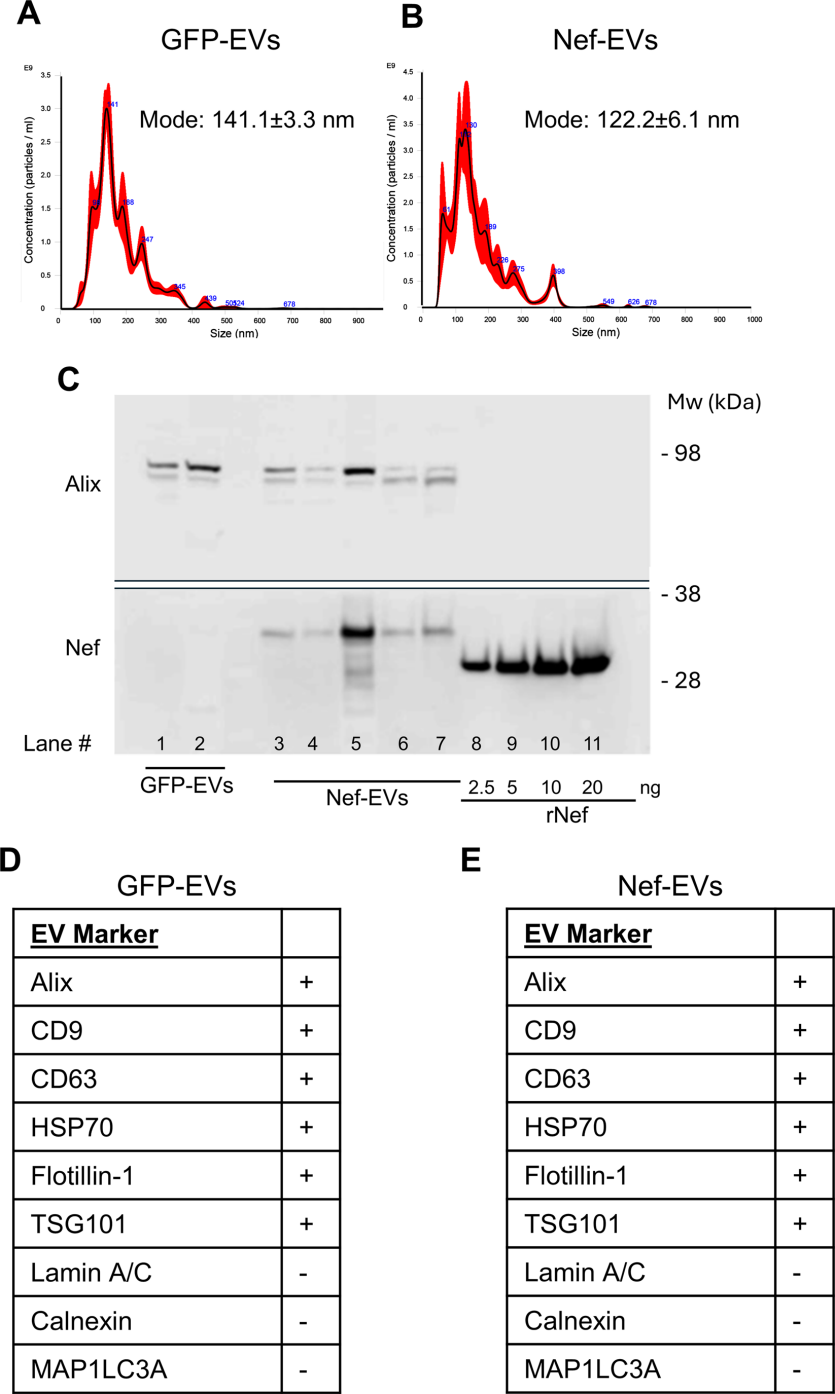
Characterization of EVs produced by SupT1 cells. (**A**, **B**) Size distribution of GFP-EVs (**A**) and Nef-EVs (**B**). (**C**) Western blot for assessing Nef content in Nef-EVs. Lane 1—GFP-EVs, 5 μg total protein, lane 2—GFP-EVs, 10 μg total protein, lane 3—Nef-EVs produced in HEK293 cells (10^9^ particles), lane 4—Nef-EVs produced in HEK293 cells (0.5 × 10^9^ particles), lane 5—Nef-EVs produced in SupT1 cells (0.4 × 10^10^ particles), lane 6—Nef-EVs produced in SupT1 cells (1 × 10^9^ particles), lane 7—Nef-EVs produced in SupT1 cells (0.5 × 10^9^ particles, from a different experiment), lanes 8–11—rNef (indicated amounts). (**D**, **E**) EV markers discovered during proteomics analysis of GFP-EVs (**D**) and Nef-EVs (**E**). Source data are available online for this figure.

We have previously reported inflammatory effects of Nef-containing EVs (Nef-EVs) on macrophages (Dubrovsky et al, 2022; Mukhamedova et al, 2019). A limitation of that study was that EVs were produced in HEK293 cells, a cell type not involved in HIV infection. To overcome this limitation, in this study we used EVs produced in SupT1 cells, a cell line of human CD4 + T cells, a cell type carrying the bulk of HIV load. Details of isolation of EVs carrying Nef or green fluorescent protein (GFP-EVs, control EVs) are provided in the Methods section. The predominant size of Nef-EVs and GFP-EVs was, respectively, 122 ± 6 nm and 141 ± 3 nm (Fig. EV1A,B). Both types of EVs contained the EV marker Alix (Fig. EV1C, lanes 1–7), and Nef-EVs contained Nef (Fig. EV1C, lanes 5–7) while no Nef was detected in GFP-EVs (Fig. EV1C, lanes 1–2). The concentration of Nef in Nef-EVs was evaluated by densitometric analysis comparing the bands of Nef-EVs with titrated recombinant Nef (Fig. EV1C, lanes 8–11) and was usually ~1 ng of Nef per 10^9^ particles. The EVs were further characterized by the identification of EV markers in the proteomics dataset of GFP-EVs and Nef-EVs (see below). Six biomarkers of EVs recommended by ISEV (Welsh et al, 2024), were identified in both GFP-EVs and Nef-EVs, while markers of nucleus (Lamin), endoplasmic reticulum (Calnexin) and microtubules (MAP1LC3A) were not found (Fig. EV1D,E).

We assumed that the effects of the infusion of Nef-EVs could be ascribed to Nef; however, it could not be excluded that the transfection of SupT1 cells with Nef resulted in the incorporation of an unidentified factor(s) into Nef-EVs, which was absent in GFP-EVs and was the true cause of the observed effects. To resolve this uncertainty, we conducted an unbiased proteomics analysis of Nef-EVs and compared proteins with control EVs. The list of proteins found in Nef-EVs, but not in the control EVs, is shown in Dataset EV1. Functional annotation of these proteins revealed that out of 110 proteins uniquely found in Nef-EVs, only 4 were involved in innate immunity pathways: Myd88, TBK1, TRAF6, and IFNG. It is important to recognize that these are human proteins, and as was shown for IFNG, they have high species specificity and are unlikely to affect mouse cells (Kumar et al, 1989).

### Nef promotes myelopoiesis

**Figure 1:**
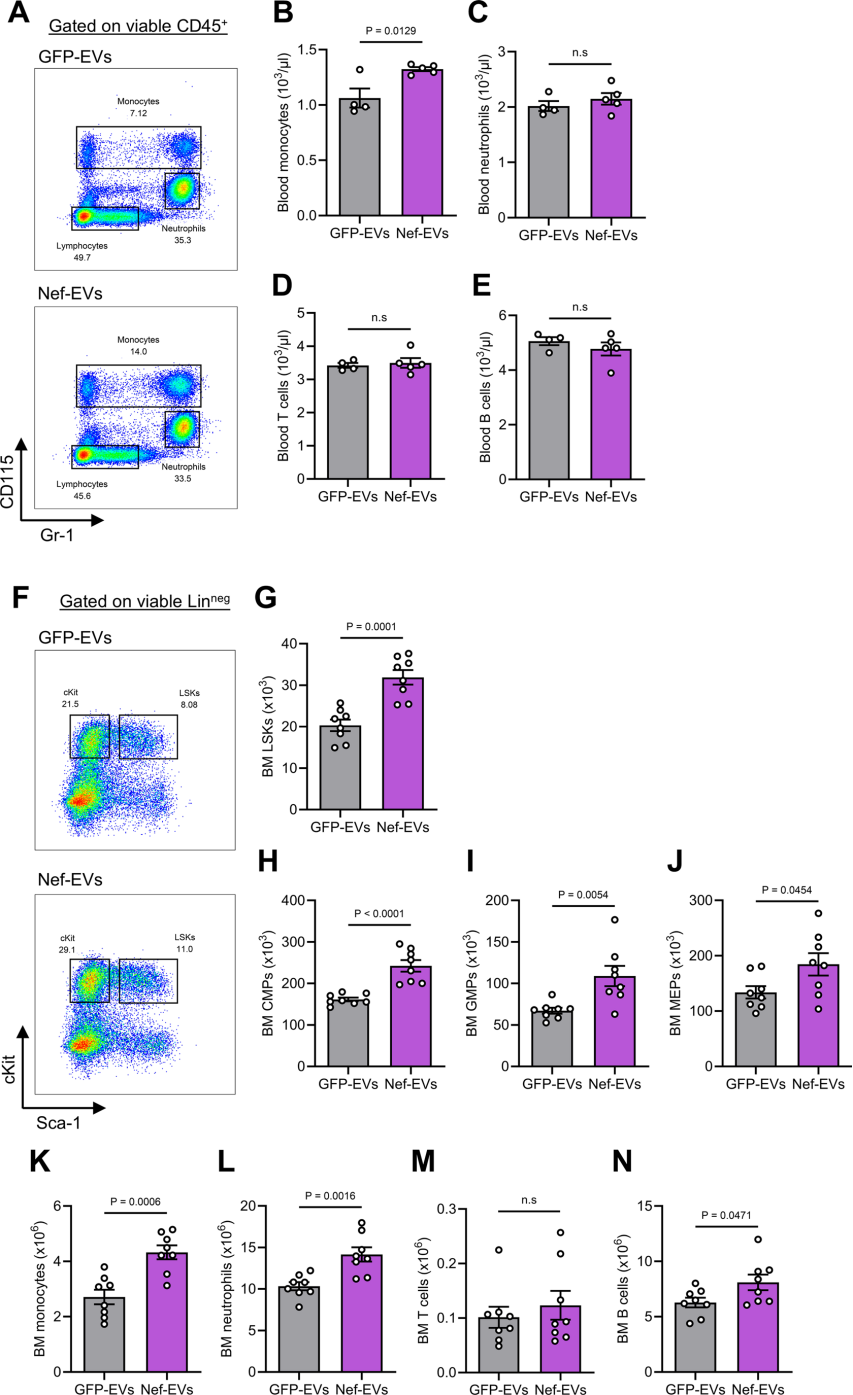
Nef-EVs promote myelopoiesis and monocytosis in mice. (**A**) Representative fluorescence-activated cell sorting (FACS) plots for the identification of monocytes and neutrophils in the blood of mice after a 2-week treatment protocol with GFP-EVs or Nef-EVs. After gating for viable CD45^+^ cells, neutrophils were characterized as CD115^lo^Ly6-C/G^hi^ (Gr-1) and monocytes were identified as CD45^+^CD115^hi^Gr-1^hi/lo^. After gating on lymphocytes (CD115^neg^Gr-1^neg^), T cells (CD3^+^) and B cells (B220^+^) were characterized (gating not depicted). (**B**–**E**) Absolute number of (**B**) blood monocytes, (**C**) neutrophils, (**D**) T cells, and (**E**) B cells in the blood of mice after GFP-EV or Nef-EV treatment (GFP-EVs, *n* = 4; Nef-EVs, *n* = 5). (**F**) Representative FACS plots for the identification of hematopoietic progenitor cells in the BM of mice after a 2-week treatment protocol with GFP-EVs or Nef-EVs. After gating for viable Lin^neg^ cells, LSK cells were characterized as cKit^+^Sca1^+^ cells. Within the cKit^+^Sca1^neg^ population, CMPs (Lin^−^c-Kit^+^Sca1^−^CD16/32^−^CD34^+^), GMPs (Lin^−^c-Kit^+^Sca1^−^CD16/32^+^CD34^+^) and MEPs (Lin^−^c-Kit^+^Sca1^−^CD16/32^−^CD34^+^) were identified. (**G**–**J**) Absolute number of (**G**) LSKs, (**H**) CMPs, (**I**) GMPs, and (**J**) MEPs in the BM of mice after GFP-EV or Nef-EV treatment (GFP-EVs, *n* = 8; Nef-EVs, *n* = 8). (**K**–**N**) Absolute number of (**K**) monocytes, (**L**) neutrophils, (**M**) T cells, and (**N**) B cells in the BM of mice after GFP-EV or Nef-EV treatment (GFP-EVs, *n* = 8; Nef-EVs, *n* = 8). Data are presented as mean ± SEM. Data points represent individual mice. Significance was determined by two-tailed Student’s *t* test with calculated *P* values show (**B**–**E**, **G**–**N**); n.s., not significant (*P* ≥ 0.05). Source data are available online for this figure.

**Figure EV2:**
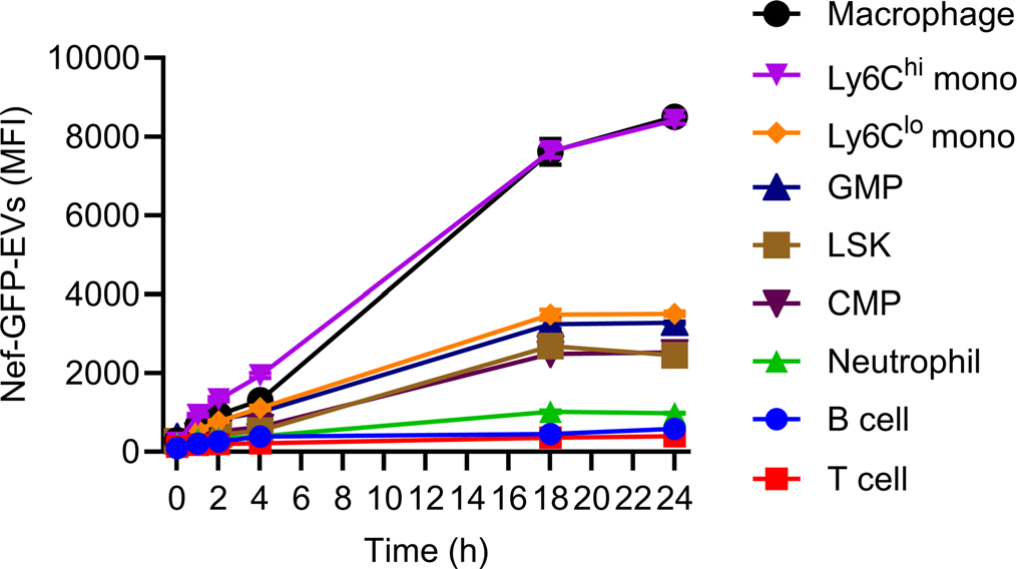
Uptake of Nef-EVs by bone marrow cells in vitro. Bone marrow freshly isolated from mice was incubated for the indicated periods of time with EVs produced in SupT1 cells transfected with GFP-Nef conjugate (4 × 10^12^ EVs). The relative amounts of GFP-Nef in different BM cell populations were detected by flow cytometry. Data presented as mean ± SEM from three biological replicates. Source data are available online for this figure.

Chronic inflammation underlies many co-morbidities associated with well-controlled HIV infection, and our previous work suggests that this may be driven, at least in part, by the viral protein Nef (Dubrovsky et al, 2022; Mukhamedova et al, 2019). Because hematologic alterations are frequently linked to chronic inflammation, we sought to determine how Nef affects hematopoietic components of the innate immune response. To this end, C57BL/6 mice were intravenously infused with EVs containing either Nef (Nef-EVs) or GFP (GFP-EVs; control) three times per week for 2 weeks. We injected 10^9^ EVs, containing ~1 ng of Nef, which corresponds to an estimated initial plasma Nef concentration of approximately 0.8 ng/mL, which is below the levels of Nef detected in the plasma Nef levels reported in PLWH, 6–12 ng/mL (Ferdin et al, 2018; Raymond et al, 2011). The dosage and regimen were based on the results of our previous study (Mukhamedova et al, 2019). Consistent with our previous observations (Mukhamedova et al, 2019), the only hematological effect of 2-week treatment with Nef-EVs was a mild monocytosis (Fig. 1A,B). The abundance of the other major leukocyte subsets, neutrophils, T and B cells, remained unchanged (Fig. 1C–E). To determine whether the elevated levels of circulating monocytes reflected altered hematopoiesis, we analyzed hematopoietic stem and progenitor cell (HSPC) subsets within the bone marrow (BM). Indeed, we observed a broad increase in various progenitor cell types following exposure to Nef-EVs. Specifically, we noted an increase in the abundance of LSK cells (LSKs; Lin^neg^ cKit^+^Sca1^+^) (Fig. 1F,G), common myeloid progenitors (CMP; Lin^neg^ cKit^+^Sca1^–^CD16/32^low^CD34^+^) (Fig. 1H), granulocyte-monocyte progenitors (GMP; Lin^neg^ cKit^+^Sca1^–^CD16/32^+^CD34^+^) (Fig. 1I), and megakaryocyte-erythroid progenitors (MEP; Lin^neg^ cKit^+^Sca1^–^CD16/32^–^CD34^–^) (Fig. 1J). Levels of mature monocytes (Fig. 1K) and neutrophils (Fig. 1L) were also increased in the BM of mice exposed to Nef-EVs. In contrast, T cell abundance remained unchanged (Fig. 1M), and B cells were only modestly increased (Fig. 1N). (Kumar et al, 1989). To investigate uptake of Nef-EVs by the different cell types present in the marrow, we incubated BM with GFP-tagged Nef-EVs (Nef-GFP-EVs) as described previously (Mukhamedova et al, 2019) and measured cellular uptake over time by flow cytometry. As expected, monocytes and macrophages were the main targets for Nef-EVs uptake, followed by the myeloid progenitors GMP and CMP, as well as LSK (Fig. EV2). Both T and B cells were only weakly positive for Nef-GFP-EV. With the exception of neutrophils, the preferential uptake of Nef-GFP-EV by mature myeloid cells and their progenitors is consistent with the broad expansion of these subsets observed in the BM following challenge with Nef-EVs. This is consistent with the findings of Kfoury et al (Kfoury et al, 2021) showing that EVs reach and deliver their cargo to BM cells, and that functional response from BM accurately reflects this delivery.

**Figure EV3:**
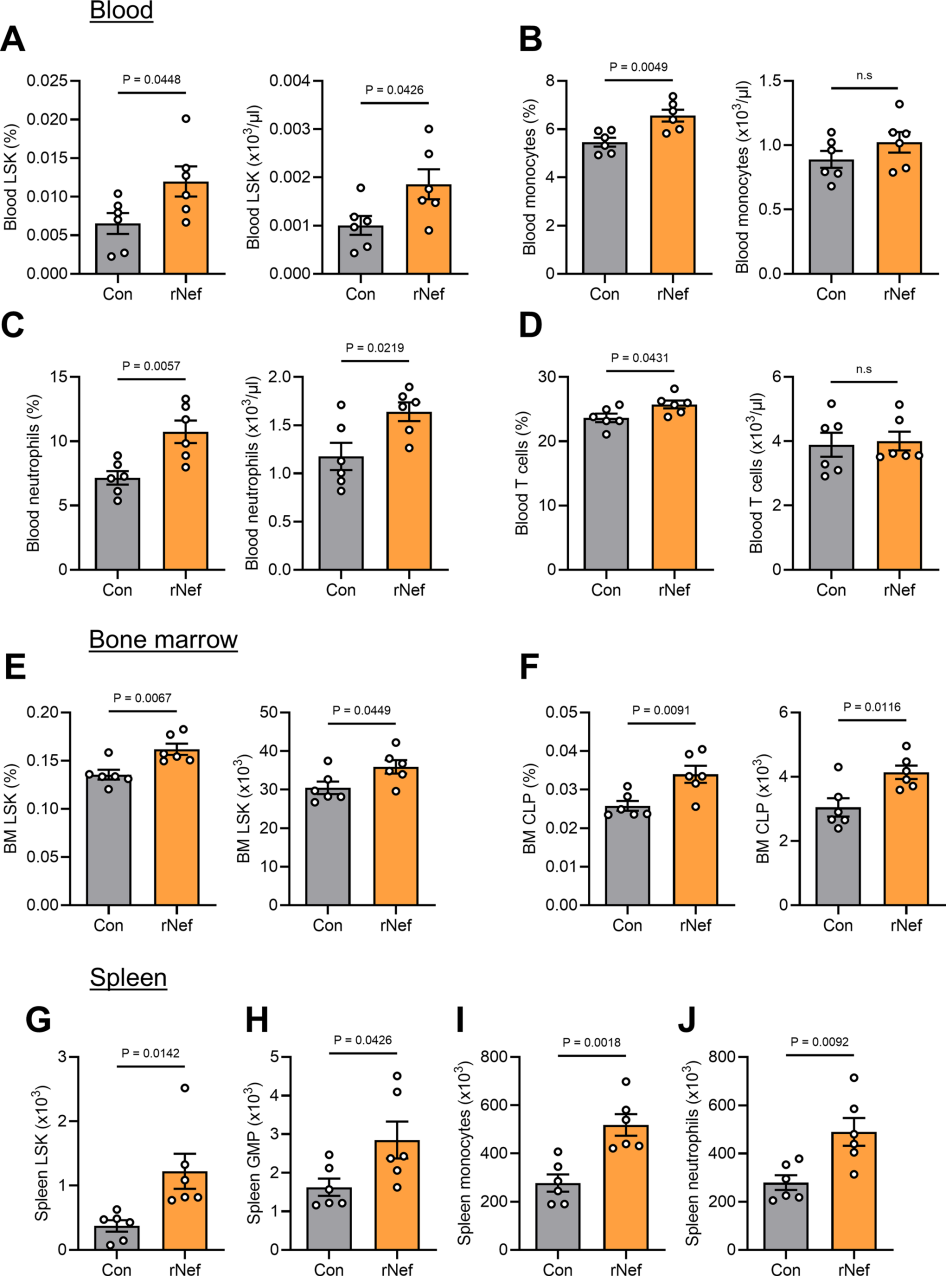
Monocytosis and myeloid progenitor expansion in the blood, bone marrow, and spleen of mice treated with recombinant Nef. (**A**–**D**) Absolute and relative abundance of LSKs (**A**), monocytes (**B**), neutrophils (**C**), and T cells (**D**), and in the blood of mice after treatment with rNef (50 ng per injection every second day for 8 days) or vehicle. (**E**,** F**) Absolute and relative abundance of LSKs (**E**) and CLP (**F**) in the bone marrow of mice after treatment with rNef (50 ng per injection every second day for 8 days) or vehicle. (**G**–**J**) Absolute numbers of LSKs (**G**), GMP (**H**), monocytes (**I**), and neutrophils (**J**) in the spleen of mice after treatment with rNef (50 ng per injection every second day for 8 days) or vehicle. Data are presented as mean ± SEM. (**A**–**J**: GFP-EVs, *n* = 6; Nef-EVs, *n* = 6). Data points represent individual mice. Significance was determined by a two-tailed unpaired Student’s *t* test (**A**–**J**) with calculated *P* values shown; n.s., not significant (*P* ≥ 0.05). Source data are available online for this figure.

To further confirm the role of Nef in the initiation of inflammation, we injected mice with naked recombinant Nef, instead of Nef-EVs. The changes in the cellular profile of blood and spleen were qualitatively similar to those observed with Nef-EVs, whereas in the BM, the response was limited to LSK and CLP cells. It must be noted that the effects were of lower magnitude and required a 50-fold higher concentration of Nef to be injected to see any effects (Fig. EV3). We have previously demonstrated that recombinant Nef affects cellular lipid metabolism (Mukhamedova et al, 2019) and triggers the formation of TRIM (Dubrovsky et al, 2022) in vitro, similar to Nef-EVs, but requires much higher concentrations to do so. Collectively, these findings support the suggestion that the consequences of the injection of Nef-EVs into mice can be predominantly attributed to Nef.

The hematologic profile after injection of Nef-EVs is consistent with activation of the innate immune system and resembles the hematopoietic remodeling observed in pristane-induced TRIM in murine BM (Kaufmann et al, 2018; Mills et al, 2024; Mitroulis et al, 2018). We therefore hypothesized that this Nef-induced hematopoietic phenotype may promote the development of trained immunity.

The establishment of TRIM is characterized by three key features: (i) epigenetic remodeling enriched at genes associated with myeloid lineage bias and innate immune activation (Saeed et al, 2014), (ii) enhanced cholesterol biosynthesis (Bekkering et al, 2018), and (iii) a metabolic switch from oxidative phosphorylation toward aerobic glycolysis (Cheng et al, 2014). We next examined the in vivo effects of Nef-EV treatment in mice across each of these defining features.

### Nef-EVs alter the epigenetic landscape of hematopoietic cells

**Figure 2:**
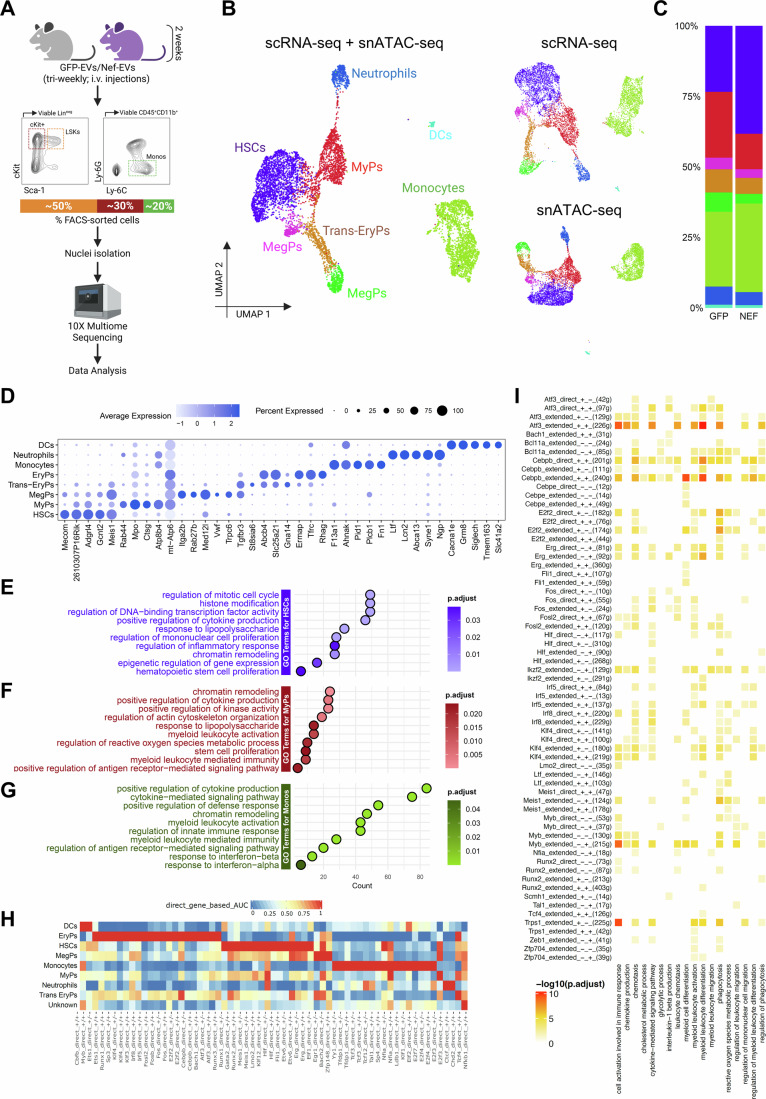
Alteration of the molecular phenotype of LSKs and monocytes in mice treated with Nef-EVs. (**A**) Experimental overview: Donor samples were used for 10X snRNA-seq and snATAC-seq. (**B**) UMAP of combined snRNA-seq and snATAC-seq data (left), snRNA-seq data alone (top right), and snATAC-seq data alone (bottom right). (**C**) Relative proportions of the different cell populations in Nef-EV and GFP-EV samples. (**D**) Top 5 distinct genes for each cell cluster determined by snRNA-seq. Dot size represents the proportion of cells expressing a given gene, and dot intensity denotes the average expression level of each gene. (**E**–**G**) Gene ontology (GO) enrichment analysis of genes that are upregulated in HSCs, Myeloid progenitor cells, and monocytes. Benjamini–Hochberg procedure was used to calculate the adjusted *P* value for each GO term. (**H**) GRN plot showing core eRegulons enriched for each cell cluster determined using the SCENIC+ pipeline. The heatmap shows TF expression of the eRegulon. (**I**) Heatmap of GO enrichment of TF target genes within eRegulons corresponding to myeloid programming-related terms. Benjamini–Hochberg procedure was used to calculate the adjusted *P* value for each GO term.

Given the myeloid bias induced by transient Nef exposure in the BM, we hypothesized that Nef directly reprograms hematopoietic stem and progenitor cells at the transcriptional and epigenetic levels. To test this scenario, we performed paired single-nucleus RNA-seq and ATAC-seq (10x Multiome) to simultaneously profile the transcriptome and chromatin accessibility in hematopoietic stem cells, myeloid progenitors, and mature myeloid cells isolated from the BM of Nef-EVs- or GFP-EVs-challenged mice. To enrich for the cell populations of interest, we used FACS to isolate LSKs (~ 50%), cKit^+^ myeloid progenitors (~ 30%), and mature myeloid cells (~ 20%), from the BM. The sorted cells were pooled, and libraries were prepared for snRNA-seq and snATAC-seq analysis (Fig. 2A).

Joint clustering after dimensionality reduction resulted in eight major populations, including hematopoietic stem cells (HSCs), myeloid progenitors (MyPs), transitional-erythroid progenitors (Trans-EryPs), erythroid progenitors (EryPs), megakaryocyte progenitors (MegPs), dendritic cells (DCs), neutrophils, and monocytes (Fig. 2B). The relative proportions of these populations differed slightly between Nef-EVs and GFP-EVs conditions (Fig. 2C), and cell type annotations were defined based on their transcriptomic signatures (Fig. 2D).

We next performed Gene Ontology (GO) enrichment analysis in HSCs, myeloid progenitors, and monocytes to compare the transcriptional profiles between Nef-EV- and GFP-EVs-treated conditions. Consistent with the myelopoiesis data (Fig. 1), differentially enriched GO terms were associated with myeloid function (Fig. 2E–G). Interestingly, we also identified GO terms associated with chromatin remodeling and innate immune responses that were differentially enriched across all three cell populations (HSCs, myeloid progenitor cells, and monocytes), specifically the terms *epigenetic regulation*, *histone modification*, and *positive regulation of cytokine production* in HSCs. These findings suggest that Nef-EVs may imprint a transcriptional memory in HSCs.

We next applied SCENIC^+^ to infer gene regulatory networks and compared eRegulon enrichment between Nef-EV- and GFP-EV-treated groups. This analysis identified the top distinct regulons associated with each cell population, highlighting lineage-defining transcription factors that define cluster identity (Fig. 2H). Since our focus was on the potential transcriptional memory within HSCs, we concentrated on this population. Notably, among the most differentially enriched eRegulons in HSCs from Nef-EV-treated mice compared with GFP-EV controls were *Klf4*, *Irf8*, and *Cebpb*, key transcription factors that regulate myeloid cell differentiation and activation (Fig. 2I). These results indicate that Nef-EVs not only promote myelopoiesis but may also transcriptionally imprint myeloid memory cues at the stem cell level. This sustained reprogramming is consistent with the manifestation of TRIM, where epigenetic and transcriptional rewiring in HSCs and myeloid progenitors enhances subsequent myeloid responses. Although we did not investigate the specific mechanism of this reprogramming, the epigenetic marks, the results of snATAC-seq analysis show that Nef has caused an epigenetic reprogramming through changes in chromatin structure/accessibility, which is a functional readout of epigenetic modification. Together, our data suggest that transient exposure to Nef-EVs may induce TRIM within HSCs, predisposing the hematopoietic system toward myeloid-biased differentiation.

### Nef-EVs modify cholesterol metabolism

**Figure 3:**
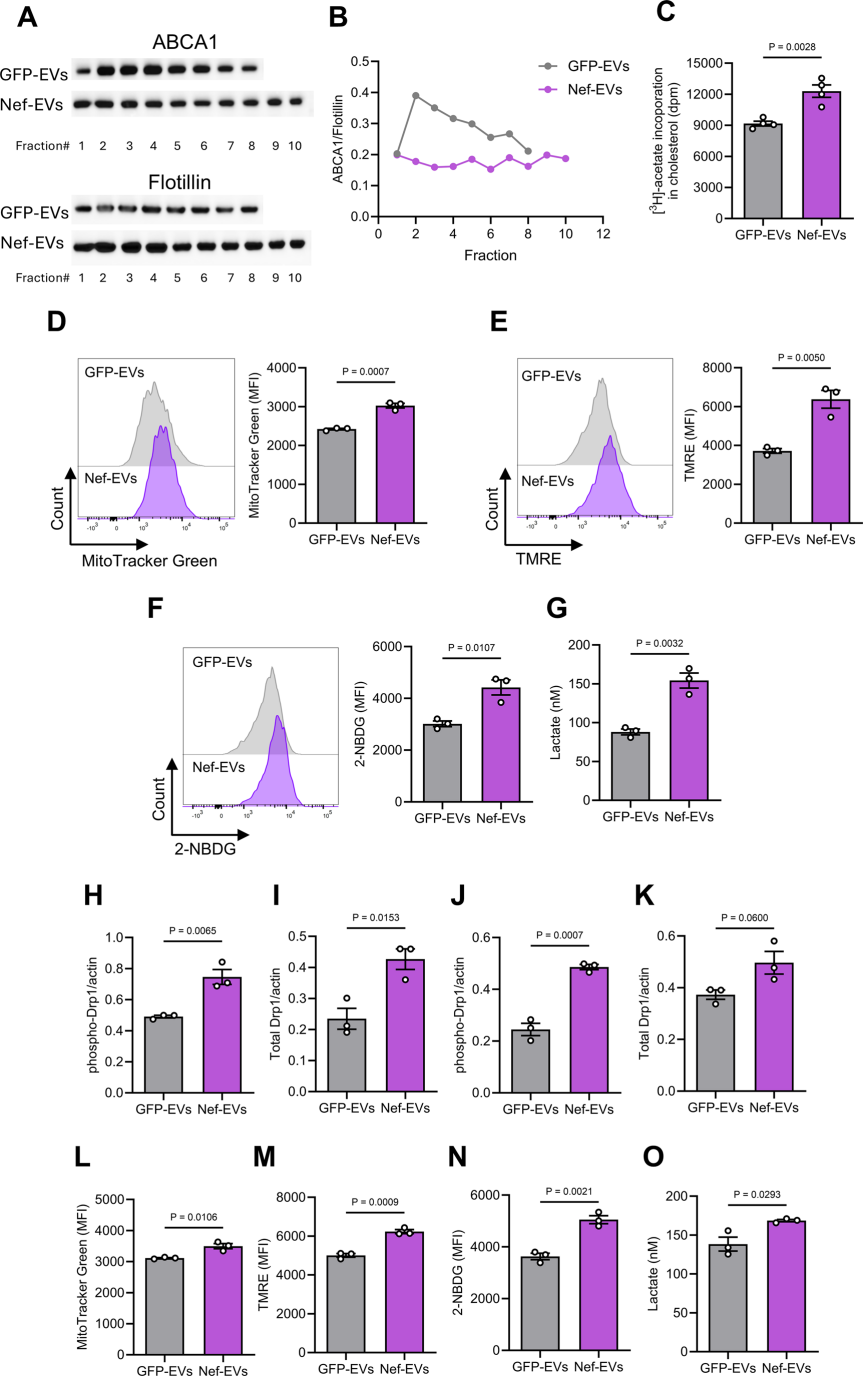
Altered energy and cholesterol metabolism in BMDM isolated from mice treated with Nef-EVs. (**A**)Western blot of ABCA1 and Flotillin in lipid raft fractions isolated from BMDM from the BM of mice treated with either GFP-EVs or Nef-EVs and grown for 7 days after isolation. (**B**) Densitometric analysis of the WB (abundance of ABCA1 relative to Flotillin) of the image shown in (**A**). (**C**) Cholesterol biosynthesis in BMDM isolated from the BM of mice treated with either GFP-EVs or Nef-EVs (0.4 × 10^9^ particles/ml) and grown for 7 days after isolation. Cells were metabolically labeled with [^3^H]acetate for 2 h and its incorporation into cholesterol was assessed as described in Methods. Mean ± SD, *t* test, *n* = 4. (GFP-EVs, *n* = 4; Nef-EVs, *n* = 4). Data points represent biological replicates. (**D**–**F**) Representative histograms and quantified median fluorescence intensity (MFI) of (**D**) MitoTracker Green, (**E**) TMRE (mitochondrial membrane potential), and (**F**) 2-NBDG (glucose uptake) in BMDM treated with GFP-EVs or Nef-EVs for 48 h in vitro. (**G**) Lactate levels in the supernatant of BMDM treated with GFP-EVs or Nef-EVs (0.4 × 10^9^ particles/ml) for 48 h in vitro. (**H**, **I**) Abundance of phosphorylated (**H**) and total (**I**) Drp1 in BMDM treated with GFP-EVs or Nef-EVs (0.4 × 10^9^ particles/ml) for 48 h. The data derived from densitometric analysis of the western blots. (**J**, **K**) Abundance of phosphorylated (**J**) and total (**K**) Drp1 in BMDM derived from bone marrow of mice treated with Nef-EVs or GFP-EVs in vivo and grown for 7 days after isolation. The data derived from densitometric analysis of the western blots. (**L**–**O**) MFI of MitoTracker Green, (**L**) TMRE, (M) 2-NBDG (**N**), and lactate release (**O**) in BMDMs treated with Nef-EVs or GFP-EVs (0.4 × 10^9^ particles/ml) for 48 h in vitro, then washed and rested for an additional 5 days. Data are presented as mean ± SEM. (**D**–**O**: GFP-EVs, *n* = 3; Nef-EVs, *n* = 3). Data points represent biological replicates. Significance was determined by two-tailed Student’s *t* test with calculated *P* values shown (**C**–**O**). Source data are available online for this figure.

The acute effect of Nef-EVs on macrophages has been shown to be mediated by a reduction in the abundance of cholesterol transporter ABCA1 (Cui et al, 2012; Dubrovsky et al, 2022; Mujawar et al, 2006; Mukhamedova et al, 2019). Consistent with this, we confirmed that ABCA1 levels in the lipid rafts were lower in macrophages derived from the BM of Nef-EV-treated mice compared with those derived from GFP-EV-treated control animals (Fig. 3A,B).

Another effect of Nef on cholesterol metabolism is stimulation of cholesterol biosynthesis (Dubrovsky et al, 2022; van ‘t Wout et al, 2005; Zheng et al, 2003), which is particularly relevant given the crucial role of cholesterol biosynthesis in the formation of trained immunity (Bekkering et al, 2018). We found that the rate of cholesterol biosynthesis in macrophages derived from the BM of mice treated with Nef-EVs was elevated (Fig. 3C). These findings indicate that Nef-EVs reprogram cholesterol homeostasis in macrophages in a manner consistent with the establishment of TRIM.

### Energy metabolism is altered in cells treated with Nef-EVs

To characterize the impact of Nef-EVs on energy metabolism, we profiled mitochondrial function and glycolysis in macrophages. For this purpose, BMDMs were treated in vitro with Nef-EVs or control GFP-EVs (0.4 × 10^9^ particles/ml) for 48 h (Mukhamedova et al, 2019) and subsequently stained with MitoTracker Green and TMRE to assess total mitochondrial content and membrane potential, respectively. We found that Nef-EVs increased both mitochondrial mass and membrane potential, indicating elevated mitochondrial respiration (Fig. 3D,E). This was accompanied by increased glucose uptake and lactate production following Nef-EV treatment (Fig. 3F,G). Treatment of BMDMs with Nef-EVs elevated the abundance of both total and phosphorylated Drp1, indicating increased mitochondrial fission (Fig. 3H,I), another hallmark of TRIM (Ding et al, 2023). Importantly, macrophages derived from the BM of Nef-EV-treated mice also displayed elevated abundance of phosphorylated Drp1 (Fig. 3J,K). Together, these data indicate that murine macrophages exposed to Nef-EVs both in vitro and in vivo exhibit increased mitochondrial respiration and aerobic glycolysis activity. To mimic the in vitro training model and assess the durability of these Nef-EV-induced metabolic adaptations, BMDMs were stimulated with Nef-EVs or GFP-EVs for 48 h, then washed and rested for an additional 5 days. Notably, the elevated mitochondrial mass, membrane potential, glucose uptake, and lactate production observed at 48 h post-Nef-EV treatment persisted in BMDM at day 7 (Fig. 3L–O). These findings confirm that Nef-EVs induce durable metabolic changes in macrophages, closely resembling the sustained metabolic reprogramming triggered by the prototypical training agent β-glucan (Ding et al, 2023; Saeed et al, 2014).

### Characterization of Nef-EV-induced inflammatory memory

**Figure 4:**
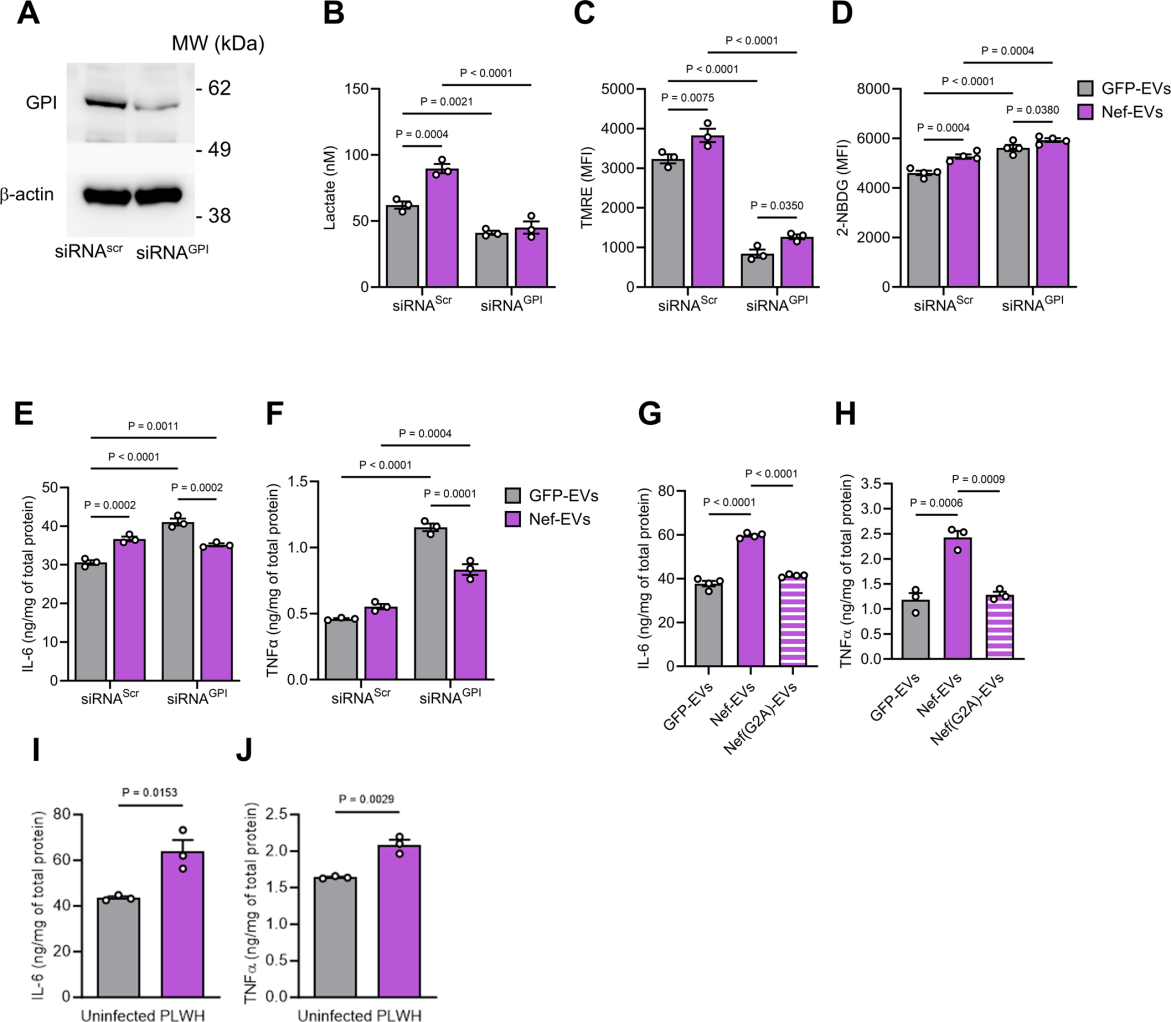
Characterization of Nef-EV-induced inflammatory memory. (**A**) Western blot of GPI (top) and β-actin (bottom) in BMDM 48 h post-transfection with siRNA for GPI or its scrambled control to confirm GPI knockdown. (**B**–**D**) BMDM were transfected with siRNA for GPI or scrambled control, then stimulated with Nef-EVs or GFP-EVs (0.4 × 10^9^ particles/ml, 48 h), and (**B**) lactate release, (**C**) TMRE, and (**D**) 2-NBDG were measured by flow cytometry. (**E**, **F**) BMDM were transfected with siRNA for GPI or scrambled control, then stimulated with Nef-EVs or GFP-EVs (0.4 × 10^9^ particles/ml, 48 h), then washed and rested for an additional 5 days prior to LPS stimulation (100 ng/ml, 4 h), and (**E**) IL-6 and (**F**) TNFα release were measured by ELISA. (**G**,** H**) BMDM were incubated with Nef-EVs, GFP-EVs, or Nef(2GA)-EVs (0.4 × 10^9^ particles/ml, 48 h), then washed and rested for an additional 5 days prior to LPS stimulation (100 ng/ml, 4 h), and IL-6 (**G**) and TNFα (**H**) release was measured by ELISA. (**I**, **J**) BMDM were incubated with EVs isolated from pooled plasma samples of 4 PLWH or 4 uninfected individuals, then washed and rested for an additional 5 days prior to LPS stimulation (100 ng/ml, 4 h), and IL-6 (**I**) and TNFα (**J**) release was measured by ELISA. Data are presented as mean ± SEM. (**B**–**F**: GFP-EVs, *n* = 3–4; Nef-EVs, *n* = 3–4; **G**, **H**: GFP-EVs, *n* = 4; Nef-EVs, *n* = 3–4; Nef(G2A)-EVs, *n* = 4). Data points represent biological replicates. Significance was determined by two-way ANOVA followed by a Bonferroni multiple comparisons test (**B**–**F**), one-way ANOVA (**G**), followed by a Tukey post hoc test (**G**, **H**), and a two-tailed unpaired Student’s *t* test (**I**, **J**) with calculated *P* values shown. Source data are available online for this figure.

To confirm the causal role of TRIM in the phenotype observed in Nef-EV-treated mice, we conducted complementary experiments in isolated BMDMs to assess the requirement for glycolysis, a pathway essential for TRIM induction. Because complete inhibition of glycolysis is lethal, we partially suppressed the pathway by silencing glucose-6-phosphate isomerase (GPI) with siRNA, a non-toxic approach (Wu et al, 2020). Transfection of BMDMs with GPI-targeting siRNA effectively reduced GPI abundance (Fig. 4A). When GPI-silenced BMDMs were exposed to Nef-EVs or GFP-EVs (0.4 × 10^9^ particles/ml) for 48 h, lactate production decreased by ~50% and mitochondrial membrane potential by ~80% (Fig. 4B,C). Consequently, the metabolic differences between Nef-EV- and GFP-EV-treated cells were largely eliminated. Glucose uptake was marginally increased in GPI-silenced BMDMs treated with both Nef-EVs and GFP-EVs (Fig. 4D). After a 5-day resting period without EVs, followed by LPS stimulation, control BMDMs with normal GPI levels exhibited the expected Nef-EV-driven increase in IL-6 and TNFα secretion compared to GFP-EV-treated cells (Fig. 4E,F). In contrast, Nef-EV-mediated cytokine increases were either abolished (for IL-6, Fig. 4E) or markedly reduced (for TNFα, Fig. 4F) in GPI-silenced cells. These findings demonstrate that active glycolysis is essential for the Nef-EV-driven TRIM phenotype and provide mechanistic support for the inflammatory memory paradigm observed in Nef-EV-treated mice.

Unexpectedly, GPI silencing elevated cytokine secretion in both GFP-EV- and Nef EV-treated BMDMs—a surprising finding given that glycolytic inhibition is typically considered anti-inflammatory and is associated with reduced cytokine production (Pajak et al, 2024). However, prior studies demonstrating this effect examined short-term (hours-long) glycolytic inhibition before LPS challenge, whereas our experiment involved sustained glycolytic suppression over 9 days prior to LPS treatment. Chronic alterations in energy metabolism can be functionally uncoupled from the acute inflammatory response to LPS (Ball et al, 2025). Moreover, genetic ablation of GPI in Th17 cells has been shown to potentiate, rather than suppress, inflammation (Wu et al, 2020).

To further characterize the formation of inflammatory memory, we used isolated BMDMs to compare the effects of Nef-EVs with EVs produced by Sup T1 cells expressing a myristoylation-deficient mutant of Nef, Nef(G2A). This Nef mutant lacks the N-terminal myristoylation signal, is excluded from EVs (Arenaccio et al, 2014b), and is inactive in modifying cholesterol metabolism (Mujawar et al, 2006). When tested in the in vitro training model, Nef(G2A)-EVs failed to induce durable inflammatory memory, producing no observable increase in LPS-induced IL-6 and TNFα release 5 days after EV treatment (Fig. 4G,H). These findings demonstrate that EV incorporation of Nef is necessary for the induction of trained immunity, directly implicating Nef as the active cargo responsible for reprogramming macrophage inflammatory responses.

Finally, we isolated EVs from pooled plasma samples of PLWH and uninfected individuals; these samples were previously characterized and tested for induction of acute inflammation in our earlier study (Mukhamedova et al, 2019). Using the same in vitro model of trained immunity, we found that IL-6 and TNFα production from BMDMs exposed to EVs from PLWH was significantly greater than from BMDMs exposed to EVs from uninfected individuals (Fig. 4I,J), demonstrating that circulating EVs from PLWH carry the capacity to induce trained immunity ex vivo.

### Prior exposure to Nef-EVs causes sustained elevation of myelopoiesis

The epigenetic and metabolic alterations observed in hematopoietic cells exposed to Nef-EVs suggest that these cells undergo long-term reprogramming of their inflammatory response program. To test this hypothesis formally, we performed a competitive BM transplantation experiment to determine whether Nef-EV-exposed hematopoietic progenitors retain a durably altered inflammatory phenotype in vivo, manifested as an enhanced response to a delayed secondary challenge, the defining functional criterion of trained immunity.

**Figure 5:**
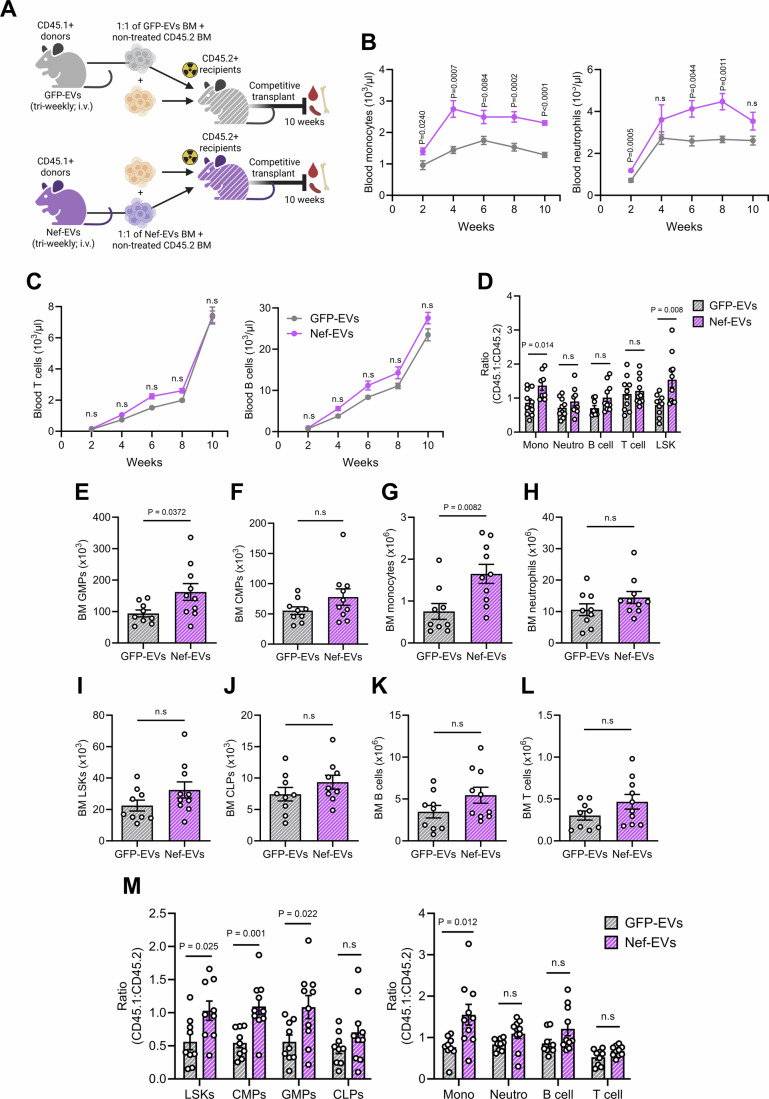
Monocytosis and myeloid progenitor expansion in recipient mice transplanted with bone marrow from Nef-EV-treated mice. (**A**) Schematic overview of competitive BM transplantation. Equally mixed portions of non-treated WT BM with either BM from Nef-EV- or GFP-EV-treated CD45.1 WT mice were transplanted into CD45.2 WT recipients. Engraftment and leukocytes from respective genotypes (CD45.1/CD45.2) were monitored over 10 weeks in blood, BM, and spleen. (**B**, **C**) Absolute number of (**B**) monocytes, neutrophils, and (**C**) T cells and B cells over 10 weeks in the blood of recipient mice (*n* = 9 mice/group). (GFP-EVs, *n* = 9; Nef-EVs, *n* = 9). (**D**) Ratio of CD45.1/CD45.2 in leukocytes and LSKs in the blood of recipient mice at week 10 (GFP-EVs, *n* = 9–10; Nef-EVs, *n* = 8–10). (**E**–**L**) Absolute number of (**E**) GMPs, (**F**) CMPs, (**G**) monocytes, (**H**) neutrophils, (**I**) LSKs, (**J**) CLPs, (**K**) B cells, and (**L**) T cells in the BM of recipient mice at week 10 (*n* = 9–10 mice/group) (GFP-EVs, *n* = 9; Nef-EVs, *n* = 10). (**M**) Ratio of CD45.1/CD45.2 in HSPC subsets and leukocytes in the BM of recipient mice at week 10 (GFP-EVs, *n* = 8–9; Nef-EVs, *n* = 9–10). Data are presented as mean ± SEM. Data points represent individual mice. Significance was determined by two-way ANOVA (mixed effects), analysis (**B**, **C**), multiple unpaired *t* tests (**D**, **M**), and a two-tailed unpaired Student’s *t* test (**E**–**L**) with calculated *P* values shown); n.s., not significant (*P* ≥ 0.05). Source data are available online for this figure.

To this end, mice carrying the CD45.1 allele were infused with Nef-EVs or GFP-EVs three times per week for 2 weeks (Fig. 5A). BM cells from these treated donor mice were isolated, mixed in equal numbers with BM cells from untreated mice carrying the CD45.2 allele, and transplanted into irradiated recipient mice also carrying the CD45.2 allele (see “Methods” for details). Recipient mice were maintained without EV treatment for 10 weeks, during which time blood samples were collected fortnightly. At the end of the experiment, BM, blood, and spleen were harvested and analyzed.

In recipient animals transplanted with BM from Nef-EV-treated donors, we observed a consistent increase in circulating monocyte abundance relative to recipients transplanted with BM from GFP-EV-treated donors, whereas neutrophil counts were elevated at early time points but returned toward baseline by week 10, suggesting that neutrophil progenitors retain Nef-EV-induced reprogramming less durably than monocyte progenitors (Fig. 5B). T- and B-cell abundances remained unchanged in both groups (Fig. 5C). To distinguish a cell-intrinsic effect of Nef-EV exposure from a cell-extrinsic effect driven by systemic factors such as elevated plasma cytokines, we calculated the ratio of cells carrying the CD45.1 allele (derived from BM progenitors exposed to either Nef-EVs or GFP-EVs) to those carrying the CD45.2 allele (derived from untreated BM progenitors; Fig. 5A). This ratio was significantly elevated for monocytes and LSKs in recipients of Nef-EV-exposed BM (Fig. 5D), demonstrating that the expanded monocyte output reflects a cell-intrinsic change in the exposed progenitors themselves rather than a response to the shared systemic environment of the recipient animal.

**Figure EV4:**
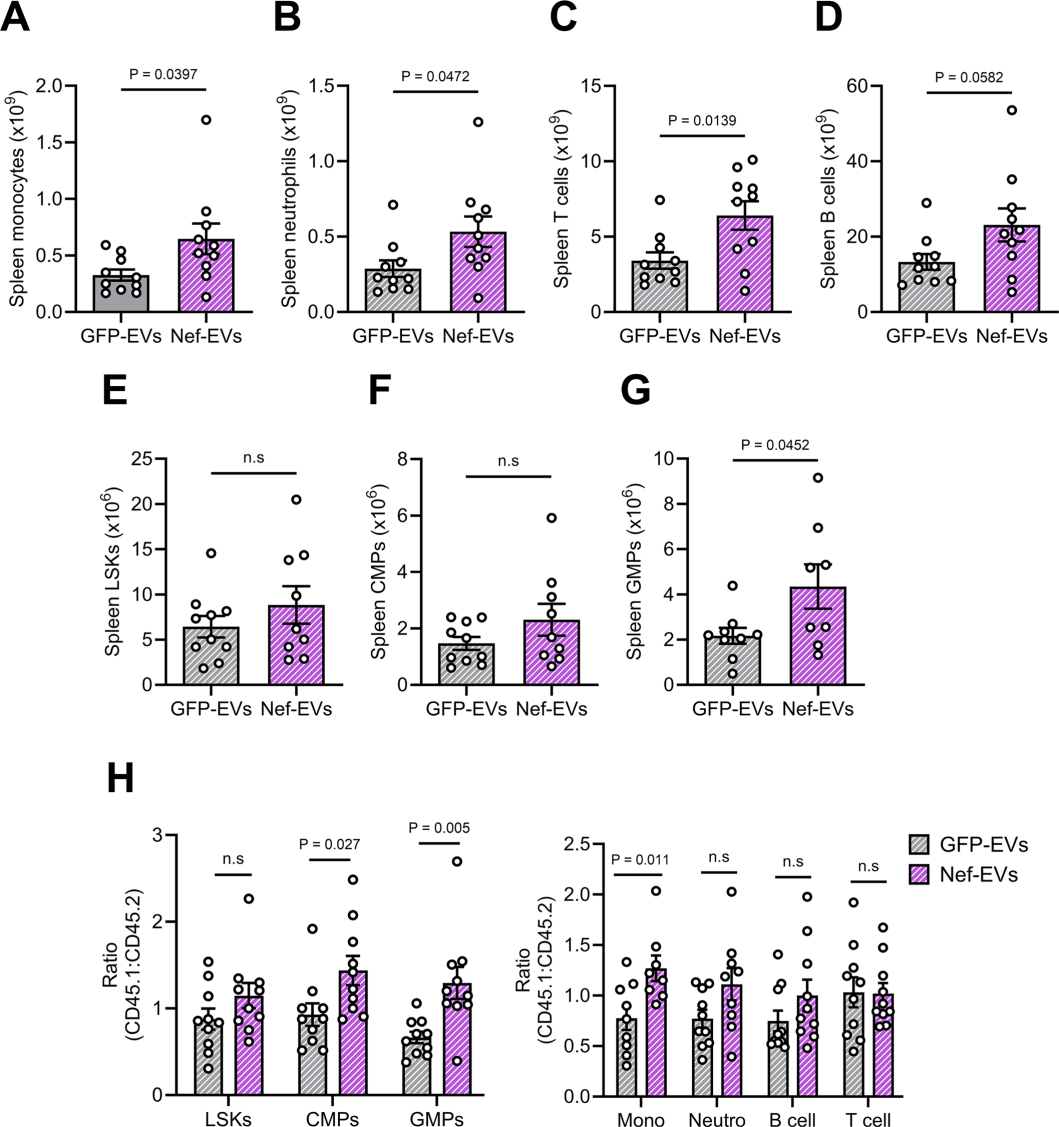
Monocytosis and myeloid progenitor expansion in the spleen of recipient mice transplanted with bone marrow from Nef-EV-treated mice. (**A**–**G**) Absolute number of monocytes (**A**), neutrophils (**B**), T cells (**C**), B cells (**D**), LSKs (**E**), CMPs (**F**), and GMPs (**G**) in the spleen of mice after GFP-EV or Nef-EV treatment (GFP-EVs, *n* = 10; Nef-EVs, *n* = 8–10). (**H**) Ratio of CD45.1/CD45.2 in LSKs and leukocytes in the spleen of recipient mice at week 10 (GFP-EVs, *n* = 10; Nef-EVs, *n* = 10). Data are presented as mean ± SEM. Data points represent individual mice. Significance was determined by two-tailed Student’s *t* test (**A**–**G**) or multiple unpaired *t* tests (**H**) with calculated *P* values shown; n.s., not significant (*P* ≥ 0.05). Source data are available online for this figure.

To determine whether the increased abundance of circulating myeloid cells reflected changes in the BM, we next examined the hematopoietic system of the recipient animals. Consistent with the observed myeloid bias in the blood, GMP and monocyte numbers were increased in mice transplanted with BM from Nef-EV-treated mice (Fig. 5E,G). There were no changes in the abundance of other major progenitor subsets (i.e., LSK, CMP, CLP; Fig. 5F,I,J) or mature leukocytes (i.e., neutrophils, B cells, T cells; Fig. 5H,K,L). Calculation of the CD45.1/CD45.2 ratio revealed a cell-intrinsic expansion of LSKs, CMPs, GMPs, and BM monocytes carrying the CD45.1 allele, i.e., cells originating from the BM of Nef-EV-exposed donor mice (Fig. 5M). Similarly, the numbers of monocytes, neutrophils, and GMPs were increased in the spleen of recipients transplanted with BM from Nef-EV-treated mice (Fig. EV4A–G). The cell-intrinsic expansion of CMP, GMP, and monocytes was also observed in the spleen (Fig. EV4H). Taken together, these data support the hypothesis that prior exposure to Nef-EVs promotes a legacy effect in the hematopoietic stem and progenitors for enhanced myeloid production across organ systems (BM, blood, and spleen).

**Figure 6:**
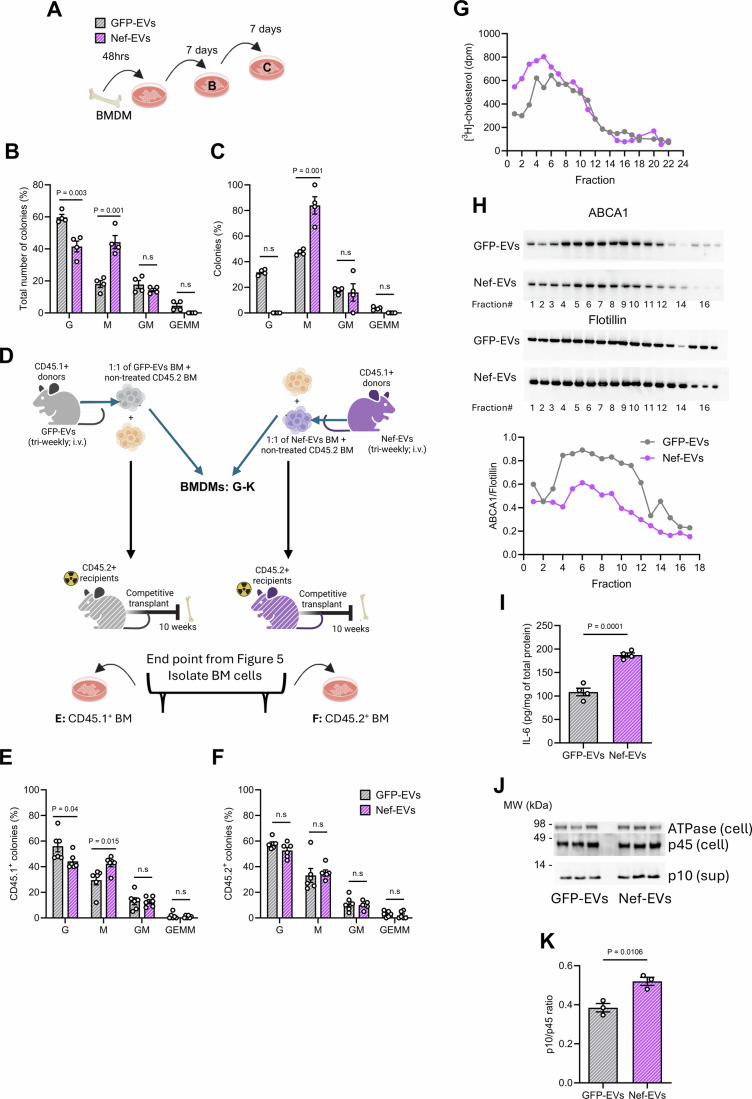
Bone marrow from mice treated with Nef-EVs is a durable source of macrophages with augmented inflammatory potential. (**A**–**C**) In vitro CFU assay: (**A**) overview, (**B**) Isolated murine stem cells treated with GFP-EVs and Nef-EVs for 48 h in vitro and plated in Mouse Methylcellulose Complete Media (MMCM). Colonies were visually identified and counted after 7 days. (**C**) Single colonies from (**B**) were re-plated to fresh dishes with MMCM, further grown for 7 days, and counted (GFP-EVs, *n* = 4; Nef-EVs, *n* = 4). G granulocytes, M macrophages, GM granulocytes/macrophages, GEMM granulocytes/erythrocytes/megakaryocytes/monocytes. (**D**–**F**) Outline of ex vivo analysis of BMT experiments. (**D**) Stem cells were isolated from the bone marrow of recipient mice at the end point of the experiment from Fig. 5A and separated into CD45.1^+^ and CD45.2^+^ cells by FACS. CFU assays were performed from (**E**) CD45.1+ (prior exposure to GFP-EVs or NEF-EVs) and (**F**) CD45.2+ (support marrow with no prior exposure). Colonies were visually identified and counted after 7 days. (GFP-EVs, *n* = 6; Nef-EVs, *n* = 6) Mean ± SD, *t* test, *n* = 4. G granulocytes, M macrophages, GM granulocytes/macrophages, GEMM granulocytes/erythrocytes/megakaryocytes /monocytes. (**G**) Abundance of lipid rafts. BMDM were derived from the bone marrow of recipient mice, cells were labeled with [^3^H]cholesterol, and lipid rafts were isolated as described in “Methods”. Profile of density gradient centrifugation is shown, where fractions 1–8 correspond to lipid rafts, a determination based on density, abundance of cholesterol, and of flotillin-1. (**H**) Abundance of ABCA1 in lipid rafts. Fractions from the experiment shown in (**E**) were analyzed by western blot for the abundance of ABCA1 and Flotillin. Densitometric analysis of the ABCA1 Flotillin ratio is shown below. (**I**) Secretion of IL-6 by macrophages derived from recipient mice. BMDM were stimulated with LPS (100 ng/ml for 4 h), media was collected over 4 h incubation. IL-6 concentration was determined by ELISA (GFP-EVs, *n* = 4; Nef-EVs, *n* = 4). (**J**, **K**) NLRP3 inflammasome activation in macrophages derived from recipient mice. BMDM were stimulated with LPS (100 ng/ml for 4 h), and western blot of p45 in cell lysate, p10 in cell culture media was analyzed (**J**). (**K**) Densitometric analysis of the western blot shown in (**G**), ratio of abundance of p10 to abundance of p45 is shown (*n* = 4). Data are presented as mean ± SEM. Data points represent biological replicates. Significance was determined by multiple unpaired *t* tests (**B**, **C**, **E**, **F**) or a two-tailed unpaired Student’s (**I**, **K**) with calculated *P* values shown); n.s., not significant (*P *≥ 0.05). Source data are available online for this figure.

To further confirm that the hematopoietic bias toward the myeloid lineage is maintained following transplantation of BM from Nef-EV-treated mice, we performed a colony-forming assay as outlined in Fig. 6A. When BM cells from untreated mice were treated with Nef-EVs in vitro for 48 h, we observed an increase in the number of macrophage colonies at the expense of granulocyte colonies (Fig. 6B). Moreover, upon replating, macrophage colonies derived from Nef-EV-treated BM continued to expand, whereas granulocyte colonies did not (Fig. 6C). We next applied the same assay to measure hematopoietic potential in recipient mice (as outlined in Fig. 6D), separating CD45.1+ and CD45.2+ cells by FACS. CD45.1^+^ carrying cells (derived from Nef-EV-exposed donor BM) showed preferential growth of macrophage colonies compared with GFP-EV-exposed controls (Fig. 6E). In contrast, no differences were observed in CD45.2^+^ cells (control, non-exposed BM from the respective recipients; Fig. 6F). These findings highlight the clear intrinsic Nef-EV-induced preference of monocyte production over granulocyte production and are consistent with the week 10 data from our competitive BMT experiment (Fig. 5B). Thus, increased myelopoiesis appears to be a long-term memory response of hematopoietic stem and progenitor cells transiently exposed to Nef-EVs.

### Prior exposure to Nef-EVs causes sustained metabolic changes in macrophages

To determine whether alterations in cholesterol metabolism persist as part of the trained immune ‘memory’, we next assessed whether the primary effects of Nef-EVs, namely increased lipid raft abundance and reduced ABCA1 levels, were retained following BM transplantation. BMDMs were isolated from the recipient mice described in Fig. 5A, and indeed, both elevated abundance of lipid raft and decreased ABCA1 abundance were observed in BMDMs derived from recipients transplanted with BM that had been exposed to Nef-EVs 11 weeks earlier (Fig. 6G,H). Furthermore, upon LPS stimulation, these macrophages secreted higher levels of IL-6 (Fig. 6I) and exhibited enhanced inflammasome activation, as indicated by an elevated ratio of cleaved caspase 1 p10 in the medium to full-length p45 in the cells (Fig. 6J,K). Thus, key mechanistic features of Nef-EV-induced innate memory, including cholesterol dysregulation, lipid raft enrichment, and heightened inflammasome activity, were stably maintained in cells derived from Nef-EV-exposed BM even 11 weeks after the initial exposure.

## Discussion

The findings of this study provide mechanistic insight into our initial observation that Nef-EVs can enhance inflammatory responses in macrophages following a delayed secondary stimulus, consistent with trained immunity (Dubrovsky et al, 2022). In the current study, we demonstrate that Nef-EVs can induce long-lasting inflammatory memory in macrophages and their bone marrow progenitors, establishing this as a previously unrecognized mechanism by which HIV drives chronic inflammation. We further demonstrate that Nef-EV-induced reprogramming is characterized by a pronounced and durable expansion of myeloid cells across organ systems, sustained through a cell-intrinsic change in hematopoietic progenitors as shown by competitive bone marrow transplantation. This reprogramming was accompanied by metabolic alterations, shifts in chromatin accessibility, and inflammatory gene expression profiles consistent with TRIM (Bekkering et al, 2018; Cheng et al, 2014; Ding et al, 2023; Netea et al, 2011). Notably, the enhanced inflammatory response was elicited by a secondary stimulus structurally unrelated to the primary inducer, fulfilling a key criterion that distinguishes trained immunity from classical priming and strengthening the case that Nef-driven TRIM contributes to the persistent innate immune activation characteristic of treated HIV infection.

The inflammatory memory caused by Nef-EVs emerged in BM myeloid progenitor cells, providing a long-lasting and renewable reservoir of mature myeloid cells with heightened inflammatory potential. TRIM-associated activation of progenitor cells was previously described by other groups for other TRIM inducers (Kain et al, 2023; Kaufmann et al, 2018; Mills et al, 2024; Mitroulis et al, 2018). We have previously reported a legacy effect in response to Nef in an in vitro setting (Dubrovsky et al, 2022), and other examples of TRIM formation in response to a single protein have also been documented (Sviridov et al, 2025). Unexpectedly, unlike other TRIM inducers in which lipid homeostasis alterations are transient and confined to the training period, the Nef-EV-induced changes in cholesterol metabolism, including reduced ABCA1 abundance and increased lipid raft formation, persisted in myeloid cells differentiated from hematopoietic progenitors 11 weeks after the initial EV exposure, suggesting that these metabolic changes are durably encoded in the progenitor population. Given that perturbed cholesterol efflux and increased lipid raft abundance have been linked to enhanced myelopoiesis (Murphy et al, 2011; Yvan-Charvet et al, 2010), we propose that these metabolic changes are mechanistically connected to the changes in cell-intrinsic myelopoiesis observed following Nef-EV exposure. The following findings support the role of Nef in the formation of TRIM: (*i*) Nef-EVs were taken up by BM cells; (*ii*) there was a notable similarity between the effect of Nef-EVs on BM cells in vivo and human and murine macrophages in vitro and ex vivo (Dubrovsky et al, 2022; Mukhamedova et al, 2019); (*iii*) the effects of Nef-EVs and rNef on hematopoiesis in vivo and inflammation in vivo and in vitro were qualitatively similar; (*iv*) EVs produced by cells expressing an inactive variant of Nef, Nef(G2A), did not cause formation of inflammatory memory; (*v*) proteomics analysis of Nef-EVs did not reveal another probable candidate for TRIM formation. Collectively, these findings make it likely that the effects of Nef result from direct interaction of Nef with BM cells, rather than mediated by systemic soluble factors. This is further supported by the transplantation experiments, where the TRIM phenotype was associated only with donor cells exposed to Nef-EVs. Notably, we have previously tested Nef proteins isolated from multiple HIV and SIV variants across a panel of assays underlying pro-inflammatory effects of Nef (Asztalos et al, 2010; Mujawar et al, 2006). In none of these assays did we observe qualitative differences in activity between Nef isolates, suggesting that the observed effects are a conserved property of Nef.

We also noted that the gene expression profile of Nef-EV-exposed BM monocytes (Fig. 2G) closely resembled that observed in BM monocytes from a pristane-induced model of classical trained immunity (Mills et al, 2024) and in monocytes/macrophages from PLWH (van der Heijden et al, 2021), all characterized by upregulation of genes linked to defense responses, myeloid cell activation, cytokine signaling and type I interferon responses. The convergence of these transcriptional signatures across mechanistically distinct contexts provides independent validation that Nef-EVs induce trained immunity through pathways shared with both canonical TRIM inducers and HIV infection itself.

TRIM exists in two forms, “adaptive”, which confers protection, and “maladaptive”, which causes inflammatory complications and co-morbidities (Netea and Joosten, 2024). The two forms can coexist; however, in HIV infection, as in many other chronic conditions, the maladaptive form predominates (Sviridov et al, 2025). We have previously suggested that the form of TRIM established after a primary challenge is influenced by events occurring in the interval between the primary and secondary challenges (Sviridov et al, 2025). If inflammation resolves during this period, adaptive TRIM is likely to develop. In contrast, if residual inflammation persists, it may potentiate TRIM-associated inflammatory responses, leading to the formation of maladaptive TRIM. We have previously demonstrated that Nef can cause chronic inflammation (Mukhamedova et al, 2019), and others have shown that, despite effective antiretroviral therapy, Nef is present in the blood of a large proportion of PLWH at concentrations causing both chronic inflammation and formation of TRIM in model systems (Dubrovsky et al, 2022; Raymond et al, 2011). Unlike β-glucan or BCG, which are administered as single bolus stimuli that mimic discrete infectious exposures, Nef-containing EVs are continuously produced and released into the circulation throughout the course of HIV infection, including during ART-suppressed disease. The repeated low-dose infusion protocol is therefore intended to model this chronic, low-level exposure rather than a single acute training event, and is more physiologically relevant to the clinical context we are studying. It is plausible that, combined with low-level endotoxemia due to gut barrier damage (Deeks et al, 2013), continuous exposure to Nef creates conditions favorable to the formation of maladaptive TRIM. TRIM, together with other drivers of inflammation, including the effects of Nef on cholesterol metabolism, may contribute to multiple HIV-associated co-morbidities. Moreover, while other HIV-related sources of inflammation would be expected to disappear if therapeutic efforts to “cure” HIV succeed, TRIM-imprinted progenitor cells may continue to elicit exaggerated responses to otherwise benign stimuli, thereby sustaining a degree of persistent inflammation. Although the magnitude and duration of such responses remain uncertain, the “cure” of HIV infection may not necessarily mark the end of its co-morbidities, which could instead persist as long-term sequelae.

### Limitations of the study

Most of the mechanistic studies described in this report were performed in a mouse model using EVs derived from human cells. While this approach enables rigorous analysis of Nef-specific effects, it is inherently limited in its ability to detect the contribution of other human proteins or RNAs incorporated into Nef-EVs, which may act additively or synergistically with Nef to modulate TRIM induction. Future studies in humanized mouse models will address this limitation. Although we demonstrate that Nef-EVs are sufficient to induce TRIM, other HIV proteins present in the blood of PLWH, such as Tat, may also contribute to, or even initiate, the formation of TRIM. The extent to which TRIM contributes to chronic inflammation in PLWH, as well as its persistence, remains unknown, and extrapolating from murine models is inherently challenging. The mechanistic studies presented here, while supporting the proposed pathway, remain largely associative, and the precise molecular mechanisms responsible for maintaining Nef-induced TRIM have yet to be defined. The causal contribution of glycolytic reprogramming to TRIM formation was examined in vitro, as in vivo testing presents fundamental interpretive obstacles. Global inhibition of glycolysis exerts broad effects on systemic metabolism and inflammation through mechanisms independent of trained immunity or the hematopoietic cell populations under study, confounding interpretation of any results obtained. Tissue-specific approaches, such as conditional knockout of GPI1 in myeloid progenitors analogous to the T cell-specific model described by Wu et al (Wu et al, 2020), face a different problem: because glycolysis is required for basal hematopoietic function, genetic disruption of essential glycolytic enzymes in progenitor cells would be expected to impair survival, proliferation, and engraftment, testing fundamental hematopoietic viability rather than the specific role of metabolic rewiring in immune memory establishment. Also, there is limited evidence for the stability and mechanisms of epigenetic adaptations in hematopoietic progenitors and their causal link to enhanced secondary responses.

### Conclusions

In conclusion, we found that a single HIV protein, Nef, can induce TRIM in murine hematopoietic stem and progenitor cells, ultimately leading to sustained myelopoiesis and the generation of inflammatory macrophages. If confirmed in humans, this phenomenon may contribute to persistent inflammation in PLWH and to the pathogenesis of HIV-associated inflammatory co-morbidities. Our findings also highlight potential challenges that may persist even after a functional cure of HIV.

## Methods

**Table:** Reagents and tools table

Reagent/resource	Reference or source	Identifier or catalog number
**Experimental models**		
Bone marrow-derived macrophages	mice C57BL/6J	N/A
SupT1 Human T Lymphoblasts	ATCC, Manassas, VA	Cat#CRL-1942
NCTC L929 Fibroblasts	ATCC, Manassas, VA	Cat#CCL-1
Mice/ C57BL/6J (M)	AMREP AS	In-house stock
CD45.2^+^ WT	AMREP AS	In-house stock
CD45.1^+/+^ (B6.SJL-Ptprc^a^Pepc^b^/BoyJ)	Ozgene	JAX stock 002014
**Recombinant DNA**		
HIV-1 Nef_SF2_WT	PMID: 7882168	Gift from Dr. M. Peterlin
pEGP-C1	Clontech	N/A
HIV-1 Nef-eGFP	PMID: 16928758	Gift from Dr. O. T. Fackler
HIV-1 Nef(G2A)	PMID: 16684552	Gift from Dr. O. T. Fackler
**Antibodies**		
Mouse anti-ABCA1 monoclonal antibody	Abcam	Cat#ab18180
Mouse potassium anti-sodium ATPase monoclonal antibody	Abcam	Cat#ab7671
Mouse anti-β -actin monoclonal antibody	Sigma-Aldrich	Cat#A1978
Rabbit anti-flotillin-1 monoclonal antibody	Cell Sign. Tech.	Cat#18634
Rabbit monoclonal anti-phospho-DRP1 antibody	Cell Sign. Tech	Cat#4494
Rabbit monoclonal anti-DRP1 antibody	Cell Sign. Tech	Cat#8570
Rabbit monoclonal anti-Caspase 1 antibody	Abcam	Cat#ab207802
GPI polyclonal antibody	Thermo Fisher Scientific	Cat#PA5-97517
Anti-mouse HRP-linked antibody	Cell Sign. Tech	Cat#7076
Anti-rabbit HRP-linked antibody	Cell Sign. Tech	Cat#7074
Rat monoclonal FITC anti-mouse/human CD45R/B220 Antibody	BioLegend	Cat#103206
Rat monoclonal FITC anti-mouse CD3 antibody	BioLegend	Cat#100204
Rat monoclonal antibody FITC anti-mouse TER-119	BioLegend	Cat#116206
Rat monoclonal Pacific Blue(TM) anti-mouse Ly-6A/E (Sca-1) antibody	BioLegend	Cat#122520
Rat monoclonal APC/Cyanine7 anti-mouse CD117 (c-kit) antibody	BioLegend	Cat#105826
Rat monoclonal PE anti-mouse CD135 antibody	BioLegend	Cat#135306
Rat monoclonal CD11b antibody (M1/70), FITC antibody	Invitrogen	Cat#MA1-10081
Hamster monoclonal anti-CD48 antibody	BD Biosciences	Cat#740236
Rat monoclonal Brilliant Violet 605(TM) anti-mouse CD150 (SLAM) antibody	BioLegend	Cat#115927
Rat monoclonal CD16/CD32 antibody	BD Biosciences	Cat#740217
Hamster monoclonal PE anti-mouse CD34 antibody	BioLegend	Cat#128610
Rat monoclonal BUV395 anti-mouse CD45 Clone 30-F11 (RUO) antibody	BD Biosciences	Cat#564279
Rat monoclonal PerCP-Cy5.5 Anti-Mouse CD45.1 (RUO), clone A20 antibody	BD Biosciences	Cat#560580
Rat monoclonal Ly-6G/Ly-6C Antibody (RB6-8C5) antibody	eBioscience	Cat#14-5931-82
Rat monoclonal PE/Cyanine7 anti-mouse CD115 (CSF-1R) antibody	BioLegend	Cat#135524
Rat monoclonal antibody Pacific Blue(TM) anti-mouse CD19	BioLegend	Cat#152416
Rat monoclonal antibody BV786 anti-mouse Ly-6C	BD Biosciences	Cat#569011
**Oligonucleotides and other sequence-based reagents**		
Silencer Select Negative Control No. 1	Thermo Fisher Scientific	Cat#4390843
Silencer Select GPI1 siRNA	Thermo Fisher Scientific	Cat#S66931
**Chemicals, enzymes, and other reagents**		
BD Horizon™ Fixable Viability Stain 510	BD Biosciences	Cat#564406
VeriBlot IP Detection reagent	Abcam	Cat#ab131366
Lipofectamine™ LTX Reagent with PLUS™ Reagent	Thermo Fisher Scientific	Cat#15338100
INTERFERin	Polyplus-Sartorius	Cat#101000036
RPMI 1640	Thermo Fisher Scientific	Cat#21870076
Opti-MEM	Thermo Fisher Scientific	Cat#32985070
IMDM	Thermo Fisher Scientific	Cat#31980030
FBS	Bovogen	Cat#SFBS-AU
Penicillin-Streptomycin	Thermo Fisher Scientific	Cat#15070063
Lipopolysaccharide (LPS)	Sigma-Aldrich	Cat#437627
Nigericin	Sigma-Aldrich	Cat#TA9H9877350F
Affinity Prosep-A High Capacity	Millipore	Cat#113111824
OptiPrep (Iodixanol) Density Gradient Medium	StemCell Tech	Cat#07820
Mouse Methylcellulose Complete Media	R&D Systems	Cat#HSC007
Tetramethylrhodamine, Ethyl Ester, Perchlorate (TMRE)	Thermo Fisher Scientific	Cat#T669
MitoTracker™ Green FM Dye	Thermo Fisher Scientific	Cat#M46750
2--NBDG (2-(N-(7-Nitrobenz-2-oxa-1,3-diazol-4-yl)Amino)-2-Deoxyglucose)	Thermo Fisher Scientific	Cat#N13195
Pierce Protease and Phosphatase Inhibitor Mini Tablets	Thermo Fisher Scientific	Cat#A32959
Cholesterol [1,2-3H(N)]	American Radiolabeled Chemicals	Cat#ART 0255
Acetic acid [^3^H] sodium salt	American Radiolabeled Chemicals	Cat#ART 0202
Mouse IL-6 Uncoated ELISA Kit	Thermo Fisher Scientific	Cat#88-7064-88
Mouse TNF alpha Uncoated ELISA Kit	Thermo Fisher Scientific	Cat#88-7324-88
EasySep FITC-positive selection kit	StemCell Tech	Cat#17682
Pierce™ Bradford Plus Protein Assay Reagent	Thermo Fisher Scientific	Cat#23238
Lactate Assay Kit	Sigma-Aldrich	Cat#MAK064
**Software**		
GraphPad Prism	http://www.graphpad.com/	RRID:SCR_002798
FlowJo	https://www.flowjo.com/solutions/flowjo	RRID:SCR_008520
**Other**		
Deposited data		
GFP,S1,snATACseq	GEO	GSM9539093
NEF,S1,snATACseq	GEO	GSM9539094
GFP,S1,snRNAseq	GEO	GSM9539095
NEF,S1,snRNAseq	GEO	GSM9539096
20231025_Eclipse-OT_DT_DS_2648_1_01	ProteomeXchange	PXD065713
20231025_Eclipse-OT_DT_DS_2648_2_01	ProteomeXchange	PXD065713
20231025_Eclipse-OT_DT_DS_2648_3_01	ProteomeXchange	PXD065713
20231025_Eclipse-OT_DT_DS_2648_4_01	ProteomeXchange	PXD065713

### Mice

All animal experiments were approved by the Alfred Medical Research Education Precinct (AMREP) animal ethics committee (P1761) and conducted in accordance with the Australian code of practice for the care and use of animals for scientific purposes, as stipulated by the National Health and Medical Research Council of Australia. Wild-type (C57BL/6J) and WT congenic CD45.1^+/+^ (B6.SJL-Ptprc^a^–Pep3^b^-/BoyJ) mice were purchased from Jackson Laboratories and colonies maintained by the AMREP animal facility. All mice were housed in a normal 12-hour light/dark cycle and had ad libitum access to water and food. Mice were fed a normal chow diet and were randomly assigned to treatment groups; endpoint analysis was blinded.

### Animal studies

C57BL/6J mice (8 male mice per group, 8 weeks old) were administered either Nef-EVs (10^9^ EVs, containing 1 ng of Nef), or the same number of control EVs (GFP-EVs), or 50 ng of recombinant Nef, three times a week, for a period of 2 weeks as described previously (Mukhamedova et al, 2019). At the end of the experiment, mice were euthanized, and blood, spleen, and BM were collected for downstream flow cytometry processing. Total WBC count was obtained from freshly collected blood, spleen, or BM diluted in cell pack buffer (1:7) using the Sysmex XN-550i automated hematology analyzer (capillary mode).

### Competitive BM transplantation study

In a separate experiment, irradiated recipient mice (CD45.2^+^ WT, *n* = 9/group) received BM transplantation with an equal mix (1:1 ratio) of BM from non-treated WT CD45.2^+^ mice alongside BM cells from donor (CD45.1^+^ WT) mice treated with either Nef-EVs or GFP-EVs, as detailed above. Recipient mice were administered a total of 5 × 10^6^ cells/mouse (i.v.). Over 10 weeks, the respective genotype of blood cells was monitored by flow cytometry (i.e., CD45.1/CD45.2). At week 10, the mice were sacrificed, and BM cells were analyzed for the contribution of each respective genotype via flow cytometry. Data were calculated as a CD45.2/CD45.1 (Dragoljevic et al, 2018).

### Flow cytometry

Monocytes, neutrophils, T cells, and B cells were identified from whole blood as previously described (Nagareddy et al, 2013). Blood was collected by cardiac puncture into EDTA-lined tubes and immediately placed on ice. All the following steps were performed on ice. RBCs were lysed, and WBCs were centrifuged, washed, and resuspended in FACS buffer (Hanks balanced salt solution + 0.1% BSA w/v, 5 mM EDTA). Cells were stained with a mixture of antibodies against CD45.2, CD45.1, Ly6-C/G (Gr-1), CD115, CD3, and B220. Monocytes were identified as CD45^+^CD115^hi^; neutrophils were identified as CD45^+^CD115^lo^Ly6-C/G^hi^ (Gr-1). T cells were identified as CD45^+^, CD115^−^, Gr1^−^, CD3^+^ and B220^−^. B cells were identified as CD45^+^, CD115^−^, Gr1^−^, CD3^−^ and B220^+^. All antibody staining was done at 1:400 dilution for 30 min in the dark on ice in FACS buffer.

Hematopoietic stem and progenitor cells from the BM were analyzed by flow cytometry as previously described (Murphy et al, 2011). BM was harvested from femurs and tibias by flushing with ice-cold PBS. RBCs were lysed, and WBCs were centrifuged, washed, and resuspended in FACS buffer. Single cell suspensions were stained with a cocktail of antibodies against lineage-committed cells (B220, CD19, CD11b, CD3e, TER-119,), Sca1, c-Kit, Flt3, CD16/CD32, and CD34. LSK (Lin-neg, Sca-1^+^ c-Kit^+^) and HSPC subsets were characterized as MEP (CD16/32^−^CD34^+^cKit^+^), CMP (CD16/32^int^CD34^int^cKit^+^), and GMP (CD16/32^+^CD34^+^cKit^+^). CLPs were characterized as Lin-neg, Sca-1^+^ c-Kit^+^ CD127^+^. All samples were stained with viability stain (BD Biosciences). All antibody staining was done at 1:400 dilution for 40 min in the dark on ice in FACS buffer. Staining was stopped with FACS buffer, and cells were subsequently washed and filtered through a 35-µm strainer before analysis. FACS was performed at the ARA Flow Cytometry Core Facility on a LSRII Fortessa (BD Biosciences). Data were analyzed using FlowJo software v.10.9 (BD).

### Mouse transcriptomic and genomic library preparation for snRNA and snATAC-sequencing

Briefly, frozen BM samples from Nef-EVs- and GFP-EVs-treated mice were gently thawed, pooled, and filtered (40 µm Flowmi Cell Strainer) before undergoing cell enrichment. To do this, BM cells were stained with FITC-labeled B220, CD19, CD2, CD3, CD4, CD8, and Ter119 antibodies to deplete samples of lymphocytes and RBCs using EasySep FITC-positive selection kit (StemCell Tech, Cat 17682). The enriched samples were then washed, counted, and stained with a cocktail of antibodies against lineage-committed cells (B220, CD2, CD3, CD4, CD8, Ter119, and CD19), CD45, cKit, Sca-1, CD11b, Ly6C, Ly6G, and viability dye BV510 at 4 °C for 45 min. Cells were washed, filtered, and then LSKs, cKit^+^, and monocytes were sorted using a BD FACS Melody. Viable FACS-sorted LSKs (Lin^neg^, cKit^+^Sca-1^+^), myeloid progenitors (Lin^neg^, cKit^+^), and monocytes (CD45^+^, CD11b^+^, Ly6C^+^) were then lysed and nuclei isolation performed as described by the 10X Genomics protocol (CG000365, Rev D). Briefly, cells were spun down at 400×*g*, for 5 min at 4 °C. The supernatant was carefully aspirated, and the pellet was resuspended in 100 µL of chilled *Lysis Buffer* (10X Genomics) for 4 min. After the incubation period, 1 mL of *Wash Buffer* was added, and the nuclei were pelleted at 1000×*g*, 5 min at 4 °C, and resuspended in *Wash Buffer*. Two additional washes were performed. At this step, the nuclei were resuspended in diluted nuclei buffer and counted. Nuclei from LSKs, cKit^+^ cells, and monocytes were combined to prepare a single nuclei suspension per experimental group containing ~50% LSKs, 30% cKit^+^ myeloid progenitors, and 20% myeloid cells. A final nuclei count was performed, and the nuclei were filtered (40-µm Flowmi Cell Strainer) prior to loading for partitioning. Transcriptomic (snRNA) and genomic (snATAC) libraries were prepared by loading 11,500 nuclei per lane and following *Chromium Next GEM Single Cell Multiome ATAC + Gene Expression Kits User Guide* with all required proprietary kits and equipment including the 10X Genomics Chromium Controller (1000204, 10X Genomics), Chromium Next GEM Single Cell Multiome ATAC + Gene Expression Kit (PN- 1000283, 10x Genomics), Chromium Next GEM Chip J Single Cell Kit (PN-1000230, 10X Genomics) and Dual Index Kit TT Set A (PN-1000215, 10X Genomics). Libraries were pooled evenly, and paired-end sequencing on an Illumina NovaSeq 6000 was performed.

### Sequencing, pre-processing, QC, filtering, and processing for mouse snRNA and snATAC libraries

10x Multiome ATAC FASTQ and GEX FASTQ files were downloaded from Illumina and processed using Cell Ranger ARC v2.0.2 (10X Genomics). Reads were aligned to the pre-built mouse reference genome mm10-2020-A-2.0.0 to quantify gene expression and chromatin accessibility for each cell. ATAC and GEX matrices were constructed, and barcodes with high-quality signals in both modalities were identified as cells using the joint cell-calling algorithm. Analyses of pre-processed snRNA-seq and snATAC-seq data were performed in R v4.2.2 using Seurat v5.0.0 and v5.0.3 (satijalab.org/seurat) (Hao et al, 2024) and Signac v1.12.0 (stuartlab.org/signac) (Stuart et al, 2021) R packages. RNA and ATAC matrices for each sample were pre-processed independently. A separate Seurat object was created for each sample based on the gene expression data, with the ATAC data added as a second assay. Uniform Maximum Approximation and Projection (UMAP) was used to visualize the data in two dimensions. Top marker genes for each cell cluster were identified using the joinLayers and FindAllMarkers functions. Cell clusters were annotated based on their unique marker gene expression profiles in the RNA-seq data. The final dataset comprised 7734 nuclei, 32,285 genes, and 141,700 ATAC fragments across two samples—4322 nuclei from the GFP-EVs sample and 3412 nuclei from the Nef-EVs sample.

### Differential expression, gene ontology, and eRegulon analysis

Differential expression (DE) analysis was performed between GFP and NEF samples for each cell population separately. Genes with nonzero expression in >10% of cells in at least one of the experimental groups were included in the analysis. The MAST R package v1.24.1 (Finak et al, 2015) was used to perform DE testing method “MASTcpmDetRate” considering the cellular detection rate as a covariate. A threshold of uncorrected *P* <  0.01 was used to define statistically significant DE genes between groups. Gene Ontology (GO) over-representation analysis for differentially expressed gene lists (uncorrected *P* < 0.01) was performed using the enrichGO function from the clusterProfiler R package (Yu et al, 2012). The org.Mm.eg.db: Genome-wide annotation for Mouse, R package (version 3.16.0) was used to obtain all gene ontology mappings for Mus Musculus. Over-representation of GO Biological Process (GO-BP) terms was calculated using the entire list of genes detected in the experiment as the background gene list, with minimum and maximum gene set sizes of 10 and 500, respectively. The similarity between enriched GO-BP terms was calculated using the simplify R function from the clusterProfiler R package. The Benjamini–Hochberg adjusted *P* value cutoff of 0.05 was used to determine statistically significant GO-BP terms. For eRegulon analysis, was performed using SCENIC+ version 1.0a2, a Python package for predicting enhancer gene regulatory networks (eGRNs) by integrating snRNA and snATAC data (Bravo Gonzalez-Blas et al, 2023). For preprocessing of the snATAC, pycisTopic (version 2.0a0) was employed to initialize a cisTopic object and perform Latent Dirichlet Allocation (LDA) modeling on the peak-cell matrix extracted from the integrated Seurat object. A model comprising 30 regulatory topics was selected, and the results were used to infer candidate enhancer regions. To construct a custom cisTarget motif database, peaks were called from ATAC fragments generated by 10X Cell Ranger ARC using MACS3 with default parameters. Consensus peak sets were derived after filtering against the mm10-blacklist.v2.bed file obtained from the SCENIC+ database. A custom motif database was subsequently generated using these consensus regions, applying the *–bgpadding* = *1000* parameter to define background regions. SCENIC+ was executed using Snakemake, integrating the reprocessed snRNA data from the Seurat object, the cisTopic model, and the custom cisTarget motif database.

### Cells

Murine bone marrow-derived macrophages (BMDM) were isolated from tibia and femur bones of 6–8 week-old C57BL/6 male mice as described by Shrestha et al (Shrestha et al, 2016). In brief, bone marrow was flushed out from bones with IMDM containing 5% FBS. Cells were spun, pellet washed, and incubated for 10 min in red blood cell lysis buffer. Cells were washed twice with cold PBS. After the last spin pellet was resuspended in IMDM containing 10% FBS and 15% of L929 cell-conditioned media. Cells were plated in Petri dishes (BD) and cultured for 5–7 days. For in vitro experiments, cells were incubated for 48 h with Nef-EVs or GFP-EVs at a final concentration of 0.4 × 10^9^ particles/ml (~0.4 ng/ml Nef for Nef-EVs). Human lymphoid cell line SupT1(ATCC, Manassas, VA) was grown in RPMI-1640 medium supplemented with 10% fetal bovine serum (FBS) (Bovogen). Murine L929 fibroblasts (ATCC, Manassas, VA) were cultured in serum-free IMDM for 10 days. Media was collected and used to supplement BMDM growth media.

### Transfection

SupT1 cells were transfected either with HIV-1 Nef_SF2_WT (kind gift of Dr. Matija Peterlin), HIV-Nef(G2A) (kind gift of Dr. Oliver Fackler), or control pEGFP- C1 plasmid (Clontech) using Lipofectamine LTX with Plus Reagent (Thermo Fisher Scientific) according to the manufacturer’s protocol with minor modifications. Average transfection efficiency was 70%,

BMDM were transfected either with siRNA_neg.control_ (Thermo Fisher Scientific) or siRNA_Gpi1_ (Thermo Fisher Scientific) using INTERFERin (Polyplus-Sartorius) according to the manufacturer’s protocol. Briefly, Gpi1 or negative control siRNA were diluted in Opti-MEM® Reduced Serum Medium. INTERFERin was added and immediately vortexed. The mixture was incubated for 15 min at room temperature. Mixture of the siRNA-INTERFERin complexes was added dropwise to the cells and plates were spun at 200×*g* for 5 min. Cells were incubated for 5 h, and incubation media was replaced with fresh complete media. Silencing efficiency was estimated in 48 h by western blot.

### EVs isolation and purification

Transfected SupT1 cells were grown in RPMI supplemented with 10% heat-inactivated exosome-depleted FBS. EVs were isolated as described (Rider et al, 2016). Forty-eight hours post transfection EV-containing medium was pre-cleared from cellular debris by centrifugation (2000×*g*) for 30 min and cleared by passing through 0.45-μm syringe filters. The samples were mixed in equal volume of 16% Polyethylene glycol 6000 in 1 M NaCl (Sigma-Aldrich), incubated at 4 °C for 18 h on a rotating platform, and centrifuged at 4 °C, 4000×*g* for 90 min. Pellets were collected, resuspended in PBS, and centrifuged again for 2 h at 4 °C, 100,000×*g*. Total protein content in EV samples was estimated by Bradford assay after dilution in RIPA buffer and boiling for 3 min. The purity, size distribution, and concentration of the EVs were analyzed using Nanoparticle Tracking Analysis with NanoSight NS300 (ATA Scientific Instruments). Monoclonal anti-Nef antibodies were used to detect the presence of Nef in the Nef-EVs by Western blotting. Nef concentration in EVs was standardized to a known recombinant Nef (rNef) concentration. Final pellet was resuspended in PBS, aliquoted, and frozen at −80 °C

EVs were also isolated from pooled plasma samples from four subjects with PLWH and four uninfected subjects. These pooled plasma samples were obtained, used, and described in our previous study (Mukhamedova et al, 2019) and were kept at −80 °C. Original human ethics approvals for the study where these samples were collected permitted the extension of analysis of the collected samples. EVs were isolated as described above except that the final concentration of polyethylene glycol was reduced to 5% and the incubation time to 2 h to avoid co-precipitation of plasma lipoproteins.

### Proteomics of EVs

Samples were homogenized in a lysis buffer (5% SDS, 50 mM TEAB, pH 8.5), sonicated, and cleared by centrifugation (10,000×*g*, 10 min). Proteins were precipitated with the addition of ice-cold acetone to the supernatant (4:1) at −20 °C, precipitated proteins were pelleted by centrifugation and then subjected to column S-trap digestion (ProtiFi, NY, USA) according to the manufacturer’s workflow. Prior to analysis, peptides were reconstituted in loading buffer (3% ACN, 0.1% FA), and samples were diluted as required and loaded onto the analyzer through LC-MS.

Peptides analyzed by LC-MS using an UltiMateTM 3000 HPLC System (Thermo Fisher Scientific, MA, USA) equipped with an in-house C18 resin (1.9 μM, 50 cm) column for reversed phase separation that was coupled to an Orbitrap Eclipse mass spectrometer (Thermo Scientific). Peptides were separated by increasing the concentration of the mobile phase over a 70 min gradient from 95% Buffer A (0.1% FA), 5% Buffer B (80% ACN, 0.1% FA) to 95% Buffer B, 5% Buffer A at a flow rate of 300 nL min^−1^. Peptide identification was carried out using a data-dependent acquisition method. The MS1 scan range was set to 350–1400 *m/z* with AGC target of 100% and dynamic exclusion set to 15 s. The MS2 scan range was set to automatic with a AGC target of 200% and an isolation window of 1.4 *m/z*, HCD NCE was set to 30%.

LC-MS/MS data were processed and analyzed in Proteome Discoverer 2.5 (Thermo Fisher Scientific, MA, USA) and peptide spectral matches (PSMs) were searched using MASCOT against a Human database in FASTA format with the following parameters: precursor mass tolerance, 10 ppm; fragment mass tolerance, 0.8 Da; maximum missed trypsin cleavage sites, 2; dynamic modifications; cysteine (Cys) carbamidomethyl, protein N-terminal actyl, methionine (Met) oxidation.

### Lipid raft isolation

Lipid raft isolation was done as described previously (Mukhamedova et al, 2020). In brief, cells were collected, and the membrane fraction was prepared by hypo-osmotic shock. After 400×*g* spin, the supernatant was taken and spun at 350,000×*g* for 90 min. Pellet was resuspended and homogenized, and protein content was estimated. Then, the sample was spun in two consecutive Iodixanol linear gradient runs (10–20% and 5–15%) at 52,000×*g* for 90 min each. After the second spin, the top part of the sample was collected by 20 µl aliquots. Aliquots were either counted on a β-counter or processed for western blot for semi-quantitative analysis of raft marker, flotillin-1.

### Inflammasome activation

Murine BMDM isolated from recipient mice were seeded in 10% FBS IMDM supplemented with 15% L929 conditioned media. To prime inflammasomes, cells were incubated with LPS (100 ng/ml) for 4 h. Then, the cells were incubated with Nigericin (5 μM) for 3 h. Cell lysates were collected for the estimation of procaspase-1 (p45) by Western blot. Cell culture supernatants were collected to measure the abundance of p10 fragment of caspase 1 by IP. Supernatants were mixed with anti-Caspase 1 antibodies and incubated overnight at 4 °C. ProsepA High Affinity beads were added and incubated for a further 6 h at 4 °C. Beads were washed with ice-cold PBS and boiled 3 min in loading buffer containing 5% 2-mercaptoethanol. Samples were analyzed by Western blotting. To avoid interference from denatured IgG VeriBlot detection reagent (HRP) was used.

### Western blot

Cells were lysed with RIPA buffer, protein concentration in lysates estimated by Bradford assay, followed by SDS-PAGE and transfer of proteins to PVDF membrane. Semi-quantitative analysis of western blots was performed by densitometry and presented as a proportion of control after normalization to loading controls.

### ELISA for cytokines

TNFα and IL-6 in the supernatants of bone marrow-derived macrophages were measured by commercial ELISA assays (Thermo Fisher Scientific) according to the manufacturer’s protocol. Briefly, for cellular experiments, BMDM were stimulated for 4 h with 100 ng/ml LPS, supernatants were collected and used in ELISA at several dilutions. Cells were lysed and protein concentration measured by Bradford assay (Thermo Fisher Scientific). Final cytokine concentration was normalized to cellular protein content.

### Colony-forming unit assay (CFU)

CFU was performed in mouse methylcellulose complete media (R&D Systems) according to the manufacturer's protocol. In brief, murine bone marrow cells were isolated as described above, resuspended in IMDM/2% FBS, and counted. Cells were mixed with Mouse Methylcellulose Complete Media and vortexed to mix the cells thoroughly with the media. The final cell mixture was loaded into a 35-mm culture dish using a 3 mL syringe fitted with an 18-gauge needle. The final cell number was 5 × 10^4^ per dish. The cells were incubated for 6–10 days at 37 °C and 5% CO_2_, and the colonies were visually identified and manually counted.

### Metabolic and mitochondrial measurements

BMDMs were treated with Nef-EVs or control GFP-EVs for 48 h followed by washing and a 5-day rest period in the growing media, before being prepared for the following metabolic and mitochondrial analyses. BMDM were incubated for 30 min at 37 °C in FACS buffer with mitochondrial dyes: TMRE (25 nM, Thermo Fisher Scientific) or MitoTracker Green (25 nM, Thermo Fisher Scientific) according to the manufacturer’s instructions. Cells were washed twice (400×*g*, 5 min) and analyzed by flow cytometry. For measurement of glucose uptake, BMDM were cultured for the final 2 h of the 48 h stimulation period in 100 μL of glucose-free medium containing 200 μM of 2-(n-(7-nitrobenz-2-oxa-1,3-diazol-4-ylamino)-2-deoxyglucose (2-NBDG) at 37 °C. After 2 h, cells were washed and processed for quantification of 2-NBDG uptake (MFI) by flow cytometry. Lactate concentration in the culture supernatant was measured by colorimetric assay (Sigma-Aldrich) and read at 570 nM on a BioRad 680 Plate Reader.

### Cholesterol biosynthesis

Cholesterol biosynthesis was assessed as described previously (Fu et al, 2004). Briefly, BMDM from mice treated either with GFP-EVs or Nef-EVs (4 each) were isolated as described above, seeded in IMDM containing 10% FBS, and incubated for 96 h. Then cells were washed and incubated for 2 h at 37 °C in serum-free IMDM containing 0.1%BSA and [^3^H]acetate (final radioactivity of 7.4 MBq/ml). Cells were washed twice and collected in distilled water. Lipid extraction and TLC using cholesterol and CE system were done as described previously (Sviridov and Fidge, 1995).

## Supplementary information

Peer Review File

Dataset EV1

Source data Fig. 1

Source data Fig. 3

Source data Fig. 4

Source data Fig. 5

Source data Fig. 6

Figure EV1 Source Data

Figure EV2 Source Data

Figure EV3 Source Data

Figure EV4 Source Data

Expanded View Figures

## Data Availability

The RNA-Seq and ATAC datasets generated and/or analyzed during the current study are accessible at NCBI GEO (accession number GSE320286). The mass spectrometry proteomics data have been deposited to the ProteomeXchange Consortium via the PRIDE partner repository with the dataset identifier PXD065713. The source data of this paper are collected in the following database record: biostudies:S-SCDT-10_1038-S44319-026-00838-w.

## References

[CR1] Adzhubei AA, Kulkarni A, Tolstova AP, Anashkina AA, Sviridov D, Makarov AA, Bukrinsky MI (2021) Direct interaction between ABCA1 and HIV-1 Nef: molecular modeling and virtual screening for inhibitors. Comput Struct Biotechnol J 19:3876–3884 34584633 10.1016/j.csbj.2021.06.050 PMC8440812

[CR2] Aiello A, Giannessi F, Percario ZA, Fecchi K, Arenaccio C, Leone S, Carollo M, D’Aversa E, Chaperot L, Gambari R et al (2021) HIV-1 Nef protein affects cytokine and extracellular vesicles production in the GEN2.2 plasmacytoid dendritic cell line. Viruses 14:74 35062278 10.3390/v14010074 PMC8780779

[CR3] Arenaccio C, Chiozzini C, Columba-Cabezas S, Manfredi F, Affabris E, Baur A, Federico M (2014a) Exosomes from human immunodeficiency virus type 1 (HIV-1)-infected cells license quiescent CD4+ T lymphocytes to replicate HIV-1 through a Nef- and ADAM17-dependent mechanism. J Virol 88:11529–11539 25056899 10.1128/JVI.01712-14 PMC4178784

[CR4] Arenaccio C, Chiozzini C, Columba-Cabezas S, Manfredi F, Federico M (2014b) Cell activation and HIV-1 replication in unstimulated CD4+ T lymphocytes ingesting exosomes from cells expressing defective HIV-1. Retrovirology 11:46 24924541 10.1186/1742-4690-11-46 PMC4229896

[CR5] Asztalos BF, Mujawar Z, Morrow MP, Grant A, Pushkarsky T, Wanke C, Shannon R, Geyer M, Kirchhoff F, Sviridov D et al (2010) Circulating Nef induces dyslipidemia in simian immunodeficiency virus-infected macaques by suppressing cholesterol efflux. J Infect Dis 202:614–623 20617930 10.1086/654817 PMC2932757

[CR6] Ball AB, Jones AE, Nguyễn KB, Rios A, Marx N, Hsieh WY, Yang K, Desousa BR, Kim KKO, Veliova M et al (2025) Pro-inflammatory macrophage activation does not require inhibition of oxidative phosphorylation. EMBO Rep 26:982–1002 39753784 10.1038/s44319-024-00351-y PMC11850891

[CR7] Bekkering S, Arts RJW, Novakovic B, Kourtzelis I, van der Heijden C, Li Y, Popa CD, Ter Horst R, van Tuijl J, Netea-Maier RT et al (2018) Metabolic induction of trained immunity through the mevalonate pathway. Cell 172:135–146.e139 29328908 10.1016/j.cell.2017.11.025

[CR8] Bravo Gonzalez-Blas C, De Winter S, Hulselmans G, Hecker N, Matetovici I, Christiaens V, Poovathingal S, Wouters J, Aibar S, Aerts S (2023) SCENIC+: single-cell multiomic inference of enhancers and gene regulatory networks. Nat Methods 20:1355–1367 37443338 10.1038/s41592-023-01938-4 PMC10482700

[CR9] Cheng SC, Quintin J, Cramer RA, Shepardson KM, Saeed S, Kumar V, Giamarellos-Bourboulis EJ, Martens JH, Rao NA, Aghajanirefah A et al (2014) mTOR- and HIF-1α-mediated aerobic glycolysis as metabolic basis for trained immunity. Science 345:1250684 25258083 10.1126/science.1250684 PMC4226238

[CR10] Cheong JG, Ravishankar A, Sharma S, Parkhurst CN, Grassmann SA, Wingert CK, Laurent P, Ma S, Paddock L, Miranda IC et al (2023) Epigenetic memory of coronavirus infection in innate immune cells and their progenitors. Cell 186:3882–3902.e3824 37597510 10.1016/j.cell.2023.07.019 PMC10638861

[CR11] Chou TC, Maggirwar NS, Marsden MD (2024) HIV persistence, latency, and cure approaches: where are we now? Viruses 16:1163 39066325 10.3390/v16071163 PMC11281696

[CR12] Cui HL, Grant A, Mukhamedova N, Pushkarsky T, Jennelle L, Dubrovsky L, Gaus K, Fitzgerald ML, Sviridov D, Bukrinsky M (2012) HIV-1 Nef mobilizes lipid rafts in macrophages through a pathway that competes with ABCA1-dependent cholesterol efflux. J Lipid Res 53:696–708 22262807 10.1194/jlr.M023119 PMC3307646

[CR13] da Silva-Januario ME, da Costa CS, Tavares LA, Oliveira AK, Januario YC, de Carvalho AN, Cassiano MHA, Rodrigues RL, Miller ME, Palameta S et al (2023) HIV-1 Nef changes the proteome of T cells extracellular vesicles depleting IFITMs and other antiviral factors. Mol Cell Proteom 22:100676 10.1016/j.mcpro.2023.100676 PMC10746527 37940003

[CR14] Deeks SG, Tracy R, Douek DC (2013) Systemic effects of inflammation on health during chronic HIV infection. Immunity 39:633–645 24138880 10.1016/j.immuni.2013.10.001 PMC4012895

[CR15] Ding C, Shrestha R, Zhu X, Geller AE, Wu S, Woeste MR, Li W, Wang H, Yuan F, Xu R et al (2023) Inducing trained immunity in pro-metastatic macrophages to control tumor metastasis. Nat Immunol 24:239–254 36604547 10.1038/s41590-022-01388-8 PMC10636755

[CR16] Dragoljevic D, Kraakman MJ, Nagareddy PR, Ngo D, Shihata W, Kammoun HL, Whillas A, Lee MKS, Al-Sharea A, Pernes G et al (2018) Defective cholesterol metabolism in haematopoietic stem cells promotes monocyte-driven atherosclerosis in rheumatoid arthritis. Eur Heart J 39:2158–2167 29905812 10.1093/eurheartj/ehy119 PMC6001889

[CR17] Dubrovsky L, Brichacek B, Prashant NM, Pushkarsky T, Mukhamedova N, Fleetwood AJ, Xu Y, Dragoljevic D, Fitzgerald M, Horvath A et al (2022) Extracellular vesicles carrying HIV-1 Nef induce long-term hyperreactivity of myeloid cells. Cell Rep 41:111674 36417867 10.1016/j.celrep.2022.111674 PMC9733434

[CR18] Dubrovsky L, Ward A, Choi S-H, Pushkarsky T, Brichacek B, Vanpouille C, Adzhubei AA, Mukhamedova N, Sviridov D, Margolis L et al (2020) Inhibition of HIV replication by apolipoprotein A-I binding protein targeting the lipid rafts. mBio 11:e02956–02919 31964734 10.1128/mBio.02956-19 PMC6974568

[CR19] Fanucchi S, Domínguez-Andrés J, Joosten LAB, Netea MG, Mhlanga MM (2021) The intersection of epigenetics and metabolism in trained immunity. Immunity 54:32–43 33220235 10.1016/j.immuni.2020.10.011

[CR20] Ferdin J, Goricar K, Dolzan V, Plemenitas A, Martin JN, Peterlin BM, Deeks SG, Lenassi M (2018) Viral protein Nef is detected in plasma of half of HIV-infected adults with undetectable plasma HIV RNA. PLoS ONE 13:e0191613 29364927 10.1371/journal.pone.0191613 PMC5783402

[CR21] Finak G, McDavid A, Yajima M, Deng J, Gersuk V, Shalek AK, Slichter CK, Miller HW, McElrath MJ, Prlic M et al (2015) MAST: a flexible statistical framework for assessing transcriptional changes and characterizing heterogeneity in single-cell RNA sequencing data. Genome Biol 16:278 26653891 10.1186/s13059-015-0844-5 PMC4676162

[CR22] Fitzgerald ML, Mujawar Z, Tamehiro N (2010) ABC transporters, atherosclerosis and inflammation. Atherosclerosis 211:361–370 20138281 10.1016/j.atherosclerosis.2010.01.011 PMC2888932

[CR23] Fu Y, Hoang A, Escher G, Parton RG, Krozowski Z, Sviridov D (2004) Expression of Caveolin-1 enhances cholesterol efflux in hepatic cells. J Biol Chem 279:14140–14146 14729661 10.1074/jbc.M311061200

[CR24] Gu J, Liu Q, Zhang J, Xu S (2023) COVID-19 and trained immunity: the inflammatory burden of long COVID. Front Immunol 14:1294959 38090572 10.3389/fimmu.2023.1294959 PMC10713746

[CR25] Hajishengallis G, Netea MG, Chavakis T (2023) Innate immune memory, trained immunity and nomenclature clarification. Nat Immunol 24:1393–1394 37620602 10.1038/s41590-023-01595-x

[CR26] Hao Y, Stuart T, Kowalski MH, Choudhary S, Hoffman P, Hartman A, Srivastava A, Molla G, Madad S, Fernandez-Granda C et al (2024) Dictionary learning for integrative, multimodal and scalable single-cell analysis. Nat Biotechnol 42:293–304 37231261 10.1038/s41587-023-01767-y PMC10928517

[CR27] Hunegnaw R, Vassylyeva M, Dubrovsky L, Pushkarsky T, Sviridov D, Anashkina AA, Üren A, Brichacek B, Vassylyev DG, Adzhubei AA et al (2016) Interaction between HIV-1 Nef and Calnexin: from modeling to small molecule inhibitors reversing HIV-induced lipid accumulation. Arterioscler Thromb Vasc Biol 36:1758–1771 27470515 10.1161/ATVBAHA.116.307997 PMC5040351

[CR28] Kain BN, Tran BT, Luna PN, Cao R, Le DT, Florez MA, Maneix L, Toups JD, Morales-Mantilla DE, Koh S et al (2023) Hematopoietic stem and progenitor cells confer cross-protective trained immunity in mouse models. iScience 26:107596 37664586 10.1016/j.isci.2023.107596 PMC10470378

[CR29] Kaufmann E, Sanz J, Dunn JL, Khan N, Mendonça LE, Pacis A, Tzelepis F, Pernet E, Dumaine A, Grenier JC et al (2018) BCG educates hematopoietic stem cells to generate protective innate immunity against tuberculosis. Cell 172:176–190.e119 29328912 10.1016/j.cell.2017.12.031

[CR30] Kfoury YS, Ji F, Mazzola M, Sykes DB, Scherer AK, Anselmo A, Akiyama Y, Mercier F, Severe N, Kokkaliaris KD et al (2021) tiRNA signaling via stress-regulated vesicle transfer in the hematopoietic niche. Cell Stem Cell 28:2090–2103.e2099 34551362 10.1016/j.stem.2021.08.014 PMC8642285

[CR31] Khan MB, Lang MJ, Huang MB, Raymond A, Bond VC, Shiramizu B, Powell MD (2016) Nef exosomes isolated from the plasma of individuals with HIV-associated dementia (HAD) can induce Aβ(1-42) secretion in SH-SY5Y neural cells. J Neurovirol 22:179–190 26407718 10.1007/s13365-015-0383-6 PMC4783240

[CR32] Kumar CS, Muthukumaran G, Frost LJ, Noe M, Ahn YH, Mariano TM, Pestka S (1989) Molecular characterization of the murine interferon gamma receptor cDNA. J Biol Chem 264:17939–17946 2530216

[CR33] Lavrin T, Loboda J, Ferdin J, Levak V, Sitar S, Holcar M, Resnik N, Stenovec M, Trampuš Bakija A, Veranič P et al (2025) HIV protein Nef expression in human microglia drives the release of distinct Nef-containing extracellular vesicles. Extracell Vesicles Circ Nucl Acids 6:895–920 41551605 10.20517/evcna.2025.106 PMC12809690

[CR34] Lenassi M, Cagney G, Liao M, Vaupotic T, Bartholomeeusen K, Cheng Y, Krogan NJ, Plemenitas A, Peterlin BM (2010) HIV Nef is secreted in exosomes and triggers apoptosis in bystander CD4+ T cells. Traffic 11:110–122 19912576 10.1111/j.1600-0854.2009.01006.x PMC2796297

[CR35] Li X, Wang H, Yu X, Saha G, Kalafati L, Ioannidis C, Mitroulis I, Netea MG, Chavakis T, Hajishengallis G (2022) Maladaptive innate immune training of myelopoiesis links inflammatory comorbidities. Cell 185:1709–1727.e1718 35483374 10.1016/j.cell.2022.03.043 PMC9106933

[CR36] McNamara RP, Costantini LM, Myers TA, Schouest B, Maness NJ, Griffith JD, Damania BA, MacLean AG, Dittmer DP (2018) Nef secretion into extracellular vesicles or exosomes is conserved across human and simian immunodeficiency viruses. mBio 9:e02344–17 29437924 10.1128/mBio.02344-17 PMC5801467

[CR37] Mills TS, Kain B, Burchill MA, Danis E, Lucas ED, Culp-Hill R, Cowan CM, Schleicher WE, Patel SB, Tran BT et al (2024) A distinct metabolic and epigenetic state drives trained immunity in HSC-derived macrophages from autoimmune mice. Cell Stem Cell 31:1630–1649.e1638 39413777 10.1016/j.stem.2024.09.010 PMC11560650

[CR38] Mitroulis I, Ruppova K, Wang B, Chen LS, Grzybek M, Grinenko T, Eugster A, Troullinaki M, Palladini A, Kourtzelis I et al (2018) Modulation of myelopoiesis progenitors is an integral component of trained immunity. Cell 172:147–161.e112 29328910 10.1016/j.cell.2017.11.034 PMC5766828

[CR39] Moorlag SJCFM, Folkman L, ter Horst R, Krausgruber T, Barreca D, Schuster LC, Fife V, Matzaraki V, Li W, Reichl S et al (2024) Multi-omics analysis of innate and adaptive responses to BCG vaccination reveals epigenetic cell states that predict trained immunity. Immunity 57:171–187.e114 38198850 10.1016/j.immuni.2023.12.005

[CR40] Mujawar Z, Rose H, Morrow MP, Pushkarsky T, Dubrovsky L, Mukhamedova N, Fu Y, Dart A, Orenstein JM, Bobryshev YV et al (2006) Human immunodeficiency virus impairs reverse cholesterol transport from macrophages. PLoS Biol 4:e365 17076584 10.1371/journal.pbio.0040365 PMC1629034

[CR41] Mukhamedova N, Hoang A, Dragoljevic D, Dubrovsky L, Pushkarsky T, Low H, Ditiatkovski M, Fu Y, Ohkawa R, Meikle PJ et al (2019) Exosomes containing HIV protein Nef reorganize lipid rafts potentiating inflammatory response in bystander cells. PLoS Pathog 15:e1007907 31344124 10.1371/journal.ppat.1007907 PMC6657916

[CR42] Mukhamedova N, Huynh K, Low H, Meikle PJ, Sviridov D (2020) Isolation of lipid rafts from cultured mammalian cells and their lipidomics analysis. Bio-Protoc 10:e3670 33659340 10.21769/BioProtoc.3670 PMC7842516

[CR43] Murphy AJ, Akhtari M, Tolani S, Pagler T, Bijl N, Kuo C-L, Wang M, Sanson M, Abramowicz S, Welch C et al (2011) ApoE regulates hematopoietic stem cell proliferation, monocytosis, and monocyte accumulation in atherosclerotic lesions in mice. J Clin Invest 121:4138–4149 21968112 10.1172/JCI57559 PMC3195472

[CR44] Nagareddy PR, Murphy AJ, Stirzaker RA, Hu Y, Yu S, Miller RG, Ramkhelawon B, Distel E, Westerterp M, Huang L-S et al (2013) Hyperglycemia promotes myelopoiesis and impairs the resolution of atherosclerosis. Cell Metab 17:695–708 23663738 10.1016/j.cmet.2013.04.001 PMC3992275

[CR45] Netea MG, Joosten LAB (2024) Trained immunity in the bone marrow: Hub of autoimmunity. Cell Stem Cell 31:1555–1557 39515297 10.1016/j.stem.2024.10.008

[CR46] Netea MG, Quintin J, van der Meer Jos WM (2011) Trained immunity: a memory for innate host defense. Cell Host Microbe 9:355–361 21575907 10.1016/j.chom.2011.04.006

[CR47] Noz MP, Bekkering S, Groh L, Nielen TM, Lamfers EJ, Schlitzer A, El Messaoudi S, van Royen N, Huys EH, Preijers FW et al (2020) Reprogramming of bone marrow myeloid progenitor cells in patients with severe coronary artery disease. eLife 9:e60939 33168134 10.7554/eLife.60939 PMC7665893

[CR48] Pajak B, Zielinski R, Priebe W (2024) The impact of glycolysis and its inhibitors on the immune response to inflammation and autoimmunity. Molecules 29:1298 38542934 10.3390/molecules29061298 PMC10975218

[CR49] Perkins MV, Joseph SB, Dittmer DP, Mackman N (2023) Cardiovascular disease and thrombosis in HIV infection. Arterioscler Thromb Vasc Biol 43:175–191 36453273 10.1161/ATVBAHA.122.318232 PMC10165851

[CR50] Puzar Dominkus P, Ferdin J, Plemenitas A, Peterlin BM, Lenassi M (2017) Nef is secreted in exosomes from Nef.GFP-expressing and HIV-1-infected human astrocytes. J Neurovirol 23:713–724 28762184 10.1007/s13365-017-0552-x PMC6010353

[CR51] Quintin J, Saeed S, Martens JHA, Giamarellos-Bourboulis EJ, Ifrim DC, Logie C, Jacobs L, Jansen T, Kullberg BJ, Wijmenga C et al (2012) *Candida albicans* infection affords protection against reinfection via functional reprogramming of monocytes. Cell Host Microbe 12:223–232 22901542 10.1016/j.chom.2012.06.006 PMC3864037

[CR52] Ramezani A, Dubrovsky L, Pushkarsky T, Sviridov D, Karandish S, Raj DS, Fitzgerald ML, Bukrinsky M (2015) Stimulation of liver X receptor has potent anti-HIV effects in a humanized mouse model of HIV infection. J Pharm Exp Ther 354:376–383 10.1124/jpet.115.224485 PMC4538872 26126533

[CR53] Raymond AD, Campbell-Sims TC, Khan M, Lang M, Huang MB, Bond VC, Powell MD (2011) HIV Type 1 Nef is released from infected cells in CD45(+) microvesicles and is present in the plasma of HIV-infected individuals. AIDS Res Hum Retroviruses 27:167–178 20964480 10.1089/aid.2009.0170 PMC3064529

[CR54] Rider MA, Hurwitz SN, Meckes DG Jr (2016) ExtraPEG: a polyethylene glycol-based method for enrichment of extracellular vesicles. Sci Rep 6:23978 27068479 10.1038/srep23978 PMC4828635

[CR55] Saeed S, Quintin J, Kerstens HH, Rao NA, Aghajanirefah A, Matarese F, Cheng SC, Ratter J, Berentsen K, van der Ent MA et al (2014) Epigenetic programming of monocyte-to-macrophage differentiation and trained innate immunity. Science 345:1251086 25258085 10.1126/science.1251086 PMC4242194

[CR56] Shrestha E, Hussein MA, Savas JN, Ouimet M, Barrett TJ, Leone S, Yates JR, Moore KJ, Fisher EA, Garabedian MJ (2016) Poly(ADP-ribose) polymerase 1 represses liver X receptor-mediated ABCA1 expression and cholesterol efflux in macrophages. J Biol Chem 291:11172–11184 27026705 10.1074/jbc.M116.726729 PMC4900266

[CR57] Stuart T, Srivastava A, Madad S, Lareau CA, Satija R (2021) Single-cell chromatin state analysis with Signac. Nat Methods 18:1333–1341 34725479 10.1038/s41592-021-01282-5 PMC9255697

[CR58] Sviridov D, Bukrinsky M (2023) Neuro-HIV—new insights into pathogenesis and emerging therapeutic targets. FASEB J 37:e23301 37942865 10.1096/fj.202301239RR PMC11032165

[CR59] Sviridov D, Fidge N (1995) Efflux of intracellular vs plasma membrane cholesterol in HepG2 cells: different availability and regulation by apolipoprotein A-I. J Lipid Res 36:1887–1896 8558077

[CR60] Sviridov D, Mukhamedova N, Makarov AA, Adzhubei A, Bukrinsky M (2020) Comorbidities of HIV infection: role of Nef-induced impairment of cholesterol metabolism and lipid raft functionality. AIDS 34:1–13 31789888 10.1097/QAD.0000000000002385 PMC6903377

[CR61] Sviridov D, Netea MG, Bukrinsky MI (2025) Maladaptive trained immunity in viral infections. J Clin Invest 135:e192469 40892509 10.1172/JCI192469 PMC12404746

[CR62] Taks EJM, Moorlag S, Netea MG, van der Meer JWM (2022) Shifting the immune memory paradigm: trained immunity in viral infections. Annu Rev Virol 9:469–489 35676081 10.1146/annurev-virology-091919-072546

[CR63] Triant VA, Perez J, Regan S, Massaro JM, Meigs JB, Grinspoon SK, D’Agostino RB (2018) Cardiovascular risk prediction functions underestimate risk in HIV infection. Circulation 137:2203–2214 29444987 10.1161/CIRCULATIONAHA.117.028975 PMC6157923

[CR64] van der Heijden WA, Van de Wijer L, Keramati F, Trypsteen W, Rutsaert S, Horst RT, Jaeger M, Koenen HJ, Stunnenberg HG, Joosten I et al (2021) Chronic HIV infection induces transcriptional and functional reprogramming of innate immune cells. JCI insight 6:e145928 33630761 10.1172/jci.insight.145928 PMC8119206

[CR65] van ‘t Wout AB, Swain JV, Schindler M, Rao U, Pathmajeyan MS, Mullins JI, Kirchhoff F (2005) Nef induces multiple genes involved in cholesterol synthesis and uptake in human immunodeficiency virus type 1-infected T cells. J Virol 79:10053–10058 16014965 10.1128/JVI.79.15.10053-10058.2005 PMC1181597

[CR66] Vanpouille C, Brichacek B, Pushkarsky T, Dubrovsky L, Fitzgerald W, Mukhamedova N, Garcia-Hernandez S, Matthies D, Popratiloff A, Sviridov D et al (2024) HIV-1 Nef is carried on the surface of extracellular vesicles. J Extracell Vesicles 13:e12478 39016173 10.1002/jev2.12478 PMC11252832

[CR67] Varshney P, Yadav V, Saini N (2016) Lipid rafts in immune signalling: current progress and future perspective. Immunology 149:13–24 27153983 10.1111/imm.12617 PMC4981613

[CR68] Welsh JA, Goberdhan DCI, O’Driscoll L, Buzas EI, Blenkiron C, Bussolati B, Cai H, Di Vizio D, Driedonks TAP, Erdbrugger U et al (2024) Minimal information for studies of extracellular vesicles (MISEV2023): From basic to advanced approaches. J Extracell Vesicles 13:e12404 38326288 10.1002/jev2.12404 PMC10850029

[CR69] Wu L, Hollinshead KER, Hao Y, Au C, Kroehling L, Ng C, Lin WY, Li D, Silva HM, Shin J et al (2020) Niche-selective inhibition of pathogenic Th17 cells by targeting metabolic redundancy. Cell 182:641–654 e620 32615085 10.1016/j.cell.2020.06.014 PMC7556360

[CR70] Yu G, Wang L-G, Han Y, He Q-Y (2012) clusterProfiler: an R package for comparing biological themes among gene clusters. OMICS: A J Integr Biol 16:284–287 10.1089/omi.2011.0118 PMC3339379 22455463

[CR71] Yvan-Charvet L, Pagler T, Gautier EL, Avagyan S, Siry RL, Han S, Welch CL, Wang N, Randolph GJ, Snoeck HW et al (2010) ATP-binding cassette transporters and HDL suppress hematopoietic stem cell proliferation. Science 328:1689–1693 20488992 10.1126/science.1189731 PMC3032591

[CR72] Zheng YH, Plemenitas A, Fielding CJ, Peterlin BM (2003) Nef increases the synthesis of and transports cholesterol to lipid rafts and HIV-1 progeny virions. Proc Natl Acad Sci USA 100:8460–8465 12824470 10.1073/pnas.1437453100 PMC166251

