# Supplementary figures and images for: HIV-1 Nef generates lasting innate immune memory in haematopoietic stem and progenitor cells in vivo
Source: EMBO Rep. 2026 Jun 15;27(14):4166–92. doi: 10.1038/s44319-026-00838-w (PMC13400611; doi:10.1038/s44319-026-00838-w)

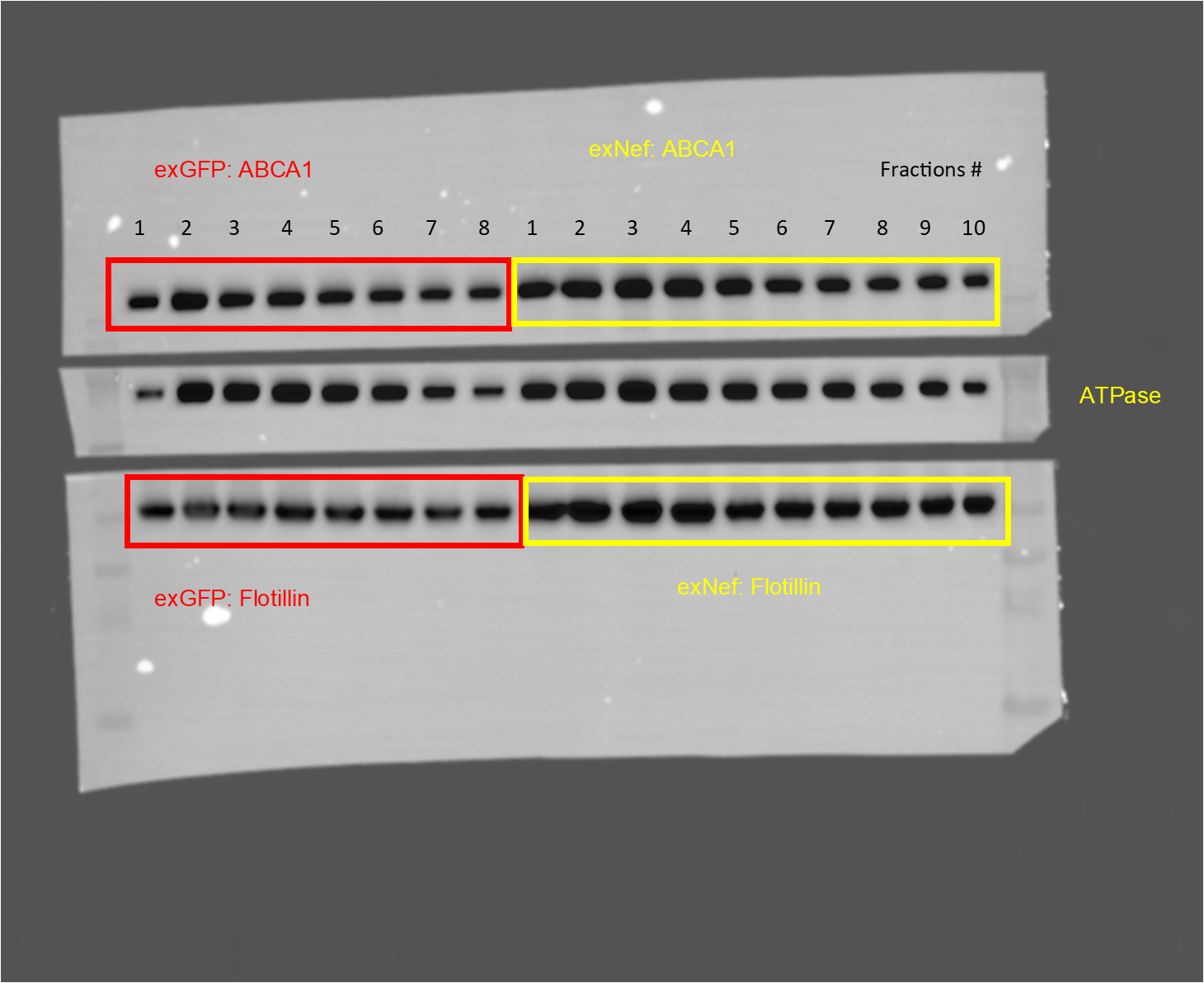

Supplement: Supplementary file 4 — Source data Fig. 3 [file 44319_2026_838_MOESM4_ESM.zip › Figure 3/Figure 3A/Figure 3A.tif]

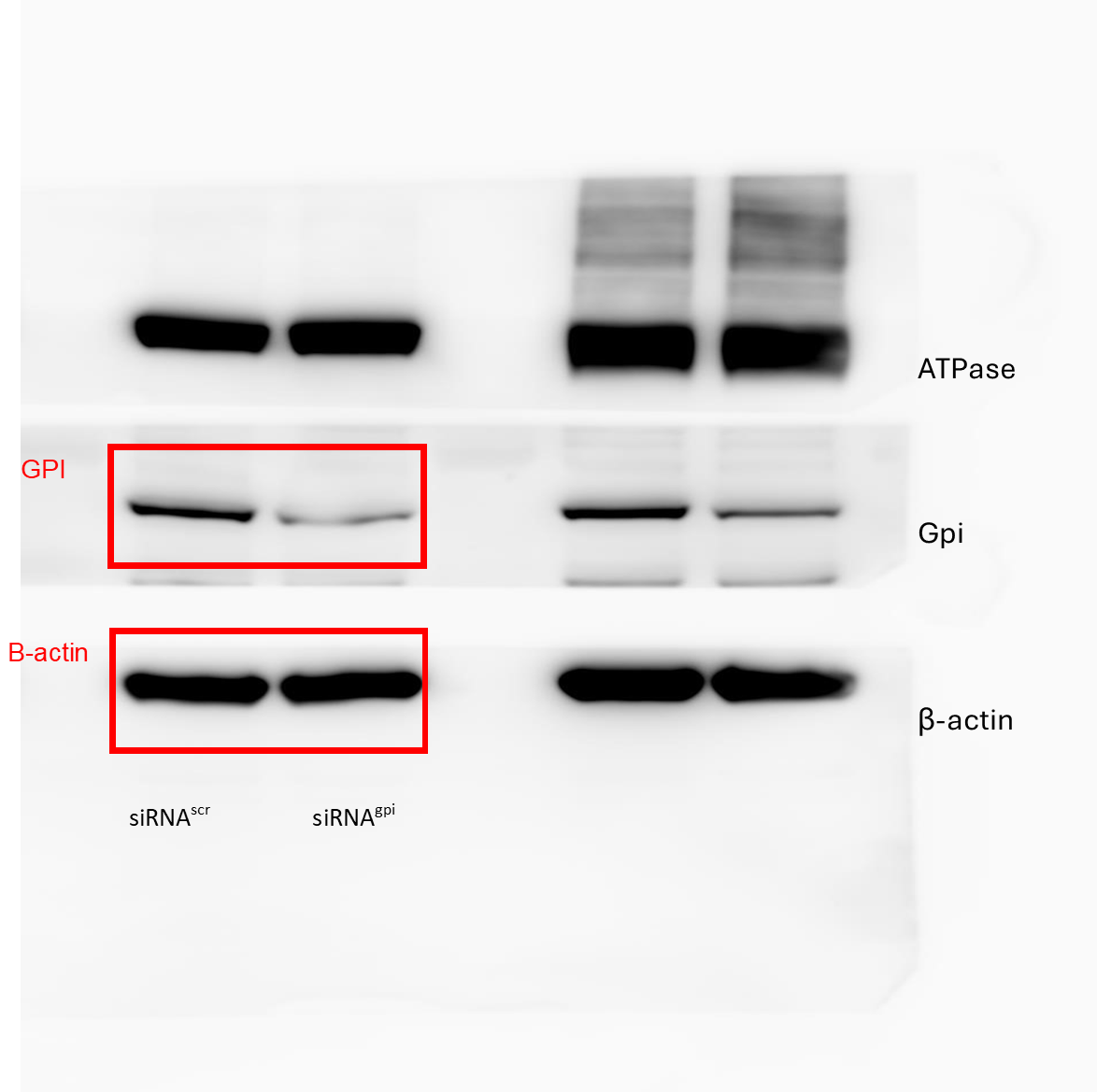

Supplement: Supplementary file 5 — Source data Fig. 4 [file 44319_2026_838_MOESM5_ESM.zip › Figure 4/Figure 4A/Figure 4A.tif]

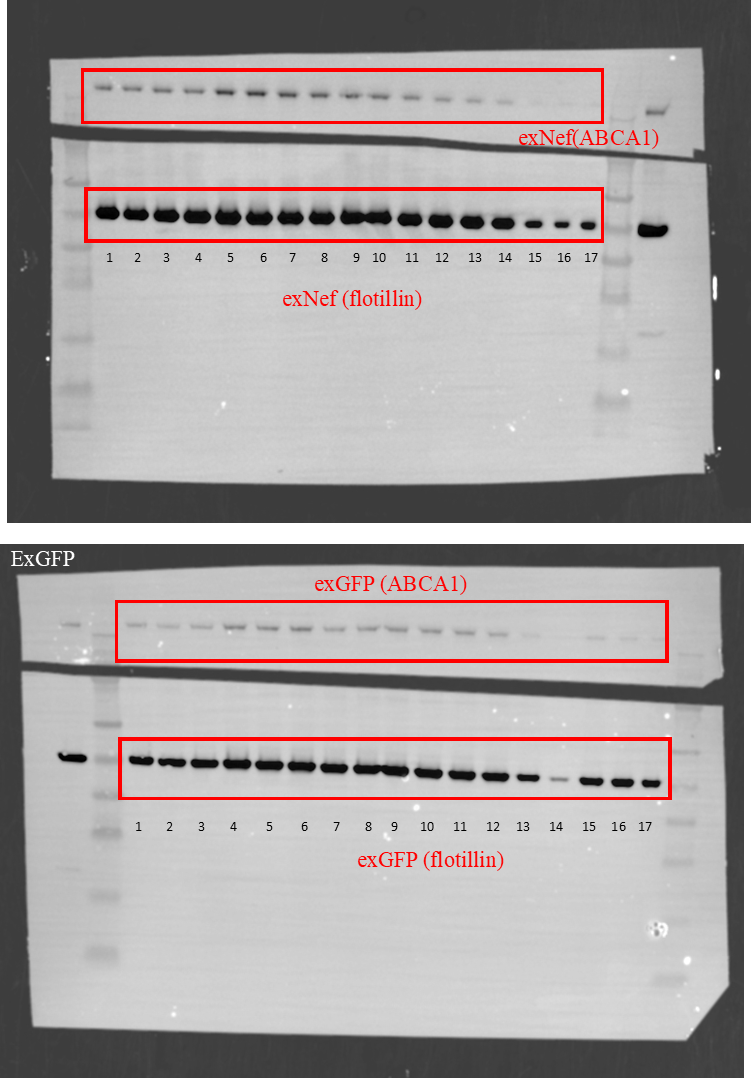

Supplement: Supplementary file 7 — Source data Fig. 6 [file 44319_2026_838_MOESM7_ESM.zip › Figure 6/Figure 6H Western blot/Fig 6H Western blot.tif]

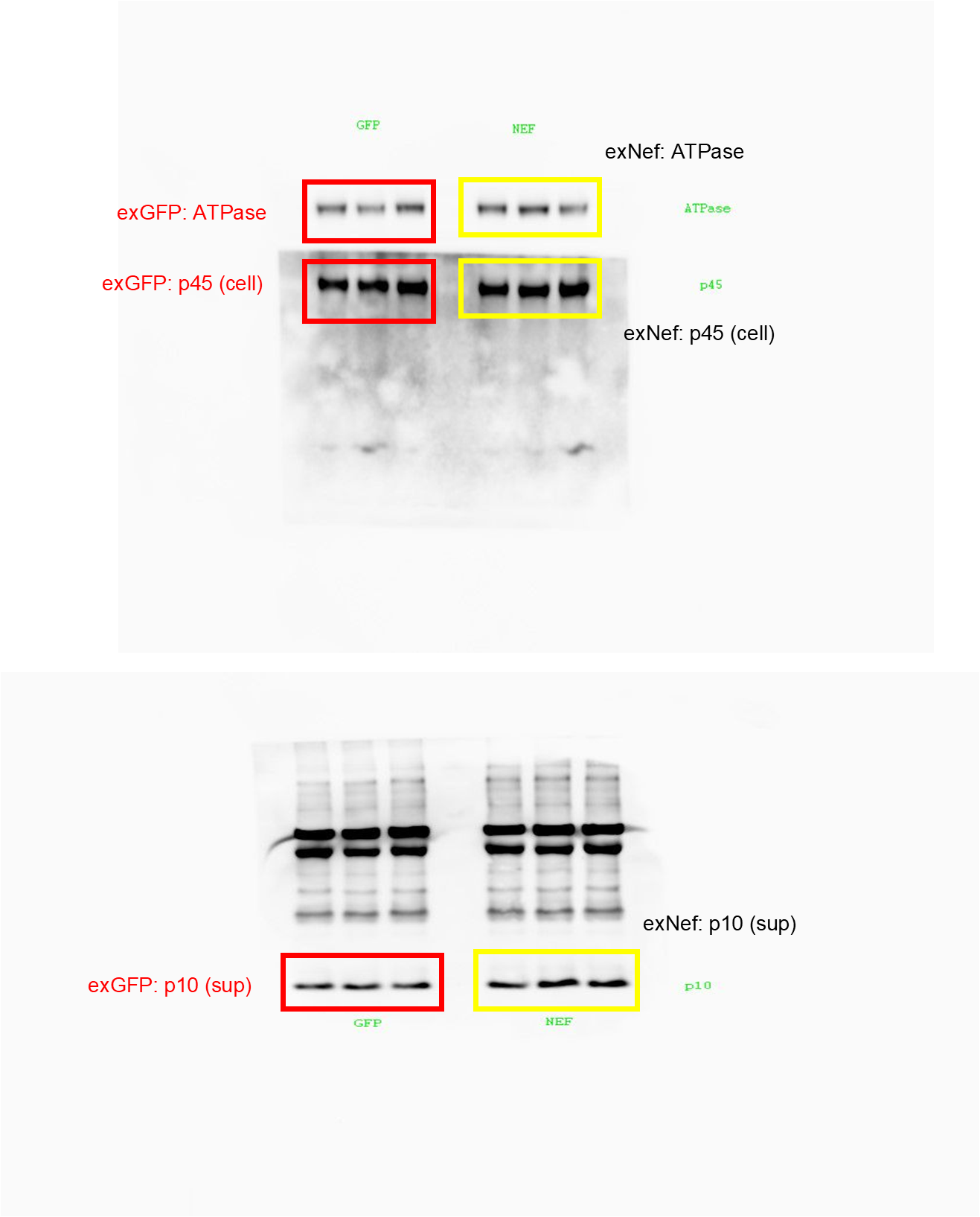

Supplement: Supplementary file 7 — Source data Fig. 6 [file 44319_2026_838_MOESM7_ESM.zip › Figure 6/Figure 6J/Figure 6J.tif]

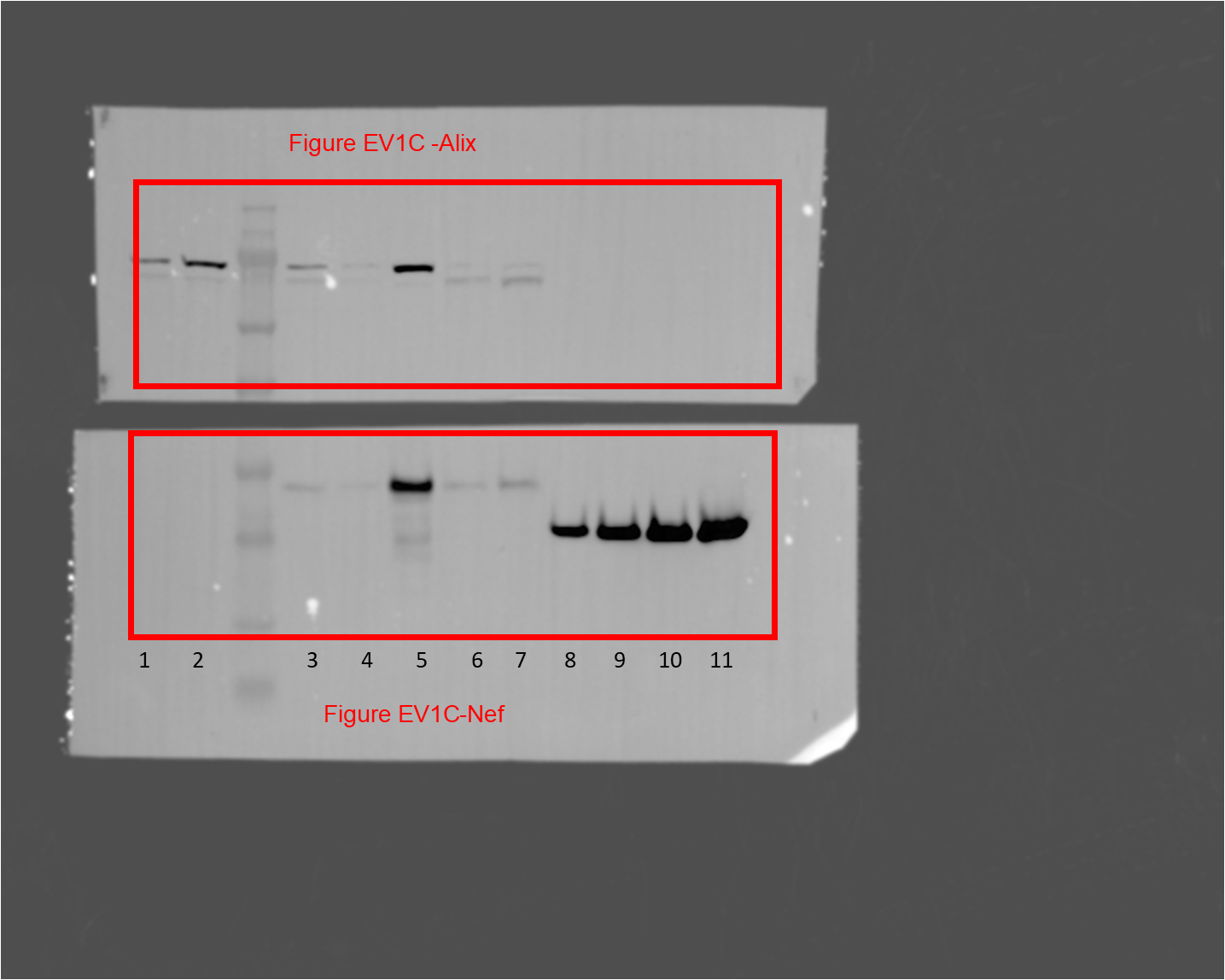

Supplement: Supplementary file 8 — Figure EV1 Source Data [file 44319_2026_838_MOESM8_ESM.zip › EV Figure 1/EV1C/EV1C.tif]
