# Supplementary material for: HIV-1 Nef generates lasting innate immune memory in haematopoietic stem and progenitor cells in vivo
Source: EMBO Rep. 2026 Jun 15;27(14):4166–92. doi: 10.1038/s44319-026-00838-w (PMC13400611; doi:10.1038/s44319-026-00838-w)
Supplement: Supplementary file 12 — Expanded View Figures [file 44319_2026_838_MOESM12_ESM.pdf]

Expanded View Figures

Figure EV1. Characterization of EVs produced by SupT1 cells.

(**A**, **B**) Size distribution of GFP-EVs (**A**) and Nef-EVs (**B**). (**C**) Western blot for assessing Nef content in Nef-EVs. Lane 1—GFP-EVs, 5 μg total protein, lane 2—GFP-EVs, 10 μg total protein, lane 3—Nef-EVs produced in HEK293 cells (10^9 particles), lane 4—Nef-EVs produced in HEK293 cells (0.5×10^9 particles), lane 5—Nef-EVs produced in SupT1 cells (0.4×10^{10} particles), lane 6—Nef-EVs produced in SupT1 cells (1×10^9 particles), lane 7—Nef-EVs produced in SupT1 cells (0.5×10^9 particles, from a different experiment), lanes 8–11—rNef (indicated amounts). (**D**, **E**) EV markers discovered during proteomics analysis of GFP-EVs (**D**) and Nef-EVs (**E**). Source data are available online for this figure.

 © The Author(s)

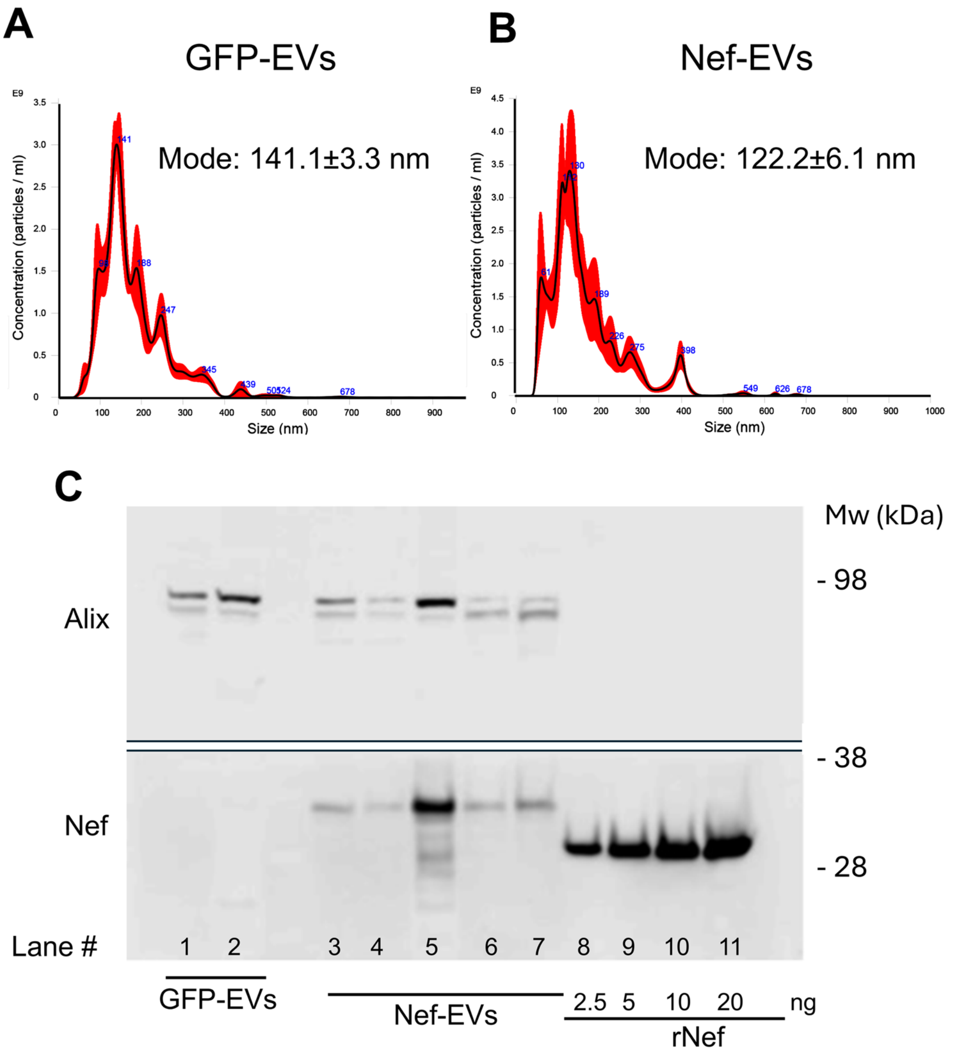

D

GFP-EVs

**EV Marker**	
Alix	+
CD9	+
CD63	+
HSP70	+
Flotillin-1	+
TSG101	+
Lamin A/C	-
Calnexin	-
MAP1LC3A	-

E

Nef-EVs

**EV Marker**	
Alix	+
CD9	+
CD63	+
HSP70	+
Flotillin-1	+
TSG101	+
Lamin A/C	-
Calnexin	-
MAP1LC3A	-

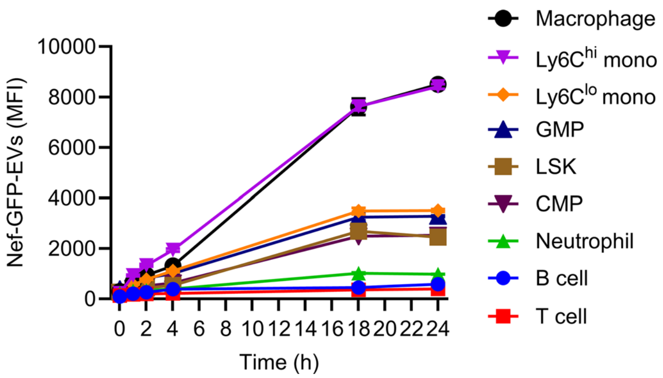

Figure EV2. Uptake of Nef-EVs by bone marrow cells in vitro.

Bone marrow freshly isolated from mice was incubated for the indicated periods of time with EVs produced in SupT1 cells transfected with GFP-Nef conjugate (4×10^{12} EVs). The relative amounts of GFP-Nef in different BM cell populations were detected by flow cytometry. Data presented as mean ± SEM from three biological replicates. Source data are available online for this figure.

 © The Author(s)

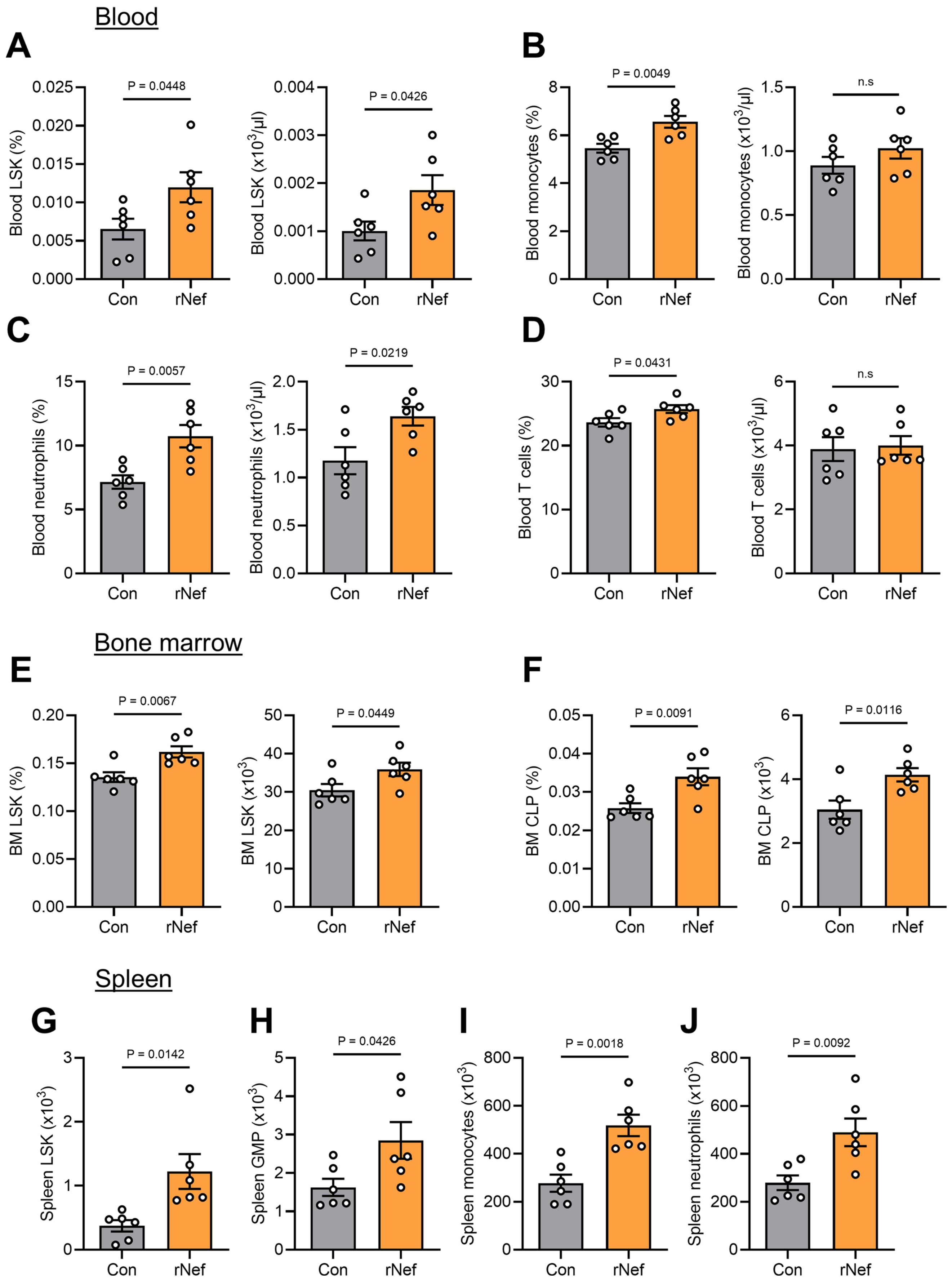
Blood
A
P = 0.0448
Blood LSK (%)
Con
rNef
P = 0.0426
Blood LSK (x10³/µl)
B
P = 0.0049
Blood monocytes (%)
n.s
Blood monocytes (x10³/µl)
C
P = 0.0057
Blood neutrophils (%)
P = 0.0219
Blood neutrophils (x10³/µl)
D
P = 0.0431
Blood T cells (%)
n.s
Blood T cells (x10³/µl)
Bone marrow
E
P = 0.0067
BM LSK (%)
P = 0.0449
BM LSK (x10³)
F
P = 0.0091
BM CLP (%)
P = 0.0116
BM CLP (x10³)
Spleen
G
P = 0.0142
Spleen LSK (x10³)
H
P = 0.0426
Spleen GMP (x10³)
I
P = 0.0018
Spleen monocytes (x10³)
J
P = 0.0092
Spleen neutrophils (x10³)

◀ **Figure EV3. Monocytosis and myeloid progenitor expansion in the blood, bone marrow, and spleen of mice treated with recombinant Nef.**

(**A–D**) Absolute and relative abundance of LSKs (**A**), monocytes (**B**), neutrophils (**C**), and T cells (**D**), and in the blood of mice after treatment with rNef (50 ng per injection every second day for 8 days) or vehicle. (**E**, **F**) Absolute and relative abundance of LSKs (**E**) and CLP (**F**) in the bone marrow of mice after treatment with rNef (50 ng per injection every second day for 8 days) or vehicle. (**G–J**) Absolute numbers of LSKs (**G**), GMP (**H**), monocytes (**I**), and neutrophils (**J**) in the spleen of mice after treatment with rNef (50 ng per injection every second day for 8 days) or vehicle. Data are presented as mean ± SEM. (**A–J**: GFP-EVs, $n = 6$; Nef-EVs, $n = 6$). Data points represent individual mice. Significance was determined by a two-tailed unpaired Student's t test (**A–J**) with calculated P values shown; n.s., not significant ($P \geq 0.05$). Source data are available online for this figure.

 © The Author(s)

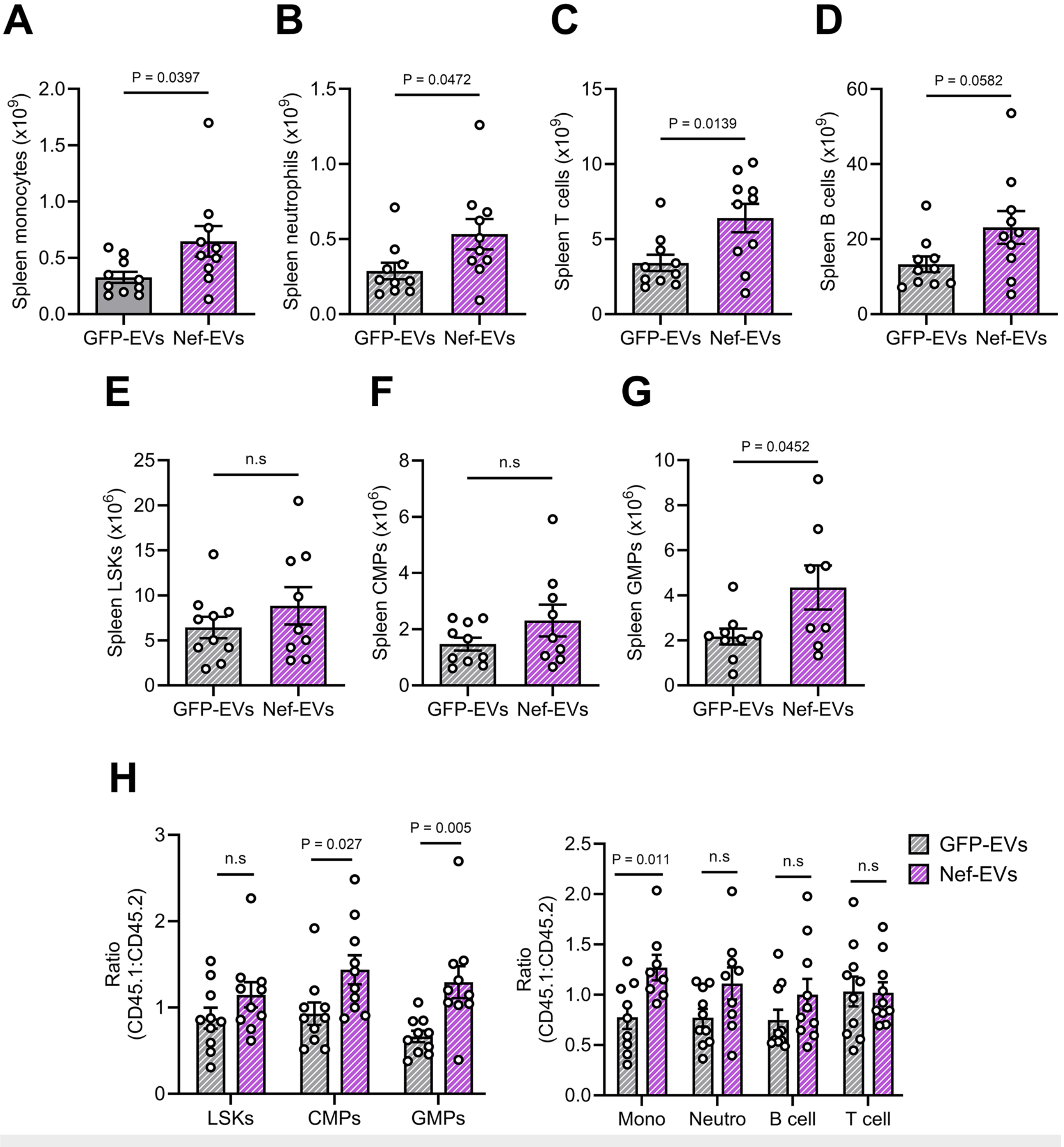

Figure EV4. Monocytosis and myeloid progenitor expansion in the spleen of recipient mice transplanted with bone marrow from Nef-EV-treated mice.

(**A–G**) Absolute number of monocytes (**A**), neutrophils (**B**), T cells (**C**), B cells (**D**), LSKs (**E**), CMPs (**F**), and GMPs (**G**) in the spleen of mice after GFP-EV or Nef-EV treatment (GFP-EVs, $n = 10$; Nef-EVs, $n = 8$–10). (**H**) Ratio of CD45.1/CD45.2 in LSKs and leukocytes in the spleen of recipient mice at week 10 (GFP-EVs, $n = 10$; Nef-EVs, $n = 10$). Data are presented as mean ± SEM. Data points represent individual mice. Significance was determined by two-tailed Student's *t* test (**A–G**) or multiple unpaired *t* tests (**H**) with calculated *P* values shown; n.s., not significant ($P \geq 0.05$). Source data are available online for this figure.

© The Author(s)
