# Supplementary material for: HIV-1 Nef generates lasting innate immune memory in haematopoietic stem and progenitor cells in vivo
Source: EMBO Rep. 2026 Jun 15;27(14):4166–92. doi: 10.1038/s44319-026-00838-w (PMC13400611; doi:10.1038/s44319-026-00838-w)
Supplement: Supplementary file 1 — Peer Review File [file 44319_2026_838_MOESM1_ESM.pdf]

EMBO reports

HIV-1 Nef Generates Lasting Innate Immune Memory in Haematopoietic Stem and Progenitor Cells In Vivo

Andrew Fleetwood, Nigora Mukhamedova, Dragana Dragoljevic, Man Lee, Yangsong Xu, Malathi Dona, Ian Hsu, Camilla Bertuzzo Veiga, Fumihiko Takeuchi, Ben Crossett, Denise Tran, Alexander Pinto, Michael Bukrinsky, Andrew Murphy, and Dmitri Sviridov

Corresponding author(s): Dmitri Sviridov (dmitri.sviridov@baker.edu.au)

Review Timeline:

Submission Date:	12th Jan 26
Editorial Decision:	6th Feb 26
Revision Received:	13th Apr 26
Editorial Decision:	20th May 26
Revision Received:	31st May 26
Accepted:	5th Jun 26

Editor: Achim Breiling

Transaction Report:

(Note: With the exception of the correction of typographical or spelling errors that could be a source of ambiguity, letters and reports are not edited. Depending on transfer agreements, referee reports obtained elsewhere may or may not be included in this compilation. Referee reports are anonymous unless the Referee chooses to sign their reports.)

Dear Prof. Sviridov,

Thank you for the submission of your manuscript to EMBO reports. I have now received reports from the three referees that were asked to evaluate your study, which can be found at the end of this email. As you will see, the referees have several comments, concerns, and suggestions, indicating that a major revision of the manuscript is necessary to allow publication of the study in EMBO reports. As the reports are below, and all points need to be addressed, I will not detail them here.

Given the constructive referee comments, I would thus like to invite you to revise your manuscript with the understanding that the concerns of the referees must be addressed in the revised manuscript and in a detailed point-by-point response. Acceptance of your manuscript will depend on a positive outcome of a second round of review. It is EMBO reports policy to allow a single round of revision only and acceptance of the manuscript will therefore depend on the completeness of your responses included in the next, final version of the manuscript.

Revised manuscripts should be submitted within three months of a request for revision. Please contact me to discuss the revision (also by video chat) if you have questions or comments regarding the revision, or should you need additional time.

When submitting your revised manuscript, please also carefully review the instructions that follow below.

PLEASE NOTE THAT upon resubmission revised manuscripts are subjected to an initial quality control prior to exposition to re-review. Upon failure in the initial quality control, the manuscripts are sent back to the authors, which may lead to delays. Frequent reasons for such a failure are the lack of the data availability section (please see below) and the presence of statistics based on n=2 (the authors are then asked to present scatter plots or provide more data points).

When submitting your revised manuscript, we will require:

1) a .docx formatted version of the final manuscript text (including legends for main figures, EV figures and tables), but without the figures included. Figure legends should be compiled at the end of the manuscript text.

2) individual production quality figure files as .eps, .tif, .jpg (one file per figure), of main figures and EV figures. Please upload these as separate, individual files upon re-submission.

The Expanded View format, which will be displayed in the main HTML of the paper in a collapsible format, has replaced the Supplementary information. You can submit up to 6 images as Expanded View. Please follow the nomenclature Figure EV1, Figure EV2 etc. The figure legend for these should be included in the main manuscript document file in a section called Expanded View Figure Legends after the main Figure Legends section. Additional Supplementary material should be supplied as a single pdf file labeled Appendix. The Appendix should have page numbers and needs to include a table of content on the first page (with page numbers) and legends for all content. Please follow the nomenclature Appendix Figure Sx, Appendix Table Sx etc. throughout the text, and also label the figures and tables according to this nomenclature.

For more details, please refer to our guide to authors:
https://link.springer.com/journal/44319/submission-guidelines#cms-Revised-submissions

Please consult our guide for figure preparation:
https://media.springernature.com/original/springer-cms/rest/v1/content/27825798/data/v1

3) a .docx formatted letter INCLUDING the reviewers' reports and your detailed point-by-point responses to their comments. As part of the EMBO Press transparent editorial process, the point-by-point response is part of the Review Process File (RPF), which will be published alongside your paper.

4) a complete author checklist, which you can download from our author guidelines (https://link.springer.com/journal/44319/submission-guidelines#cms-Revised-submissions). Please insert page numbers in the checklist to indicate where the requested information can be found in the manuscript. The completed author checklist will also be part of the RPF.

Please also follow our guidelines for the use of living organisms, and the respective reporting guidelines:
https://link.springer.com/partners/embo-press/editorial-policies#Animal%20and%20human%20studies

5) that primary datasets produced in this study (e.g. RNA-seq, ChIP-seq, structural and array data) are deposited in an appropriate public database. If no primary datasets have been deposited, please also state this in a dedicated section (e.g. 'No primary datasets have been generated and deposited'), see below.

See also: https://link.springer.com/partners/embo-press/editorial-policies#Data%20deposition

Please remember to provide a reviewer password if the datasets are not yet public.

The accession numbers and database should be listed in a formal "Data Availability" section that follows the model below. This is now mandatory (like the COI statement). Please note that the Data Availability Section is restricted to new primary data that are part of this study. This section is mandatory. As indicated above, if no primary datasets have been deposited, please state this in this section

Data availability

The datasets produced in this study are available in the following databases:

- RNA-Seq data: Gene Expression Omnibus GSE46843 (https://www.ncbi.nlm.nih.gov/geo/query/acc.cgi?acc=GSE46843)
- [data type]: [name of the resource] [accession number/identifier/doi] ([URL or identifiers.org/DATABASE:ACCESSION])

*** Note - All links should resolve to a page where the data can be accessed. ***

See also: https://link.springer.com/partners/embo-press/editorial-policies#Data%20availability%20statement

Moreover, I have these editorial requests:

6) We now request the publication of original source data with the aim of making primary data more accessible and transparent to the reader. You will receive a separate email with instructions for providing source data with your revised manuscript, including information how to upload and organize the files.

7) Our journal encourages inclusion of *data citations in the reference list* to directly cite datasets that were re-used and obtained from public databases. Data citations in the article text are distinct from normal bibliographical citations and should directly link to the database records from which the data can be accessed. In the main text, data citations are formatted as follows: "Data ref: Smith et al, 2001" or "Data ref: NCBI Sequence Read Archive PRJNA342805, 2017". In the Reference list, data citations must be labeled with "[DATASET]". A data reference must provide the database name, accession number/identifiers and a resolvable link to the landing page from which the data can be accessed at the end of the reference. Further instructions are available at: https://link.springer.com/journal/44319/submission-guidelines#cms-Reference-guidelines

8) Regarding data quantification and statistics, please make sure that the number "n" for how many independent experiments were performed, their nature (biological versus technical replicates), the bars and error bars (e.g. SEM, SD) and the test used to calculate p-values is indicated in the respective figure legends (also for EV and Appendix figures). Please also check that all the p-values are explained in the legend, and that these fit to those shown in the figure. Please provide statistical testing where applicable. Please avoid the phrase 'independent experiment' but clearly state if these were biological or technical replicates. Please also indicate (e.g. with n.s.) if testing was performed, but the differences are not significant. In case n=2, please show the data as separate datapoints without error bars and statistics. See also:
https://link.springer.com/journal/44319/submission-guidelines#cms-Figure-and-data-presentation

If n<5, please show single datapoints for diagrams.

9) Please also note our reference format:
https://link.springer.com/journal/44319/submission-guidelines#cms-Reference-guidelines

10) We updated our journal's competing interests policy in January 2022 and request authors to consider both actual and perceived competing interests. Please name this section 'Disclosure and Competing Interests Statement' and put it after the Acknowledgements section.
See: https://link.springer.com/partners/embo-press/editorial-policies#Competing%20interest%20disclosures

11) We now use CRediT to specify the contributions of each author in the journal submission system. CRediT replaces the author contribution section. Please use the free text box to provide more detailed descriptions and do NOT provide your final manuscript text file with an author contributions section. See also our guide to authors (section 'Author contributions'):
https://link.springer.com/journal/44319/submission-guidelines#cms-Revised-submissions

12) All Materials and Methods need to be described in the main text using our 'Structured Methods' format, which is required for all research articles. According to this format, the Methods section should include a Reagents and Tools Table (listing key reagents, experimental models, software, and relevant equipment and including their sources and relevant identifiers), uploaded as separate file, and a Methods section in which we encourage the authors to describe their methods using a step-by-step protocol format with bullet points, to facilitate the adoption of the methodologies across labs. More information on how to adhere to this format as well as downloadable templates (.doc) for the Reagents and Tools Table can be found in our author guidelines (section 'Structured Methods'):

https://link.springer.com/journal/44319/submission-guidelines#cms-Manuscript-organisation-and-formatting

Please add all primer or antibody information directly to the Reagents and Tools table.

13) Please reduce the number of keywords to five and order the manuscript sections like this, using only these names:
Title page - Abstract (max. 175 words) - Keywords - Introduction - Results - Discussion - Methods - Data availability section - Acknowledgements (please include here all the funding information) - Disclosure and Competing Interests Statement - References - Figure legends - Expanded View Figure legends

14) Please make sure that all the funding information is also entered into the online submission system and that it is complete and similar to the one in the acknowledgement section of the manuscript text file.

We would also welcome the submission of cover suggestions, or motifs to be used by our Graphics Illustrator in designing a cover.

As part of the EMBO publication's Transparent Editorial Process, EMBO reports publishes online a Review Process File (RPF) to accompany accepted manuscripts. This File will be published in conjunction with your paper and will include the referee reports, your point-by-point response and all pertinent correspondence relating to the manuscript.

You are able to opt out of this by letting the editorial office know (emboreports@embo.org). If you do opt out, the Review Process File link will point to the following statement: "No Review Process File is available with this article, as the authors have chosen not to make the review process public in this case."

I look forward to seeing a revised form of your manuscript when it is ready.

Yours sincerely,

Achim Breiling
Senior Editor
EMBO Reports

Referee #1:

The viral Nef protein drives HIV pathogenesis by affecting the function of infected cells but also by triggering the release of extracellular vesicles (EVs) that can impact the function of uninfected bystander cells. Following up on previous work by the same investigators, the current manuscript by Fleetwood, Mukhamedova and colleagues explores in more detail the long-lasting effect EVs from Nef-expressing cells exert on myeloid cells in vitro and in vivo described by Dubrovsky et al., 2022. The results reveal that treatment of mice with these EVs induces reprogramming of genes involved in energy and lipid metabolism that are consistent with the signatures observed upon the induction of trained immunity.

Importantly, transplantation of bone marrow of EV-treated mice revealed similar phenotypes in terms of cell differentiation and inflammatory potential, indicating that EV treatment indices memory in hematopoetic cells. An experiment in which BMDM are exposed with EVs in vitro suggests that interference with the metabolic changes induced by EVs from Nef expressing cells reduces their proinflammatory cytokine response to these EVs. The study addresses a relevant topic and provides important new insights but also has a number of important shortcomings.

1) While some of the findings presented are confirmatory of their previous work, the authors provide new insight on the molecular signature induced by EV treatment and present the fascinating results that bone marrow transplantation can transfer the features acquired by EC treatment on the donor animal. To establish a mechanistic link between these two observations, the authors present the siGPI experiment in Fig.4., which however is conducted exclusively in vitro and therefore does not really address the question. Experiments in the transplantation system (e.g. assessing the impact of targeted gene editing of the transferred cells) need to be included to test if the metabolic changes observed in EC treated animals really drive the establishment of long-lasting immune memory. This is the key novelty and conclusion of the study and needs to be further developed to make a convincing case.

2) Essential information on the quality controls for EV preps is missing (e.g. analysis of content for Nef and other cargo). According to the method section, 2µg EVs per animal were used. How does this compare to the amounts of EVs present in the serum of PLWH? Assessing if EVs isolated from the sera of PLWH can also induce immunological memory would be essential to establish the relevance of the phenomenon described in the manuscript.

3) Literature from many labs, which is largely ignored by the authors except their own work, has established that Nef can promote the formation of EVs, is incorporated in and present at the surface of EVs, and alters the EV content including an enrichment in proinflammatory cytokines. The authors interpret their results as a direct consequence of the presence of the Nef protein in their EVs but have not tested this hypothesis (e.g. is Nef detected in target cells, how do mutant Nef proteins with defects in specific functional motifs perform?). Experiments to dissect which is the active component of memory-inducing EVs would be essential. Irrespectively whether as trigger of EV release or active component (see comment below), it is important to test if this effect is limited to the single Nef variant used herein (from HIV-1 SF2) or is conserved among lentiviral Nef proteins.

Referee #2:

In this manuscript, the authors investigate whether the HIV-1 accessory protein Nef, delivered via extracellular vesicles (EV), is sufficient to induce long-lasting innate immune reprogramming in vivo. Using a combination of EV exposure experiments, multi-omic profiling, and competitive bone marrow transplantation assays, the authors propose that exosome-associated Nef (exNef) induces a durable, cell-intrinsic form of inflammatory memory consistent with trained immunity. The study addresses an important and timely question at the interface of viral immunology, innate immune memory, and chronic inflammation, and the experimental scope is ambitious.

While the work contains several interesting observations and employs technically demanding approaches, the mechanistic conclusions currently exceed the strength of the presented data, and several key methodological issues substantially weaken the central claims. In particular, concerns regarding EV characterization, cross-species experimental design, and lack of evidence for direct bone marrow targeting need to be addressed before the study can be considered for publication in EMBO Reports.

Major Concerns:
1. Insufficient characterization of EV and incomplete adherence to MISEV guidelines. The study relies on the biological activity of exosome-associated Nef; however, EV characterization remains incomplete. Key aspects recommended by MISEV2018/2023-including rigorous assessment of EV purity, exclusion of non-vesicular protein contaminants, and normalization using orthogonal EV metrics-are not sufficiently addressed. In several instances, EV dosing appears to rely primarily on total protein content, which is discouraged in the absence of particle-based normalization.

2. The manuscript does not adequately acknowledge prior seminal work demonstrating the release of Nef in extracellular vesicles and its functional effects on T cells and myeloid cells, including monocytes and macrophages. Several important studies in this area are not cited, while more recent or less comprehensive reports are referenced instead. A more balanced discussion that integrates prior work is necessary.

3. Use of human SupT1-derived EVs in mouse models.
A major concern is the use of EVs derived from a human T cell line (SupT1) for in vivo mouse experiments. Human EVs inherently contain xenogeneic proteins, lipids, and glycans that may elicit innate immune activation in mice independent of Nef. This raises the possibility that the observed inflammatory and myelopoietic effects at least in part reflect non-specific stimulation analogous to a mixed lymphocyte reaction rather than a Nef-specific mechanism. The often relatively modest magnitude of many effects would be consistent with such non-specific stimulation.

Therefore, essential controls to address this issue should be included:
EVs derived from murine cells of same genetic background as background control,
exNef containing Nef mutants deficient in known functional domains (e.g. in PxxP),
or genetic/biochemical depletion of Nef from EV preparations.

4. Overinterpretation of competitive bone marrow transplantation.
The competitive bone marrow transplantation experiments demonstrate persistence and cell-intrinsic effects; however, they do not, on their own, establish trained immunity. Trained immunity implies stable epigenetic reprogramming of hematopoietic progenitors that causally underlies enhanced secondary responses. In this study epigenetic changes are inferred largely (i) from correlative chromatin accessibility and transcriptional profiling. (ii) no functional perturbation of chromatin-modifying pathways is provided. (iii) no direct demonstration of stable epigenetic marks (e.g. locus-specific histone modifications) is shown.
Thus, while the data are consistent with innate immune reprogramming, the assertion that trained immunity has been established is premature.

5. Lack of evidence that injected exNef-EVs reach target cells.
A critical mechanistic gap is the absence of evidence that intravenously injected exNef-EVs reach the bone marrow or are taken up by hematopoietic stem and progenitor cells. No biodistribution studies, EV labeling experiments, or uptake analyses are provided to support this key assumption. As a result, alternative explanations, such as indirect systemic effects mediated by circulating cytokines or peripheral immune cells cannot be excluded.

6. Mechanistic conclusions exceed the presented evidence.
Although the authors present extensive datasets, the mechanistic link between Nef-containing EVs, metabolic rewiring, chromatin remodeling, and durable inflammatory memory remains largely associative. Interventions targeting glycolysis or cholesterol metabolism provide partial support but do not establish a specific causal pathway connecting Nef to epigenetic reprogramming.

Minor Comments:
The terminology "trained immunity" should be used more cautiously throughout the manuscript and clearly distinguished from long-term inflammatory priming or persistence.

The discussion would benefit from a more explicit consideration of alternative explanations, particularly indirect systemic effects of EV administration.
Several conclusions in the Discussion section would benefit from clearer separation between experimentally supported findings and speculative interpretations.

Overall Recommendation: major revision
This study addresses an important question and contains several interesting observations; however, substantial additional experimental support and/or a more cautious framing of the conclusions are required. In its current form, the manuscript falls short of the mechanistic rigor expected for EMBO Reports. Addressing the concerns outlined above would significantly strengthen the work and clarify its conceptual contribution.

Referee #3:

The study addresses an important and timely question regarding the role of extracellular Nef (exNef) in the induction of trained immunity and its potential contribution to persistent inflammation in HIV. While the experimental observations are interesting, several conceptual and methodological aspects require further clarification to strengthen the manuscript and clearly delineate its novelty and impact.

A previous study by Dubrovsky et al. (2022) proposed that exNef induces trained immunity, thereby linking this phenomenon to persistent inflammation and HIV-associated comorbidities. The authors of the current study should more clearly emphasize the novelty of their work, as the central hypothesis appears largely overlapping with this prior publication.

The authors should also clarify the rationale behind their experimental design, specifically the intravenous infusion of extracellular vesicles containing Nef three times per week for two weeks. This approach differs from classical trained immunity mouse models, such as those using β-glucan or BCG, and the reasoning for this dosing regimen and route of administration should be better justified.

The authors associate the observed transcriptional and epigenetic changes in the myeloid compartment with trained immunity. While these signatures are consistent with previously described trained immunity profiles, definitive functional proof is still lacking. To strengthen this claim, the authors should consider assessing the response of exNef-trained mice to a secondary heterologous challenge and comparing it with appropriate control groups.

For the ex vivo training experiments in which bone marrow-derived macrophages (BMDMs) were treated with exNef or control for 48 hours, the authors should clarify why the stimulus was removed only after 48 hours of exposure. Additionally, the effects of LPS as a secondary stimulus on cytokine production (TNF and IL-6) in the absence of silencing constructs are not shown (Figure 4). This control is essential to establish exNef as a bona fide inducer of trained immunity.

The authors should also consider discussing the mechanisms underlying the effects of exNef on the bone marrow compartment. Do the authors propose that exNef directly reaches the bone marrow and is sensed by hematopoietic stem and progenitor cells (HSPCs), or is the effect indirect, mediated through systemically induced soluble factors such as cytokines that subsequently act on the bone marrow?

Minor Comments:

The statistical methods used are not consistently stated in the figure legends and should be clearly indicated for all figures.

Dr. Achim Breiling

Senior Editor, *EMBO Reports*

Melbourne, April 13, 2026

Submission ID: EMBOR-2025-63451V1

Dear Dr. Breiling,

Thank you very much for your letter of February 2nd, 2026, and careful review of our manuscript entitled "**HIV Infection Generates Lasting Innate Immune Memory in Haematopoietic Stem and Progenitor Cells In Vivo"** (Submission ID EMBOR-2025-63451V1).

We appreciate the reviewers' comments; they were professional and constructive, and their implementation has resulted in a significant improvement of the manuscript. In brief, the following major changes were introduced:

1. EVs produced by SupT1 cells were thoroughly characterized and the findings are now presented in Fig. EV1 and corresponding text.
2. Proteomics analysis of EVs has been implemented and the findings are now presented in Table EV1 and corresponding text.
3. The effects of Nef-EV on haematopoiesis have been compared with that of naked recombinant Nef and the findings are now presented in Fig. EV3 and corresponding text.
4. Uptake of Nef-EV by bone marrow cells was analyzed and the findings are now presented in Fig. EV2 and corresponding text.
5. The effects of Nef-EV on the formation of inflammatory memory were compared with that of Nef(G2A)-EV, an inactive variant of Nef. The findings are now presented in Fig. 4G and corresponding text.
6. The effects of Nef-EV on the formation of inflammatory memory were compared with that of EVs isolated from plasma of PLWH and uninfected individuals (the samples were obtained from our previously published study). The findings are now presented in Fig. 4H and corresponding text.
7. Responding to the critique of the referees about overinterpretation of the findings, parts of the Introduction and Discussion sections were re-written and the Limitations section was thoroughly revised and expanded.

Baker Heart and Diabetes Institute
75 Commercial Road, Melbourne Victoria 3004 Australia
T +61 3 8532 1111 **F** +61 3 8532 1100 **E** melbourne@baker.edu.au **W** www.baker.edu.au
PO Box 6492, Melbourne Victoria 3004 Australia **ABN: 98 131 762 948**

8. We also revised the paper to present views from other research laboratories on the subject of this study and include citations to papers from these laboratories.
9. Numerous textual changes were made in the paper in response to comments of the referees.

We believe that we have fully addressed all comments of the reviewers. **Detailed point-by-point responses to the comments are included with this submission.**

Please note that generation of proteomics data required adding two new co-authors to the paper, Dr. Ben Crossett and Dr. Denise Tran. I confirm that I hold a written consent of all authors to their inclusion.

Sincerely yours,

Dmitri Sviridov

Andrew Murphy

Point-by-point response to the comments of the reviewers

Referee #1:

1) While some of the findings presented are confirmatory of their previous work, the authors provide new insight on the molecular signature induced by EV treatment and present the fascinating results that bone marrow transplantation can transfer the features acquired by EC treatment on the donor animal. To establish a mechanistic link between these two observations, the authors present the siGPI experiment in Fig.4., which however is conducted exclusively in vitro and therefore does not really address the question. Experiments in the transplantation system (e.g. assessing the impact of targeted gene editing of the transferred cells) need to be included to test if the metabolic changes observed in EC treated animals really drive the establishment of long-lasting immune memory. This is the key novelty and conclusion of the study and needs to be further developed to make a convincing case.

Response:

We appreciate the reviewer's suggestion and agree that directly testing the causal role of glycolytic reprogramming *in vivo* would add significant mechanistic value. However, we have carefully considered the available approaches and identified substantial obstacles to each.

Pharmacological inhibition of glycolysis, while feasible, exerts broad effects on systemic metabolism and inflammation through mechanisms independent of trained immunity or the haematopoietic cell populations under study, which would confound interpretation of any results obtained.

Genetic approaches present equally serious challenges. Global deletion of essential glycolytic enzymes is lethal. Tissue-specific conditional knockout in early myeloid precursors is conceptually attractive, but permanent disruption of GPI1 in haematopoietic stem and progenitor cells would be expected to severely impair their survival, proliferation, and engraftment, as glycolysis is required for basal haematopoietic function. Such an experiment would therefore test fundamental haematopoietic viability rather than the specific contribution of metabolic rewiring to immune memory establishment. The only precedent for this approach, T cell-specific GPI1 knockout by Wu et al. (Cell 2020; 182:641–654), demonstrated that cell survival was highly context-dependent, and how individual myeloid progenitor populations would respond to GPI1 deletion remains entirely unknown, making the experimental outcome unpredictable.

We therefore consider rigorous investigation of this question to be beyond the scope of the current study. In recognition of this limitation, we have added a clear limitations statement to the manuscript (p. 20, lines 2-14) acknowledging that the causal relationship between glycolytic reprogramming and trained immunity induction remains to be established in future work.

“The mechanistic studies presented here, while supporting the proposed pathway, remain largely associative, and the precise molecular mechanisms responsible for maintaining Nef-induced TRIM have yet to be defined. The causal contribution of glycolytic reprogramming to TRIM formation was examined *in vitro*, as *in vivo* testing presents fundamental interpretive obstacles. Global inhibition of glycolysis exerts broad effects on systemic metabolism and inflammation through mechanisms independent of trained immunity or the haematopoietic cell populations under study, confounding interpretation of any results obtained. Tissue-specific approaches, such as conditional knockout of GPI1 in myeloid progenitors analogous to the T cell-specific model described by Wu et al. (2020), face a different problem: because glycolysis is required for basal haematopoietic function, genetic disruption of essential glycolytic enzymes in progenitor cells would be expected to impair survival, proliferation, and engraftment, testing fundamental haematopoietic viability rather than the specific role of metabolic rewiring in immune memory establishment.”

2) Essential information on the quality controls for EV preps is missing (e.g. analysis of content for Nef and other cargo). According to the method section, 2µg EVs per animal were used. How does this compare to the amounts of EVs present in the serum of PLWH? Assessing if EVs isolated from the sera of PLWH can also induce immunological memory would be essential to establish the relevance of the phenomenon described in the manuscript.

Response:

1. We apologise for not including EV characterization data and thank the reviewer for pointing at this significant oversight. This information is now provided in Fig. EV1 and in the text in p. 5, lines 10-21. It reads:

“Details of isolation of EVs carrying Nef or green fluorescent protein (GFP-EVs, control EVs) are provided in the Methods section. The predominant size of Nef-EVs and GFP-EVs was, correspondingly, 122±6 nm and 141±3 nm (Fig. EV1 A,B). Both types of EVs contained an EV marker Alix (Fig. EV1C, lanes 1-7), and Nef-EVs contained Nef (Fig. EV1C, lanes 5-7) while no Nef was detected in GFP-EVs (Fig. EV1C, lanes 1-2). The concentration of Nef in Nef-EVs was evaluated by densitometric analysis comparing the bands of Nef-EVs with titrated recombinant Nef (Fig. EV1C, lanes 8-11) and was usually ~ 1 ng of Nef per 109 particles. The EVs were further characterized by identification of EV markers in the proteomics dataset of GFP-EVs and Nef-EVs (see below). Six biomarkers of EVs recommended by ISEV (Welsh et al, 2024) were identified in both GFP-EVs and Nef-EVs, while markers of nucleus (Lamin), endoplasmic reticulum (Calnexin) and microtubules (MAP1LC3A) were not found (Fig. EV1 D,E).”

2. Responding to the second part of this comment, we now present the quantity of the injected EVs as their numbers and Nef concentration; we also compare the expected Nef concentration with that in blood of PLWH. This information is provided in p. 6, lines 16-20:

“We injected 10^9 EVs, containing ∼1 ng of Nef, which should result in the initial level of Nef in plasma of approximately 0.8 ng/mL, which is below levels of Nef detected in PLWH, 6–12 ng/mL (Ferdin *et al*, 2018; Raymond *et al*, 2011). The dosage and regimen were based on the results of our previous study (Mukhamedova *et al.*, 2019).”

3. Responding to the third part of this comment, we conducted experiments with EVs isolated from pooled plasma samples of PLWH and uninfected subjects. The results are shown in Fig. 4H and in text in p. 13, lines 5-11:

“Finally, we isolated EVs from pooled plasma samples of PLWH and uninfected individuals; these samples were previously characterized and tested for induction of acute inflammation in our earlier study (Mukhamedova *et al.*, 2019). Using the same *in vitro* model of trained immunity, we found that IL-6 and TNFα production from BMDM exposed to EVs from PLWH was significantly greater than from BMDMs exposed to EVs from uninfected individuals (Fig. 4I,J), demonstrating that circulating EVs from PLWH carry the capacity to induce trained immunity *ex vivo*.”

3) Literature from many labs, which is largely ignored by the authors except their own work, has established that Nef can promote the formation of EVs, is incorporated in and present at the surface of Evs, and alters the EV content including an enrichment in proinflammatory cytokines. The authors interpret their results as a direct consequence of the presence of the Nef protein in their Evs but have not tested this hypothesis (e.g. is Nef detected in target cells, how do mutant Nef proteins with defects in specific functional motifs perform?). Experiments to dissect which is the active component of memory-inducing Evs would be essential. Irrespectively whether as trigger of EV release or active component (see comment below), it is important to test if this effect is limited to the single Nef variant used herein (from HIV-1 SF2) or is conserved among lentiviral Nef proteins.

Response:

1. We accept the criticism of insufficient attention to the studies from other laboratories. The section on Nef-containing EVs is now expanded and most important studies from other laboratories cited. Please see p. 3, lines 10-17.

“A significant contributor to chronic inflammation in PLWH is the HIV-1 protein Nef (Mukhamedova *et al*, 2019), which is synthesized in infected cells in viral reservoirs. Nef promotes the secretion of inflammatory cytokines by these cells, moreover, Nef itself is incorporated into EVs released from the infected cells into systemic as well as brain circulation (Aiello *et al*, 2021; Lavrin *et al*, 2025; McNamara *et al*, 2018; Puzar Dominkus *et al*, 2017). These EVs are released independently of viral particle production (Lenassi *et al*, 2010); they

contain Nef both as a cargo and on the surface of the EV (Vanpouille *et al*, 2024) and affect the metabolism of bystander cells (Arenaccio *et al*, 2014a; Khan *et al*, 2016).”

2. We agree that demonstration of the ability of BM cells to take up exNef is important for interpretation of the results of this study. This experiment has been conducted, and the results are shown in Fig. EV2 and in text, at p. 7, lines 9-17.

“To investigate uptake of Nef-EVs by the different cell types present in the marrow, we incubated BM with GFP-tagged Nef-EVs (Nef-GFP-EV) as described previously (Mukhamedova *et al.*, 2019) and measured cellular uptake over time by flow cytometry. As expected, monocytes and macrophages were the main targets for Nef-EVs uptake, followed by the myeloid progenitors GMP and CMP, as well as LSK (Fig EV2). Both T and B cells were only weakly positive for Nef-GFP-EV. With the exception of neutrophils, the preferential uptake of Nef-GFP-EV by mature myeloid cells and their progenitors is consistent with the broad expansion of these subsets observed in the BM following challenge with Nef-EVs.”

3. We agree that transfection of SupT1 cells with Nef can trigger synthesis and incorporation into EVs of factors other than Nef that can affect innate immunity. In response, we performed unbiased proteomics analysis of EVs. This analysis identified 110 proteins differentially expressed in exNef, however, according to DAVID functional allocation, only 4 of them were related to inflammation and innate immunity. It needs to be pointed out that they are human proteins that should not cross-react if delivered to murine cells. We also include previously unpublished findings showing that naked recombinant Nef has broadly similar inflammatory effects as exNef. Finaly, we present new findings with inactive Nef variant Nef(G2A); this Nef variant did not reproduce the effects of WT Nef on inflammation and trained immunity.

The results are now presented in Table EV1 and Fig. 4G and described in the text from p. 5, line 22 to page 6, line 7, from p.12, line 20 to p. 13, line2, and discussed in the text from p. 17, line 19 to p.18, line 3.

“We assumed that the effects of infusion of Nef-EVs could be ascribed to Nef; however, it could not be excluded that transfection of SupT1 cells with Nef resulted in the incorporation of an unidentified factor(s) into Nef-EVs, which was absent in GFP-EVs and was the true cause of the observed effects. To resolve this uncertainty, we conducted an unbiased proteomics analysis of Nef-EVs and compared proteins with control EVs. The list of proteins found in Nef-EVs, but not in the control EVs, is shown in Table EV1. Functional annotation of these proteins revealed that out of 110 proteins uniquely found in Nef-EVs, only 4 were involved in innate immunity pathways: Myd88, TBK1, TRAF6 and IFNG. It is important to recognize that these are human proteins, and as was shown for IFNG, have high species specificity and are unlikely to affect mouse cells (Kumar et al, 1989).”

“To further characterise the formation of inflammatory memory, we used isolated BMDMs to compare the effects of Nef-EVs with EVs produced by Sup T1 cells expressing a myristoylation-deficient mutant of Nef, Nef(G2A). This Nef mutant lacks the N-terminal myristoylation signal, is excluded from EVs (Arenaccio *et al*, 2014b), and is inactive in

modifying cholesterol metabolism (Mujawar *et al.*, 2006). When tested in the *in vitro* training model, Nef(G2A)-EVs failed to induce durable inflammatory memory, producing no observable increase in LPS-induced IL-6 and TNFα release five days after EV treatment (Fig. 4G,H)."

"The following findings support the role of Nef in the formation of TRIM: (*i*) Nef-EVs were taken up by BM cells; (*ii*) there was a notable similarity between the effect of Nef-EVs on BM cells *in vivo* and human and murine macrophages *in vitro* and *ex vivo* (Dubrovsky *et al.*, 2022; Mukhamedova *et al.*, 2019); (*iii*) the effects of Nef-EVs and rNef on haematopoiesis *in vivo* and inflammation *in vivo* and *in vitro* where qualitatively similar; (*iv*) EVs produced by cells expressing inactive variant of Nef, Nef(G2A), did not cause formation of inflammatory memory; (*v*) proteomics analysis of Nef-EVs did not reveal another probable candidate for TRIM formation. Collectively these findings make it likely that the effects of Nef result from direct interaction of Nef with BM cells, rather than mediated by systemic soluble factors."

4. We appreciate the reviewer raising this point. We have previously tested Nef proteins isolated from multiple HIV and SIV variants across a panel of assays examining cholesterol homeostasis, the principal Nef activity underlying its pro-inflammatory effects (Mujawar et al., PLoS Biol. 2006; 4:e365; Asztalos et al., J Infect Dis. 2010; 202:614–623). In none of these assays did we observe qualitative differences in activity between Nef isolates, suggesting that the effects characterized here are a conserved property of Nef rather than specific to the isolate used.

Referee #2:

While the work contains several interesting observations and employs technically demanding approaches, the mechanistic conclusions currently exceed the strength of the presented data, and several key methodological issues substantially weaken the central claims. In particular, concerns regarding EV characterization, cross-species experimental design, and lack of evidence for direct bone marrow targeting need to be addressed before the study can be considered for publication in EMBO Reports.

We address these issues in our responses to the specific comments below.

Major Concerns:

1. Insufficient characterization of EV and incomplete adherence to MISEV guidelines. The study relies on the biological activity of exosome-associated Nef; however, EV characterization remains incomplete. Key aspects recommended by MISEV2018/2023-including rigorous assessment of EV purity, exclusion of non-vesicular protein contaminants,

and normalization using orthogonal EV metrics-are not sufficiently addressed. In several instances, EV dosing appears to rely primarily on total protein content, which is discouraged in the absence of particle-based normalization.

Response:

We apologise for not including EV characterization data and thank the reviewer for pointing at this significant oversight. This information is now provided in Fig. EV1 and in the text p. 5, lines 10-21. It reads:

"Details of isolation of EVs carrying Nef or green fluorescent protein (GFP-EVs, control EVs) are provided in the Methods section. The predominant size of Nef-EVs and GFP-EVs was, correspondingly, 122±6 nm and 141±3 nm (Fig. EV1 A,B). Both types of EVs contained an EV marker Alix (Fig. EV1C, lanes 1-7), and Nef-EVs contained Nef (Fig. EV1C, lanes 5-7) while no Nef was detected in GFP-EVs (Fig. EV1C, lanes 1-2). The concentration of Nef in Nef-EVs was evaluated by densitometric analysis comparing the bands of Nef-EVs with titrated recombinant Nef (Fig. EV1C, lanes 8-11) and was usually ～1 ng of Nef per 109 particles. The EVs were further characterized by identification of EV markers in the proteomics dataset of GFP-EVs and Nef-EVs (see below). Six biomarkers of EVs recommended by ISEV (Welsh et al, 2024) were identified in both GFP-EVs and Nef-EVs, while markers of nucleus (Lamin), endoplasmic reticulum (Calnexin) and microtubules (MAP1LC3A) were not found (Fig. EV1 D,E).

Further, we now present the quantity of the injected Evs both as their numbers and Nef concentration; we also compare the expected Nef concentration with that in blood of PLWH. This information is provided in p. 6, lines 16-20:

"We injected 109 EVs, containing ～1 ng of Nef, which should result in the initial level of Nef in plasma of approximately 0.8 ng/mL, which is below levels of Nef detected in PLWH, 6–12 ng/mL (Ferdin et al, 2018; Raymond et al, 2011). The dosage and regimen were based on the results of our previous study (Mukhamedova et al., 2019)."

2. The manuscript does not adequately acknowledge prior seminal work demonstrating the release of Nef in extracellular vesicles and its functional effects on T cells and myeloid cells, including monocytes and macrophages. Several important studies in this area are not cited, while more recent or less comprehensive reports are referenced instead. A more balanced discussion that integrates prior work is necessary.

Response:

We accept the criticism of insufficient attention to the studies from other laboratories. The

section on Nef-containing EVs is now expanded and studies from other laboratories cited. Please see p. 3, lines 10-17.

"A significant contributor to chronic inflammation in PLWH is the HIV-1 protein Nef (Mukhamedova *et al*, 2019), which is synthesized in infected cells in viral reservoirs. Nef promotes the secretion of inflammatory cytokines by these cells, moreover, Nef itself is incorporated into EVs released from the infected cells into systemic as well as brain circulation (Aiello *et al*, 2021; Lavrin *et al*, 2025; McNamara *et al*, 2018; Puzar Dominkus *et al*, 2017). These EVs are released independently of viral particle production (Lenassi *et al*, 2010); they contain Nef both as a cargo and on the surface of the EV (Vanpouille *et al*, 2024) and affect the metabolism of bystander cells (Arenaccio *et al*, 2014a; Khan *et al*, 2016)."

3. Use of human SupT1-derived Evs in mouse models.

A major concern is the use of Evs derived from a human T cell line (SupT1) for in vivo mouse experiments. Human Evs inherently contain xenogeneic proteins, lipids, and glycans that may elicit innate immune activation in mice independent of Nef. This raises the possibility that the observed inflammatory and myelopoietic effects at least in part reflect non-specific stimulation analogous to a mixed lymphocyte reaction rather than a Nef-specific mechanism. The often relatively modest magnitude of many effects would be consistent with such non-specific stimulation.

Therefore, essential controls to address this issue should be included: Evs derived from murine cells of same genetic background as background control, exNef containing Nef mutants deficient in known functional domains (e.g. in PxxP), or genetic/biochemical depletion of Nef from EV preparations.

Response:

We thank the reviewer for raising this important point and have considered it carefully. The reviewer's concern that human-derived EVs may elicit non-specific innate immune activation in mice independent of Nef is addressed directly by our experimental design. In all experiments, EVs produced in the same human cell line (SupT1) expressing GFP rather than Nef served as the comparator control. These GFP-EVs are identical to Nef-EVs in their cellular origin, production conditions, lipid composition, and content of human xenogeneic proteins, lipids, and glycans, differing mostly in the absence of Nef. Critically, GFP-EV-treated animals showed no inflammatory or myelopoietic effects distinguishable from untreated controls in any assay. If xenogeneic components of human EVs were sufficient to drive the observed phenotypes non-specifically, this would have been apparent in the GFP-EV group, and it was not. The GFP-EV control therefore directly excludes non-specific stimulation as an explanation for our findings.

We note that this design mirrors our previous studies using EVs produced in HEK293 cells, another human cell line, where the same pattern was observed: Nef-EVs induced

inflammation while GFP-EVs produced no response above baseline (Mukhamedova et al., 2019).

In addition, and consistent with the reviewer's suggestion, we conducted experiments using EVs produced in cells expressing the myristoylation-deficient Nef mutant Nef(G2A). This mutant is not myristoylated, does not associate with the plasma membrane, and is not incorporated into EVs, providing a second Nef-specific control that further dissociates any EV carrier effect from the activity of Nef itself. Nef(G2A)-EVs failed to induce trained immunity in any assay, results that are presented in Fig. 4G and described in text from p.12, line 20 to p. 13, line4, and the essential role of Nef is further discussed in the text from p. 17, line 19 to p.18, line 5.

"To further characterise the formation of inflammatory memory, we used isolated BMDMs to compare the effects of Nef-EVs with EVs produced by Sup T1 cells expressing a myristoylation-deficient mutant of Nef, Nef(G2A). This Nef mutant lacks the N-terminal myristoylation signal, is excluded from EVs (Arenaccio et al, 2014b), and is inactive in modifying cholesterol metabolism (Mujawar et al., 2006). When tested in the in vitro training model, Nef(G2A)-EVs failed to induce durable inflammatory memory, producing no observable increase in LPS-induced IL-6 and TNFα release five days after EV treatment (Fig. 4G,H). These findings demonstrate that EV incorporation of Nef is necessary for the induction of trained immunity, directly implicating Nef as the active cargo responsible for reprogramming macrophage inflammatory responses."

"The following findings support the role of Nef in the formation of TRIM: (i) Nef-EVs were taken up by BM cells; (ii) there was a notable similarity between the effect of Nef-EVs on BM cells in vivo and human and murine macrophages in vitro and ex vivo (Dubrovsky et al., 2022; Mukhamedova et al., 2019); (iii) the effects of Nef-EVs and rNef on haematopoiesis in vivo and inflammation in vivo and in vitro where qualitatively similar; (iv) EVs produced by cells expressing inactive variant of Nef, Nef(G2A), did not cause formation of inflammatory memory; (v) proteomics analysis of Nef-EVs did not reveal another probable candidate for TRIM formation. Collectively these findings make it likely that the effects of Nef result from direct interaction of Nef with BM cells, rather than mediated by systemic soluble factors. This is further supported by the transplantation experiments, where TRIM phenotype was associated only with donor cells exposed to Nef-EVs."

4. Overinterpretation of competitive bone marrow transplantation.

The competitive bone marrow transplantation experiments demonstrate persistence and cell-intrinsic effects; however, they do not, on their own, establish trained immunity. Trained immunity implies stable epigenetic reprogramming of hematopoietic progenitors that causally underlies enhanced secondary responses. In this study epigenetic changes are inferred largely (i) from correlative chromatin accessibility and transcriptional profiling. (ii) no functional perturbation of chromatin-modifying pathways is provided. (iii) no direct demonstration of stable epigenetic marks (e.g. locus-specific histone modifications) is shown.

Thus, while the data are consistent with innate immune reprogramming, the assertion that trained immunity has been established is premature.

Response:

We agree with this criticism and combined response to this comment with the response to a similar criticism in comment #6, please see below.

5. Lack of evidence that injected exNef-Evs reach target cells.

A critical mechanistic gap is the absence of evidence that intravenously injected exNef-Evs reach the bone marrow or are taken up by hematopoietic stem and progenitor cells. No biodistribution studies, EV 11abelling experiments, or uptake analyses are provided to support this key assumption. As a result, alternative explanations, such as indirect systemic effects mediated by circulating cytokines or peripheral immune cells cannot be excluded.

Response:

We agree with this comment and experimentally investigated EV uptake by different populations of BM cells. The results are shown in Fig. EV2, and described in at p. 7, lines 9-17.

"To investigate uptake of Nef-EVs by the different cell types present in the marrow, we incubated BM with GFP-tagged Nef-EVs (Nef-GFP-EV) as described previously (Mukhamedova *et al.*, 2019) and measured cellular uptake over time by flow cytometry. As expected, monocytes and macrophages were the main targets for Nef-EVs uptake, followed by the myeloid progenitors GMP and CMP, as well as LSK (Fig EV2). Both T and B cells were only weakly positive for Nef-GFP-EV. With the exception of neutrophils, the preferential uptake of Nef-GFP-EV by mature myeloid cells and their progenitors is consistent with the broad expansion of these subsets observed in the BM following challenge with Nef-EVs."

6. Mechanistic conclusions exceed the presented evidence.

Although the authors present extensive datasets, the mechanistic link between Nef-containing Evs, metabolic rewiring, chromatin remodeling, and durable inflammatory memory remains largely associative. Interventions targeting glycolysis or cholesterol metabolism provide partial support but do not establish a specific causal pathway connecting Nef to epigenetic reprogramming.

Response to point 4 and 6:

We accept this criticism, and this is now reflected in the paper in (p. 20, lines 2-16). Please also see our response to Referee 1 (comment 1, above).

"The mechanistic studies presented here, while supporting the proposed pathway, remain largely associative, and the precise molecular mechanisms responsible for maintaining Nef-induced TRIM have yet to be defined. The causal contribution of glycolytic reprogramming to TRIM formation was examined *in vitro*, as *in vivo* testing presents fundamental interpretive obstacles. Global inhibition of glycolysis exerts broad effects on systemic metabolism and inflammation through mechanisms independent of trained immunity or the haematopoietic cell populations under study, confounding interpretation of any results obtained. Tissue-specific approaches, such as conditional knockout of GPI1 in myeloid progenitors analogous to the T cell-specific model described by Wu et al. (Wu *et al.*, 2020), face a different problem: because glycolysis is required for basal haematopoietic function, genetic disruption of essential glycolytic enzymes in progenitor cells would be expected to impair survival, proliferation, and engraftment, testing fundamental hematopoietic viability rather than the specific role of metabolic rewiring in immune memory establishment. Also, there are limited evidence for the stability and mechanisms of epigenetic adaptations in haematopoietic progenitors and their causal link to enhanced secondary responses."

Also, we have revised our wording and now introduce the phenotype as "a durable myeloid-biased state consistent with trained immunity."

Minor Comments:

1.The terminology "trained immunity" should be used more cautiously throughout the manuscript and clearly distinguished from long-term inflammatory priming or persistence.

We agree and where appropriate relaced "trained immunity" with "trained innate immunity" to distinguish it from two other phenomena.

2. The discussion would benefit from a more explicit consideration of alternative explanations, particularly indirect systemic effects of EV administration.

We agree with criticism and have expanded the "Limitations" section of the manuscript to discuss this point.

Regarding indirect systemic effects of EV administration specifically, we would note that our experimental design directly addresses this concern through the GFP-EV control. GFP-EVs are produced in the same cell line, under identical conditions, at the same particle number, with comparable size distribution and protein cargo complexity, differing from Nef-EVs only in the identity of the incorporated transgene product and a few host proteins, which are unlikely to influence trained immunity due to species specificity. Any indirect systemic effect of EV infusion, including complement activation, pattern recognition receptor engagement by EV surface components, or bystander cytokine release acting on unexposed haematopoietic progenitors, would be expected to occur equally in GFP-EV-treated animals. Since GFP-EV-treated animals showed no myelopoietic expansion, inflammatory memory, or epigenetic

reprogramming distinguishable from untreated controls, indirect systemic effects cannot account for the phenotypes observed in Nef-EV-treated animals.

Furthermore, the competitive bone marrow transplantation experiments provide an additional and more direct refutation of the indirect systemic effects hypothesis. In these experiments, BM from Nef-EV-exposed donors and untreated donors was co-transplanted into the same recipient animal, which therefore shares an identical systemic environment. The selective expansion of CD45.1+ monocytes and LSKs derived from the Nef-EV-exposed donor, but not of CD45.2+ cells from the untreated donor in the same animal, demonstrates that the observed effects are cell-intrinsic and cannot be attributed to systemic factors present in the recipient.

***3. Several conclusions in the Discussion section would benefit from clearer separation between experimentally supported findings and speculative interpretations*.**

We identified such instances, and revised text according to this recommendation.

Referee #3:

1. A previous study by Dubrovsky et al. (2022) proposed that exNef induces trained immunity, thereby linking this phenomenon to persistent inflammation and HIV-associated comorbidities. The authors of the current study should more clearly emphasize the novelty of their work, as the central hypothesis appears largely overlapping with this prior publication.

We thank the reviewer for raising this point and agree that the relationship between the current study and Dubrovsky et al. (2022) requires clearer articulation in the manuscript. We have revised the discussion accordingly (p. 16, lines 14-24).

Response:

"The findings of this study provide mechanistic insight into our initial observation that Nef-EVs can enhance inflammatory responses in macrophages following a delayed secondary stimulus, consistent with trained immunity (Dubrovsky et al., 2022). In the current study we demonstrate that Nef-EV, can induce long-lasting inflammatory memory in macrophages and their bone marrow progenitors, establishing this as a previously unrecognized mechanism by which HIV drives chronic inflammation. We further demonstrate that Nef-EV-induced reprogramming is characterized by a pronounced and durable expansion of myeloid cells across organ systems, sustained through a cell-intrinsic change in haematopoietic progenitors as shown by competitive bone marrow transplantation. This reprogramming was accompanied by metabolic alterations, shifts in chromatin accessibility, and inflammatory gene expression profiles consistent with TRIM…"

2. ***The authors should also clarify the rationale behind their experimental design, specifically***

the intravenous infusion of extracellular vesicles containing Nef three times per week for two weeks. This approach differs from classical trained immunity mouse models, such as those using β-glucan or BCG, and the reasoning for this dosing regimen and route of administration should be better justified.

Response:

We thank the reviewer for this question and agree that the rationale for our dosing regimen should be stated explicitly in the manuscript. The dosage and regimen are based on our previous study (Mukhamedova *et al.*, 2019), where we investigated the mechanism of inflammation caused by exNef. This is now stated on p. 6, lines 19-20. We discuss the rationale of using this regimen in the text from p. 18, line 18 to page 19, line 8.

"TRIM established after a primary challenge is influenced by events occurring in the interval between the primary and secondary challenges (Sviridov *et al.*, 2025). If inflammation resolves during this period, adaptive TRIM is likely to develop. In contrast, if residual inflammation persists, it may potentiate TRIM-associated inflammatory responses, leading to the formation of maladaptive TRIM. We have previously demonstrated that Nef can cause chronic inflammation (Mukhamedova *et al.*, 2019), and others have shown that, despite effective anti-retroviral therapy, Nef is present in the blood of a large proportion of PLWH at concentrations causing both chronic inflammation and formation of TRIM in model systems (Dubrovsky *et al.*, 2022; Raymond *et al.*, 2011). Unlike β-glucan or BCG, which are administered as single bolus stimuli that mimic discrete infectious exposures, Nef-containing EVs are continuously produced and released into the circulation throughout the course of HIV infection, including during ART-suppressed disease. The repeated low-dose infusion protocol is therefore intended to model this chronic, low-level exposure rather than a single acute training event, and is more physiologically relevant to the clinical context we are studying. It is plausible that, combined with low-level endotoxemia due to gut barrier damage (Deeks *et al*, 2013), continuous exposure to Nef creates conditions favourable to the formation of maladaptive TRIM."

3. *The authors associate the observed transcriptional and epigenetic changes in the myeloid compartment with trained immunity. While these signatures are consistent with previously described trained immunity profiles, definitive functional proof is still lacking. To strengthen this claim, the authors should consider assessing the response of exNef-trained mice to a secondary heterologous challenge and comparing it with appropriate control groups.*

Response:

1. We accept the criticism of some overinterpretation, and this is now reflected in the paper on p. 20, lines 2-14. Please also see our response to Referee 1 (comment 1, above).

"The mechanistic studies presented here, while supporting the proposed pathway, remain largely associative, and the precise molecular mechanisms responsible for maintaining Nef-

induced TRIM have yet to be defined. The causal contribution of glycolytic reprogramming to TRIM formation was examined *in vitro*, as *in vivo* testing presents fundamental interpretive obstacles. Global inhibition of glycolysis exerts broad effects on systemic metabolism and inflammation through mechanisms independent of trained immunity or the haematopoietic cell populations under study, confounding interpretation of any results obtained. Tissue-specific approaches, such as conditional knockout of GPI1 in myeloid progenitors analogous to the T cell-specific model described by Wu et al. (Wu *et al.*, 2020), face a different problem: because glycolysis is required for basal haematopoietic function, genetic disruption of essential glycolytic enzymes in progenitor cells would be expected to impair survival, proliferation, and engraftment, testing fundamental haematopoietic viability rather than the specific role of metabolic rewiring in immune memory establishment."

2. We respectfully note that the functional evidence the reviewer requests has in fact been provided in the current manuscript. Specifically, Figure 6G (now Fig 6I) - Represents exactly the type of secondary heterologous challenge experiment the reviewer describes. Macrophages isolated from mice trained with Nef-EVs and then rested for five days, a washout period designed to eliminate any residual direct effects of EV exposure, were stimulated with LPS as a secondary heterologous stimulus. Nef-EV-trained macrophages produced significantly higher levels of IL-6 compared to GFP-EV-treated controls (Fig. 6I) and exhibited markedly enhanced inflammasome activation as evidenced by an elevated ratio of cleaved caspase-1 p10 in the conditioned medium to full-length pro-caspase-1 p45 in the cell lysate (Figs. 6J, K). This enhanced response to a secondary stimulus that is structurally unrelated to the primary training agent fulfills the defining functional criterion of trained immunity, augmented innate immune responses to a heterologous challenge. To make this more clear we have updated this figure to include schematic diagrams.

4. For the ex vivo training experiments in which bone marrow-derived macrophages (BMDMs) were treated with exNef or control for 48 hours, the authors should clarify why the stimulus was removed only after 48 hours of exposure. Additionally, the effects of LPS as a secondary stimulus on cytokine production (TNF and IL-6) in the absence of silencing constructs are not shown (Figure 4). This control is essential to establish exNef as a bona fide inducer of trained immunity.

Response:

1. The 48-hour timepoint was selected based on our previous study (Mukhamedova et al., 2019), in which we systematically characterized the time- and dose-dependence of Nef-EV uptake and its functional consequences in macrophages; this is now stated on p. 6, lines 19-20. That study demonstrated that 48 hours of exposure is sufficient for maximal intracellular Nef accumulation and for the downstream functional effects on cholesterol metabolism and inflammatory gene expression to become fully established, while remaining within a window that does not induce overt cytotoxicity.

2. In the experiments presented in Figure 4 the silencing of GPI1 was controlled by replacing $siRNA^{GPI1}$ with the scrambled siRNA ($siRNA^{scr}$). This control accounts for any non-specific effects of the transfection procedure itself, and thus represents a more stringent baseline than it would be with un-transfected cells.

5. The authors should also consider discussing the mechanisms underlying the effects of exNef on the bone marrow compartment. Do the authors propose that exNef directly reaches the bone marrow and is sensed by hematopoietic stem and progenitor cells (HSPCs), or is the effect indirect, mediated through systemically induced soluble factors such as cytokines that subsequently act on the bone marrow?

Response:

We agree and discuss this issue in the text from p. 17, line 15 to p. 18, line 4:

"Given that perturbed cholesterol efflux and increased lipid raft abundance have been linked to enhanced myelopoiesis (Murphy et al, 2011; Yvan-Charvet et al, 2010), we propose that these metabolic changes are mechanistically connected to the changes in cell-intrinsic myelopoiesis observed following Nef-EVs exposure. The following findings support the role of Nef in the formation of TRIM: (i) Nef-EVs were taken up by BM cells; (ii) there was a notable similarity between the effect of Nef-EVs on BM cells in vivo and human and murine macrophages in vitro and ex vivo (Dubrovsky et al., 2022; Mukhamedova et al., 2019); (iii) the effects of Nef-EVs and rNef on haematopoiesis in vivo and inflammation in vivo and in vitro where qualitatively similar; (iv) EVs produced by cells expressing inactive variant of Nef, Nef(G2A), did not cause formation of inflammatory memory; (v) proteomics analysis of Nef-EVs did not reveal another probable candidate for TRIM formation. Collectively these findings make it likely that the effects of Nef result from direct interaction of Nef with BM cells, rather than mediated by systemic soluble factors. This is further supported by the transplantation experiments, where TRIM phenotype was associated only with donor cells exposed to Nef-EVs."

Minor Comments:

The statistical methods used are not consistently stated in the figure legends and should be clearly indicated for all figures.

We apologise for this oversight; statistical methods were added to the figure legends where they were missing.

Dear Prof. Sviridov,

Thank you for the submission of your revised manuscript to our editorial offices. I have already forwarded to you the reports I received from the three referees that I asked to re-evaluate your study. Please find them again below.

After going through your preliminary point-by-points response (further revision plan), and after cross-commenting with the other referees (who now fully support publication of the study), I have decided to proceed with the submission. Please address the remaining concerns of referee #1 and #2 in a final revised manuscript (by modifying the title, further discussing limitations and more detailed descriptions of analyses) and in a final detailed point-by-point response, as indicated in your revision plan.

Moreover, I have several editorial requests. Please also provide a p-b-p-response regarding these with your final submission.

Editorial requests:

- Please provide the abstract written in present tense throughout.

- Please order the manuscript sections like this, using only these names:
Title page - Abstract (175 words) - Keywords - Introduction - Results - Discussion - Methods - Data availability section - Acknowledgements (please include here all the funding information) - Disclosure and Competing Interests Statement - References - Figure legends - Expanded View Figure legends

Thus, please remove the section 'Materials and Correspondence'.

- There is an author name discrepancy: It is Alexander R. Pinto on the title page of the manuscript and Alex Pinto in the submission system. Please check and make sure the same and correct name is used.

- Please check again that the number "n" for how many independent experiments were performed, their nature (biological versus technical replicates), the bars and error bars (e.g. SEM, SD) and the test used to calculate p-values is indicated in the respective figure legends (main and EV figures). Please also check that all the p-values are explained in the legend, and that theses fit to those shown in the figure. Please provide statistical testing where applicable. Please avoid the phrase 'independent experiment' but clearly state if these were biological or technical replicates. Please also indicate (e.g. with n.s.) if testing was performed, but the differences are not significant. In case n=2, please show the data as separate datapoints without error bars and statistics. See also:

https://link.springer.com/journal/44319/submission-guidelines#cms-Figure-and-data-presentation

If n<5, please show single datapoints for diagrams. Presently, some diagrams have no statistics (e.g. 5B and 5C) or show only partly statistics or miss the 'n.s.' (e.g. 1C, 1D, 1E, 1M, 5D, 5F, 5H, 5I, 5J, 5K, 5L, 5M, 6B, 6C, EV4B, EV4D, EV5E, EV5F and EV5H). Please provide complete statistics as indicated. Moreover:
- Please note that the exact p values are not provided in the legends of figures 4B, C, D, E, F, G.
- Please indicate the statistical test used for data analysis in the legends of figures 2E, F, G, I.
- Please note that information related to n is missing in the legends of figures 3D-F; 6I, K; EV3 A-J.

- Please upload the Reagents & Tools table only as separate file and remove it from the manuscript text file. Please add callouts to the table where appropriate.

- If live virus strains were used in the study, please add a paragraph to the methods section (titled 'Biosafety') providing details on where and how biosafety-relevant experiments were performed and that these were approved, and by whom (institution, government).

- There is presently one EV table uploaded. I think this is a dataset. Please name this 'Dataset EV1' and upload it with its legend on the first TAB as a dataset file. Please update all callouts.

- Figure 6J (in particular upper panel - ATPase) and EV1C, although they have a high megabyte, contain highly pixelated blots. Please provide these in higher resolution.

- Thanks for providing the source data. Please upload this as one folder per main figure, grouping together all the files for this figure (and ZIPed together) and one folder with the source data for the EV figures, containing folders for each EV figure (with the correct nomenclature - Figure EVx), ZIPed together.

In addition, I would need from you uploaded separately:
- a short, two-sentence summary of the manuscript (not more than 35 words).

- two to four short (!) bullet points highlighting the key findings of your study (two lines each).
- a schematic summary figure as separate file that provides a sketch of the major findings (not a data image) in jpeg or tiff format (with the exact width of 550 pixels and a height of not more than 400 pixels) that can be used as a visual synopsis on our website.

I look forward to seeing the further revised version of your manuscript when it is ready. Please let me know if you have questions regarding the revision.

Best,

Achim Breiling
Senior Editor
EMBO Reports

Referee #1:

The authors did a good job in addressing my previous comments by adding new experimental data and textual clarifications. I only have two more minor comments

1) The manuscript title implicates that experiments were conducted in the context of HIV-1 infection, which is not correct expect for the new experiments with EVs from PLWH. A modified title reflecting the use of Nef containing EVs rather than an infection context might be more suitable.
2) Please include the explanation on the conservation of this Nef activity among different Nef variants provided in the rebuttal letter in reply to my last comment also in the manuscript text.

Referee #2:

The authors have expanded several sections of the manuscript, and the revised text is clearer and more cautious in tone. However, several of the major concerns raised previously remain unresolved at the level of experimental evidence, and the newly added data do not fully address the mechanistic or methodological weaknesses identified in the first review. Below I outline the principal outstanding issues.

Cross species EV use and specificity controls
The central concern regarding the use of human SupT1 derived EVs in mice remains insufficiently addressed. The authors argue that GFP EVs represent an adequate control for xenogeneic effects; however, this does not resolve the issue. Nef expression substantially alters EV composition - including proteins, lipids, and RNA - and the authors' own proteomics data identify >100 proteins uniquely present in Nef EVs. Given the relatively modest effect sizes in several assays, the possibility that xenogeneic or transfection induced changes in EV cargo contribute to the phenotype remains a significant concern. It is notable that primary human EVs can elicit mixed lymphocyte reaction like responses when added to allogeneic PBMCs in vitro, underscoring the risk of non specific cross species activation in vivo.
At minimum, the effects should be controlled using defined functional Nef mutations. The myristoylation deficient Nef(G2A) mutant serves only as a control for EV incorporation; it is not an appropriate control for Nef function. G2A Nef fails to localize to membranes, is excluded from EVs, and is structurally perturbed. As such, it cannot discriminate whether the observed effects depend on canonical Nef effector motifs (e.g., PxxP/SH3 binding domain, di leucine motif, acidic cluster) or on EV associated versus soluble signalling. Consequently, the current data do not exclude the possibility that the reported phenotypes arise from Nef independent alterations in EV cargo.
In summary, the combination of xenogeneic EVs and the absence of functional Nef mutants substantially weakens the mechanistic conclusions drawn in the manuscript.
Biodistribution and target cell engagement in vivo
The newly added ex vivo uptake assay shows that bone marrow cells can internalize Nef EVs in culture. However, this does not address the original concern:
whether intravenously injected EVs reach the bone marrow in vivo, at what efficiency, and with what cellular tropism.
Without in vivo tracking or biodistribution experiments, the possibility remains that the in vivo effects are indirect and mediated by circulating cytokines or peripheral immune activation.

MISEV compliance and EV purity
The added characterization improves the dataset but does not achieve MISEV recommended rigor:
Aside from the fact, that the description of vesicle purification is very cryptic, EV preparations without SEC and subsequent gradient purification are certainly not free of non vesicular Nef or other contaminating proteins. This is worrisome for in-vivo injection experiment. Dosing still relies on NTA and protein content rather than multi parameter normalization.
Given that the mechanistic claims depend entirely on EV associated Nef, higher stringency purification and characterization

remain necessary.
Mechanistic overextension
The revised discussion adopts more cautious language, which is appropriate.
Nevertheless, No direct epigenetic marks are shown (e.g., H3K4me3, H3K27ac at relevant loci), no causal perturbation of chromatin modifying pathways is provided. The metabolic intervention is limited to in vitro partial glycolytic inhibition.
Thus, while the data are consistent with innate immune priming, they do not yet establish trained immunity mechanistically.

Recommendation
The manuscript has improved, but several essential controls and mechanistic clarifications are still lacking. The conclusions remain stronger than the data support, particularly regarding:
-the specificity of Nef action,
-exclusion of xenogeneic or cargo composition confounders,
-the mechanistic link to trained immunity, and
-in vivo EV targeting of bone marrow progenitors.

These points are central to the manuscript's core claims and must be addressed experimentally before the work is suitable for publication in EMBO Reports.

Referee #3:

The authors have satisfactorily addressed all of my comments, and the revisions have significantly improved the clarity and quality of the manuscript.

BAKER
HEART & DIABETES INSTITUTE

Dr. Achim Breiling

Senior Editor, *EMBO Reports*

Melbourne, May 31, 2026

Submission ID: EMBOR-2025-63451V1

Dear Dr. Breiling,

Thank you very much for your letter of May 20th, 2026, and careful review of our manuscript entitled "**HIV Infection Generates Lasting Innate Immune Memory in Hematopoietic Stem and Progenitor Cells In Vivo"** (Submission ID EMBOR-2025-63451V1). Please note that in response to the comment of Referee 1 the title of the paper has been amended to "HIV-1 Nef Generates Lasting Innate Immune Memory in Haematopoietic Stem and Progenitor Cells In Vivo".

We appreciate the referees' comments and are delighted that two of them recommended accepting the paper for publication. Attached to this submission please find a detailed point-by-point response to the minor recommendations of Referee 1, our detailed responses to the comments of Referee 2, and description of changes made to the manuscript in response to Editorial recommendations. We believe that we have fully addressed all comments of the Editor and the reviewers.

Sincerely yours,

Dmitri Sviridov

Andrew Murphy

Baker Heart and Diabetes Institute

75 Commercial Road, Melbourne, Vic 3004

T +61 3 8532 1111 E reception@baker.edu.au W baker.edu.au

100 YEARS

Point-by-point response to the comments of the reviewers and the Editor

Referee Comments

Referee #1

The authors did a good job in addressing my previous comments by adding new experimental data and textual clarifications. I only have two more minor comments
1) The manuscript title implicates that experiments were conducted in the context of HIV-1 infection, which is not correct expect for the new experiments with EVs from PLWH. A modified title reflecting the use of Nef containing EVs rather than an infection context might be more suitable.

Response:

We agree and the title has been modified according to this recommendation. Our preferred option was "Extracellular Vesicles Containing HIV Nef Generate Lasting Innate Immune Memory in Haematopoietic Stem and Progenitor Cells In Vivo", however, this is 31 characters longer than mandated length of the title and we had to settle for "HIV-1 Nef Generates Lasting Innate Immune Memory in Haematopoietic Stem and Progenitor Cells In Vivo".

2) Please include the explanation on the conservation of this Nef activity among different Nef variants provided in the rebuttal letter in reply to my last comment also in the manuscript text.

Response:

We agree, and the requested information is now provided at p. 18, lines 12-16:

"Notably, we have previously tested Nef proteins isolated from multiple HIV and SIV variants across a panel of assays underlying pro-inflammatory effects of Nef (Asztalos et al, 2010; Mujawar et al., 2006). In none of these assays did we observe qualitative differences in activity between Nef isolates, suggesting that the observed effects are a conserved property of Nef".

Referee #2

Comment for EMBO Reports (Re Review After Major Revision)

The authors have expanded several sections of the manuscript, and the revised text is clearer and more cautious in tone. However, several of the major concerns raised previously remain unresolved at the level of experimental evidence, and the newly added data do not fully address the mechanistic or methodological weaknesses identified in the first review. Below I outline the principal outstanding issues.

Cross species EV use and specificity controls

The central concern regarding the use of human SupT1 derived EVs in mice remains insufficiently addressed. The authors argue that GFP EVs represent an adequate control for xenogeneic effects; however, this does not resolve the issue. Nef expression substantially alters EV composition - including proteins, lipids, and RNA - and the authors' own proteomics data identify >100 proteins uniquely present in Nef EVs. Given the relatively modest effect sizes in several assays, the possibility that xenogeneic or transfection induced changes in EV cargo contribute to the phenotype remains a significant concern. It is notable that primary human EVs can elicit mixed lymphocyte reaction like responses when added to allogeneic PBMCs in vitro, underscoring the risk of nonspecific cross species activation in vivo.

At minimum, the effects should be controlled using defined functional Nef mutations. The myristoylation deficient Nef(G2A) mutant serves only as a control for EV incorporation; it is not an appropriate control for Nef function. G2A Nef fails to localize to membranes, is excluded from EVs, and is structurally perturbed. As such, it cannot discriminate whether the observed effects depend on canonical Nef effector motifs (e.g., PxxP/SH3 binding domain, di leucine motif, acidic cluster) or on EV associated versus soluble signalling. Consequently, the current data do not exclude the possibility that the reported phenotypes arise from Nef independent alterations in EV cargo.
In summary, the combination of xenogeneic EVs and the absence of functional Nef mutants substantially weakens the mechanistic conclusions drawn in the manuscript.

Response:
1. The reviewer raises an important point regarding the possibility that Nef expression alters EV composition beyond incorporation of Nef itself, potentially contributing indirectly to the observed phenotype. There is no doubt that Nef affects both the quantity and molecular composition of EVs produced by Nef-expressing cells, making possible contribution of various EV constituents an important issue. However, immune signalling pathways have limited cross-reactivity across species, and using EVs produced in human cells restricts nonspecific biological activity of EV cargo in mice reducing the likelihood of non-Nef EV constituents contributing to immune responses. Further, bearing in mind a possibility that there are always exceptions, and “something” in human EVs modified by Nef may activate the innate immune system in mice, we compared protein composition of EVs produced by human cells with and without Nef – and found only four proteins linked to innate immunity. All these proteins are specific to human cells and do not work on mouse receptors. We also tested recombinant Nef and found similar effects on BM in the absence of other EV constituents. Furthermore, EVs produced by cells expressing NefG2A and not incorporating Nef (as NefG2A is not incorporated into EVs) had no effect on HSPCs. All these controls are described in the paper on p. 18. We do not believe that extending this body of evidence to testing Nef-EVs produced by murine cells would add much: such EVs could introduce distinct murine-specific signaling pathways not relevant to the human disease setting. The primary objective in this study was to determine whether Nef is sufficient to induce the observed phenotype in HSPCs and whether Nef-dependent EVs can trigger persistent inflammatory reprogramming in vivo; the effects on bone marrow of non-Nef proteins in EVs are beyond the scope of our study.

2. Responding to the criticism of the choice of Nef mutant, we agree that G2A mutation incapacitates most Nef functions, and this limits interpretation. However, “milder” mutations, which affect a particular function of Nef, such as the ones suggested by the reviewer, require understanding of how this function affects capacity of Nef to elicit TRIM. In effect, choosing the right mutation requires prior structure-function analysis of Nef sequence in relation to formation of TRIM. This falls beyond the scope of our study.

Biodistribution and target cell engagement in vivo

The newly added ex vivo uptake assay shows that bone marrow cells can internalize Nef EVs in culture. However, this does not address the original concern: whether intravenously injected EVs reach the bone marrow in vivo, at what efficiency, and with what cellular tropism. Without in vivo tracking or biodistribution experiments, the possibility remains that the in vivo effects are indirect and mediated by circulating cytokines or peripheral immune activation.

Response:

Although lack of data on the EV uptake by BM in vivo is a limitation, the conclusions of the study are supported by several lines of evidence. Our experiments show that systemically applied Nef-EVs affect many readouts in bone marrow cells. Competitive BMT shows that only cells exposed to Nef are affected. In our view, this result makes it unlikely that the effects on BM could be secondary, e.g. mediated by cytokines secreted by cells outside BM. Combining these data with evidence from literature showing that exosomes do reach BM delivering their cargo to BM cells and that functional response of BM cells accurately reflects this delivery (e.g. Kfoury et al, 2021, Cell Stem Cell 28, 2090–2103, or Gao et al, Theranostics, 2019, 28;9:5642–5656), we do not believe that additional evidence is required to prove that Nef-EVs reach BM cells. The following sentence has been added to the text at p. 7, lines 17-20:

"This is consistent with the findings of Kfoury et al (Kfoury *et al*, 2021) showing that EVs reach and deliver their cargo to BM cells, and that functional response from BM accurately reflects this delivery."

MISEV compliance and EV purity

The added characterization improves the dataset but does not achieve MISEV recommended rigor: Aside from the fact, that the description of vesicle purification is very cryptic, EV preparations without SEC and subsequent gradient purification are certainly not free of non vesicular Nef or other contaminating proteins. This is worrisome for in-vivo injection experiment. Dosing still relies on NTA and protein content rather than multi parameter normalization. Given that the mechanistic claims depend entirely on EV associated Nef, higher stringency purification and characterization remain necessary.

Response:

Our EV characterisation is fully compliant with minimum requirements recommended by MISEV. The description of EV purification was brief because we refer to the previous paper where this procedure was described in detail; we now replaced this with a more detailed description of EV purification (see p. 28, lines 7-19). While non-EV Nef may be present in the Nef-EV preparations, our results show that a 50-fold higher concentration of free Nef is required to achieve an effect similar to that of Nef-EVs, making this concern irrelevant. We also compared our EV purification method with other techniques, including Norgen Biotek columns, considered a gold standard, and found that "our" method combines purity that is the same or better than that achieved by other methods while losses are minimized. Finally, if there are impurities (e.g. proteins attached to the EV surface), they would be similar for Nef-EVs and GFP-EVs, i.e. are properly controlled. The scope of our study was focused on Nef and did not extend to relationships between formation of TRIM and structural properties of EV, which would require using orthogonal EV metrics.

Mechanistic overextension

The revised discussion adopts more cautious language, which is appropriate.

Nevertheless, No direct epigenetic marks are shown (e.g., H3K4me3, H3K27ac at relevant loci), no causal perturbation of chromatin modifying pathways is provided. The metabolic intervention is limited to in vitro partial glycolytic inhibition.

Thus, while the data are consistent with innate immune priming, they do not yet establish trained immunity mechanistically.

Response:

While we did not investigate the epigenetic marks, we present the results of snATAC-seq analysis, which shows clearly that Nef has caused epigenetic reprogramming through changes in chromatin structure/accessibility, which is a functional downstream readout of epigenetic modifications and is widely used to identify persistent regulatory reprogramming associated with trained immunity. In our view, the defining feature of TRIM is the establishment of persistent innate immune memory mediated by stable epigenetic and transcriptional reprogramming, rather than the involvement of any single specific histone modification pathway. Identification of the exact histone modifications and chromatin-modifying enzymes involved would certainly be of interest mechanistically, but we consider this to represent a downstream mechanistic question rather than a prerequisite for defining the presence of trained immunity itself.

As to limited mechanistic evidence for causality of Nef in triggering TRIM in vivo, we admit this limitation and dedicated significant space in the limitation section discussing it.

The following text was added at p. 10, lines 4-9:

"Although we did not investigate the specific mechanism of this reprogramming, the epigenetic marks, the results of snATAC-seq analysis shows that Nef has caused an epigenetic reprogramming through changes in chromatin structure/accessibility, which is a functional readout of epigenetic modification."

<u>Referee #3:</u>

The authors have satisfactorily addressed all of my comments, and the revisions have significantly improved the clarity and quality of the manuscript.

We appreciate the reviewer' positive evaluation of our effort.

<u>Editorial requests</u>

1. *Please provide the abstract written in present tense throughout.*

Done

2. *Please order the manuscript sections like this, using only these names: Title page - Abstract (175 words) - Keywords - Introduction - Results - Discussion - Methods - Data availability section - Acknowledgements (please include here all the funding information)*

- Disclosure and Competing Interests Statement - References - Figure legends - Expanded View Figure legends. Thus, please remove the section 'Materials and Correspondence'.

Done

3. *There is an author name discrepancy: It is Alexander R. Pinto on the title page of the manuscript and Alex Pinto in the submission system. Please check and make sure the same and correct name is used.*

We apologize for this oversight, the data in the submission system are corrected.

4. Statistics
   a. Please check again that the number "n" for how many independent experiments were performed, their nature (biological versus technical replicates), the bars and error bars (e.g. SEM, SD) and the test used to calculate p-values is indicated in the respective figure legends (main and EV figures).

Done

   b. Please also check that all the p-values are explained in the legend, and that theses fit to those shown in the figure.

Done

   c. Please provide statistical testing where applicable. Please avoid the phrase 'independent experiment' but clearly state if these were biological or technical replicates.

Done

   d. Please also indicate (e.g. with n.s.) if testing was performed, but the differences are not significant.

Done

   e. In case n=2, please show the data as separate datapoints without error bars and statistics. See also: https://link.springer.com/journal/44319/submission-guidelines#cms-Figure-and-data-presentation

N/A

   f. If n<5, please show single datapoints for diagrams. Presently, some diagrams have no statistics (e.g. 5B and 5C) or show only partly statistics or miss the 'n.s.' (e.g. 1C, 1D, 1E, 1M, 5D, 5F, 5H, 5I, 5J, 5K, 5L, 5M, 6B, 6C, EV4B, EV4D, EV5E, EV5F and EV5H). Please provide complete statistics as indicated.

Done

   g. Moreover: Please note that the exact p values are not provided in the legends of figures 4B, C, D, E, F, G.

We now provide the exact p values in the figures.

5. Please indicate the statistical test used for data analysis in the legends of figures 2E, F, G, I.

Done

6. Please note that information related to n is missing in the legends of figures 3D-F; 6I, K; EV3 A-J.

Done

7. Please upload the Reagents & Tools table only as separate file and remove it from the manuscript text file. Please add callouts to the table where appropriate.

Done

8. If live virus strains were used in the study, please add a paragraph to the methods section (titled 'Biosafety') providing details on where and how biosafety-relevant experiments were performed and that these were approved, and by whom (institution, government).

No live virus strains were used in this study

9. There is presently one EV table uploaded. I think this is a dataset. Please name this 'Dataset EV1' and upload it with its legend on the first TAB as a dataset file. Please update all callouts.

Done

-

10. Figure 6J (in particular upper panel - ATPase) and EV1C, although they have a high megabyte, contain highly pixelated blots. Please provide these in higher resolution.

Now provided at 300 dpi

11. Thanks for providing the source data. Please upload this as one folder per main figure, grouping together all the files for this figure (and ZIPed together) and one folder with the source data for the EV figures, containing folders for each EV figure (with the correct nomenclature - Figure EVx), ZIPed together.

All done

12. In addition, I would need from you uploaded separately:
    a. a short, two-sentence summary of the manuscript (not more than 35 words).

Done

    b. two to four short (!) bullet points highlighting the key findings of your study (two lines each).

Done

    c. a schematic summary figure as separate file that provides a sketch of the major findings (not a data image) in jpeg or tiff format (with the exact width of 550 pixels and a height of not more than 400 pixels) that can be used as a visual synopsis on our website.

Done

Prof. Dmitri Sviridov
Baker Heart and Diabetes Institute
Lipoproteins and Atherosclerosis
75 Commercial Road
Melbourne, VIC 3004
Australia

Dear Prof. Sviridov,

Thank you for the submission of your final revised manuscript to our editorial offices. I now went through this and your final point-by-point-response and consider the remaining points of the referees and the editorial requests as adequately addressed.

I thus am very pleased to accept your manuscript for publication in the next available issue of EMBO reports. Thank you for your contribution to our journal.

Your manuscript will be processed for publication by EMBO Press. It will be copy edited and you will receive page proofs prior to publication. Please note that you will be contacted by Springer Nature Author Services to complete licensing and payment information.

You may qualify for financial assistance for your publication charges - either via a Springer Nature fully open access agreement or an EMBO initiative. Check your eligibility: https://link.springer.com/journal/44319/how-to-publish-with-us

Should you be planning a Press Release on your article, please notify the production team via this form in order to coordinate publication and release dates: https://forms.cloud.microsoft/e/eH4A8JVP6g

If you have any questions, please do not hesitate to contact the Editorial Office. Thank you for your contribution to EMBO Reports.

Yours sincerely,

Achim Breiling
Senior Editor
EMBO Reports

>>> Please note that it is EMBO Reports policy for the transcript of the editorial process (containing referee reports and your response letter) to be published as an online supplement to each paper. If you do NOT want this, you will need to inform the Editorial Office via email immediately. More information is available here: https://link.springer.com/partners/embo-press/editorial-policies#Peer%20review

EMBO Press Author Checklist

Corresponding Author Name: Dmitri Sviridov
Journal Submitted to: EMBO Reports
Manuscript Number: EMBOR-2025-63451

USEFUL LINKS FOR COMPLETING THIS FORM
The EMBO Journal - Author Guidelines
EMBO Reports - Author Guidelines
Molecular Systems Biology - Author Guidelines
EMBO Molecular Medicine - Author Guidelines

Reporting Checklist for Life Science Articles (updated January
This checklist is adapted from Materials Design Analysis Reporting (MDAR) Checklist for Authors. MDAR establishes a minimum set of requirements in transparent reporting in the life sciences (see Statement of Task: 10.31222/osf.io/9sm4x). Please follow the journal's guidelines in preparing your
Please note that a copy of this checklist will be published alongside your article.

Abridged guidelines for figures
1. Data
The data shown in figures should satisfy the following conditions:
- the data were obtained and processed according to the field's best practice and are presented to reflect the results of the experiments in an accurate and unbiased manner.
- ideally, figure panels should include only measurements that are directly comparable to each other and obtained with the same assay.
- plots include clearly labeled error bars for independent experiments and sample sizes. Unless justified, error bars should not be shown for technical
- if n<5, the individual data points from each experiment should be plotted. Any statistical test employed should be justified.
- Source Data should be included to report the data underlying figures according to the guidelines set out in the authorship guidelines on Data

2. Captions
Each figure caption should contain the following information, for each panel where they are relevant:
- a specification of the experimental system investigated (eg cell line, species name).
- the assay(s) and method(s) used to carry out the reported observations and measurements.
- an explicit mention of the biological and chemical entity(ies) that are being measured.
- an explicit mention of the biological and chemical entity(ies) that are altered/varied/perturbed in a controlled manner.
- the exact sample size (n) for each experimental group/condition, given as a number, not a range;
- a description of the sample collection allowing the reader to understand whether the samples represent technical or biological replicates (including how many animals, litters, cultures, etc.).
- a statement of how many times the experiment shown was independently replicated in the laboratory.
- definitions of statistical methods and measures:
  - common tests, such as t-test (please specify whether paired vs. unpaired), simple $\chi 2$ tests, Wilcoxon and Mann-Whitney tests, can be unambiguously identified by name only, but more complex techniques should be described in the methods section;
  - are tests one-sided or two-sided?
  - are there adjustments for multiple comparisons?
  - exact statistical test results, e.g., P values = x but not P values < x;
  - definition of 'center values' as median or average;
  - definition of error bars as s.d. or s.e.m.

Please complete ALL of the questions below.
Select "Not Applicable" only when the requested information is not relevant for your study.

Materials

Newly Created Materials	Information included in the manuscript?	In which section is the information available? (Reagents and Tools Table, Materials and Methods, Figures, Data Availability Section)
New materials and reagents need to be available; do any restrictions apply?	Not Applicable	

Antibodies	Information included in the manuscript?	In which section is the information available? (Reagents and Tools Table, Materials and Methods, Figures, Data Availability Section)
For **antibodies** provide the following information:<br>- Commercial antibodies: RRID (if possible) or supplier name, catalogue number and or/clone number<br>- Non-commercial: RRID or citation	Yes	Reagents and Tools Table

DNA and RNA sequences	Information included in the manuscript?	In which section is the information available? (Reagents and Tools Table, Materials and Methods, Figures, Data Availability Section)
**Short novel DNA or RNA including primers, probes**: provide the sequences.	Yes	Reagents and Tools Table

Cell materials	Information included in the manuscript?	In which section is the information available? (Reagents and Tools Table, Materials and Methods, Figures, Data Availability Section)
**Cell lines:** Provide species information, strain. Provide accession number in repository **OR** supplier name, catalog number, clone number, and/**OR** RRID.	Yes	Reagents and Tools Table
**Primary cultures:** Provide species, strain, sex of origin, genetic modification status.	Yes	Methods
Report if the cell lines were recently **authenticated** (e.g., by STR profiling) and tested for mycoplasma contamination.	Not Applicable	

Experimental animals	Information included in the manuscript?	In which section is the information available? (Reagents and Tools Table, Materials and Methods, Figures, Data Availability Section)
**Laboratory animals or Model organisms:** Provide species, strain, sex, age, genetic modification status. Provide accession number in repository **OR** supplier name, catalog number, clone number, **OR** RRID.	Yes	Methods
**Animal observed in or captured from the field:** Provide species, sex, and age where possible.	Not Applicable	
Please detail **housing and husbandry conditions**.	Yes	Methods

Plants and microbes	Information included in the manuscript?	In which section is the information available? (Reagents and Tools Table, Materials and Methods, Figures, Data Availability Section)
**Plants:** provide species and strain, ecotype and cultivar where relevant, unique accession number if available, and source (including location for collected wild specimens).	Not Applicable	
**Microbes:** provide species and strain, unique accession number if available, and source.	Not Applicable	

Human research participants	Information included in the manuscript?	In which section is the information available? (Reagents and Tools Table, Materials and Methods, Figures, Data Availability Section)
If collected and within the bounds of privacy constraints report on age, sex and gender or ethnicity for all study participants.	Yes	Methods

Core facilities	Information included in the manuscript?	In which section is the information available? (Reagents and Tools Table, Materials and Methods, Figures, Data Availability Section)
If your work benefited from core facilities, was their service mentioned in the acknowledgments section?	Not Applicable	

Design

Study protocol	Information included in the manuscript?	In which section is the information available? (Reagents and Tools Table, Materials and Methods, Figures, Data Availability Section)
If study protocol has been **pre-registered, provide DOI in the manuscript**. For clinical trials, provide the trial registration number **OR** cite DOI.	Not Applicable	
Report the **clinical trial registration number** (at ClinicalTrials.gov or equivalent), where applicable.	Not Applicable	

Laboratory protocol	Information included in the manuscript?	In which section is the information available? (Reagents and Tools Table, Materials and Methods, Figures, Data Availability Section)
Provide DOI OR other citation details if **external detailed step-by-step protocols** are available.	Not Applicable	

Experimental study design and statistics	Information included in the manuscript?	In which section is the information available? (Reagents and Tools Table, Materials and Methods, Figures, Data Availability Section)
Include a statement about **sample size** estimate even if no statistical methods were used.	Yes	Methods and Figure Legends
Were any steps taken to minimize the effects of subjective bias when allocating animals/samples to treatment (e.g. **randomization procedure**)? If yes, have they been described?	Yes	Methods
Include a statement about **blinding** even if no blinding was done.	Yes	Methods
Describe **inclusion/exclusion criteria** if samples or animals were excluded from the analysis. Were the criteria pre-established?<br><br>If sample or data points were omitted from analysis, report if this was due to attrition or intentional exclusion and provide justification.	Not Applicable	
For every figure, are **statistical tests** justified as appropriate? Do the data meet the assumptions of the tests (e.g., normal distribution)? Describe any methods used to assess it. Is there an estimate of variation within each group of data? Is the variance similar between the groups that are being statistically compared?	Yes	Figure Legends

Sample definition and in-laboratory replication	Information included in the manuscript?	In which section is the information available? (Reagents and Tools Table, Materials and Methods, Figures, Data Availability Section)
In the figure legends: state number of times the experiment was **replicated** in laboratory.	Yes	Figure Legends
In the figure legends: define whether data describe **technical or biological replicates**.	Yes	Methods and Figure Legends

Ethics

Ethics	Information included in the manuscript?	In which section is the information available? (Reagents and Tools Table, Materials and Methods, Figures, Data Availability Section)
Studies involving **human participants**: State details of **authority granting ethics approval** (IRB or equivalent committee(s), provide reference number for approval.	Not Applicable	
Studies involving **human participants**: Include a statement confirming that **informed consent** was obtained from all subjects and that the experiments conformed to the principles set out in the WMA Declaration of Helsinki and the Department of Health and Human Services Belmont Report.	Not Applicable	
Studies involving **human participants:** For publication of **patient photos**, include a statement confirming that consent to publish was obtained.	Not Applicable	
Studies involving experimental **animals**: State details of **authority granting ethics approval** (IRB or equivalent committee(s), provide reference number for approval. Include a statement of compliance with ethical regulations.	Yes	Methods
Studies involving **specimen and field samples:** State if relevant **permits** obtained, provide details of authority approving study; if none were required, explain why.	Not Applicable	

Dual Use Research of Concern (DURC)	Information included in the manuscript?	In which section is the information available? (Reagents and Tools Table, Materials and Methods, Figures, Data Availability Section)
Could your study fall under dual use research restrictions? Please check biosecurity documents and list of **select agents and toxins** (CDC): https://www.selectagents.gov/sat/list.htm	Not Applicable	
If you used a select agent, is the security level of the lab appropriate and reported in the manuscript?	Not Applicable	
If a study is subject to dual use research of concern regulations, is the name of the **authority granting approval and reference number** for the regulatory approval provided in the manuscript?	Not Applicable	

Reporting

The MDAR framework recommends adoption of discipline-specific guidelines, established and endorsed through community initiatives. Journals have their own policy about requiring specific guidelines and recommendations to complement MDAR.

Adherence to community standards	Information included in the manuscript?	In which section is the information available? (Reagents and Tools Table, Materials and Methods, Figures, Data Availability Section)
State if relevant guidelines or checklists (e.g., **ICMJE, MIBBI, ARRIVE, PRISMA**) have been followed or provided.	Not Applicable	
For **tumor marker prognostic studies,** we recommend that you follow the **REMARK** reporting guidelines (see link list at top right). See author guidelines, under 'Reporting Guidelines'. Please confirm you have followed these guidelines.	Not Applicable	
For **phase II and III randomized controlled trials**, please refer to the **CONSORT** flow diagram (see link list at top right) and submit the CONSORT checklist (see link list at top right) with your submission. See author guidelines, under 'Reporting Guidelines'. Please confirm you have submitted this list.	Not Applicable	

Data Availability

Data availability	Information included in the manuscript?	In which section is the information available? (Reagents and Tools Table, Materials and Methods, Figures, Data Availability Section)
Have **primary datasets** been deposited according to the journal's guidelines (see 'Data Deposition' section) and the respective accession numbers provided in the Data Availability Section?	Yes	Data Availabilitry Section and Reagents and Tools Table
Were **human clinical and genomic datasets** deposited in a public access-controlled repository in accordance to ethical obligations to the patients and to the applicable consent agreement?	Not Applicable	
Are **computational models** that are central and integral to a study available without restrictions in a machine-readable form? Were the relevant accession numbers or links provided?	Not Applicable	
If publicly available data were reused, provide the respective **data citations in the reference list**.	Not Applicable	
